# Genomes of the Most Dangerous Epidemic Bacteria Have a Virulence Repertoire Characterized by Fewer Genes but More Toxin-Antitoxin Modules

**DOI:** 10.1371/journal.pone.0017962

**Published:** 2011-03-18

**Authors:** Kalliopi Georgiades, Didier Raoult

**Affiliations:** Unité de Recherche en Maladies Infectieuses Tropicales Emergentes (URMITE), CNRS-IRD UMR 6236, Faculté de la Médecine, Université de la Méditerranée, Marseille, France; The University of Maryland, United States of America

## Abstract

**Background:**

We conducted a comparative genomic study based on a neutral approach to identify genome specificities associated with the virulence capacity of pathogenic bacteria. We also determined whether virulence is dictated by rules, or if it is the result of individual evolutionary histories. We systematically compared the genomes of the 12 most dangerous pandemic bacteria for humans (“bad bugs”) to their closest non-epidemic related species (“controls”).

**Methodology/Principal Findings:**

We found several significantly different features in the “bad bugs”, one of which was a smaller genome that likely resulted from a degraded recombination and repair system. The 10 Cluster of Orthologous Group (COG) functional categories revealed a significantly smaller number of genes in the “bad bugs”, which lacked mostly transcription, signal transduction mechanisms, cell motility, energy production and conversion, and metabolic and regulatory functions. A few genes were identified as virulence factors, including secretion system proteins. Five “bad bugs” showed a greater number of poly (A) tails compared to the controls, whereas an elevated number of poly (A) tails was found to be strongly correlated to a low GC% content. The “bad bugs” had fewer tandem repeat sequences compared to controls. Moreover, the results obtained from a principal component analysis (PCA) showed that the “bad bugs” had surprisingly more toxin-antitoxin modules than did the controls.

**Conclusions/Significance:**

We conclude that pathogenic capacity is not the result of “virulence factors” but is the outcome of a virulent gene repertoire resulting from reduced genome repertoires. Toxin-antitoxin systems could participate in the virulence repertoire, but they may have developed independently of selfish evolution.

## Introduction

The virulence of pathogenic bacteria has been attributed to virulence factors, and pathogenic bacteria are considered to be better armed compared to bacteria that do not cause disease [1]. In support of this hypothesis, the deletion of genes in pathogens has a detrimental effect on their fitness and on their ability to cause diseases [2]. In contrast, comparative genomic studies have revealed that in some cases, the genomes of bacteria, such as *Rickettsia* or *Mycobacteria* spp. [3]–[5], are reduced [4], [6]–[10]. For example, the genomes of *Mycobacterium leprae*, *Yersinia pestis* and *Salmonella Typhi* contain hundreds of degraded genes. The evolution of specialized bacteria, including pathogenic bacteria, consists mainly of gene losses [10]. Moreover, extreme genome decay is often accompanied by a low GC% content [11]. Furthermore, genes that encode “virulence factors” are also found in the genomes of non-pathogenic bacteria [11], [12], such as free-living bacteria, which may carry more “virulence factors” than do pathogenic bacteria. By counting the number of genes involved in transcription, host-dependent bacteria (including pathogens) were found to have significantly fewer transcriptional regulators than free-living bacteria [10].

A neutral approach to comparative genomic studies is needed to examine all of the previously described parameters that play a role in pathogenicity. The present study was conducted based on this approach and was applied to the genomes of the 12 most dangerous pandemic bacteria (“bad bugs”) of all times for humans; they were compared to their closest non-pathogenic or non-epidemic related species (“controls”). By neutralizing the bias of the observation, we aimed to identify genome specificities associated with the virulence capacity of pathogenic bacteria. We also determined whether virulence is dictated by rules, or if it is the result of individual evolutionary histories.

Currently there is no any official scientific name to describe specifically the most dangerous pandemic bacteria of all times. We therefore suggest the term “bad bugs” to avoid confusion between the epidemic, less pathogenic and non pathogenic species used in this study.

## Methods

The following “bad bugs” were used: *Mycobacterium leprae* TN (NC_002677), *Mycobacterium tuberculosis* H37Rv (NC_000962), *Rickettsia prowazekii* Madrid E (NC_000963), *Corynebacterium diphtheriae* NCTC 13129 (NC_002935), *Treponema pallidum pallidum* SS14 (NC_010741), *Yersinia pestis* KIM (NC_004088), *Bordetella pertussis* Tohama 1 (NC_002929), *Streptococcus pneumoniae* G54 (NC_011072), *Streptococcus pyogenes* M1 GAS (NC_002737), *Salmonella Typhi* CT18 (NC_003198), *Shigella dysenteriae* Sd197 (NC_007606) and *Vibrio cholerae* O395 (NC_009457). For the “controls”, we constructed a 16s RNA phylogenetic tree for each group of species. The following 12 related bacterial species were used: *Mycobacterium avium* 104 (NC_008595), *Mycobacterium smegmatis* MC2 155 (NC_008596), *Rickettsia africae* ESF-5 (NC_012633), *Corynebacterium glutamicum* R (NC_009342), *Treponema denticola* ATCC 35405 (NC_002967), *Yersinia pseudotuberculosis* IP 32953 (NC_006155), *Bordetella bronchiseptica* RB50 (NC_002927), *Streptococcus agalactiae* 2603V/R (NC_004116), *Streptococcus suis* 05ZYH33 (NC_009442), *Salmonella Schwarzengrund* CVM19633 (NC_011094), *Escherichia coli* HS (NC_009800) and *Vibrio parahaemolyticus* RIMD 2210633 (NC_004603).

### Genomic characteristics

All of the genomic characteristics used herein (genome size, GC% content, number of open reading frames, ORFs, number of pseudogenes) were obtained from the NCBI database. Each characteristic was represented graphically, and a Mann-Whitney test [13] was used to identify significantly different “bad bugs” and “control” species. The species were compared in pairs. The number of virulence factors for our species were obtained through literature searches [12]. We searched for genes encoding eukaryotic-like motifs such as ankyrin repeats (ANK), tetratricopeptide repeats (TPR), leucine-rich repeats (LRR), and U- and F- box domains in each of our selected bacterial species using the Simple Modular Architecture Research Tools database (SMART) [14], [15] and the InterPro database [16]; the number of protein secretion systems was evaluated (http://www.ncbi.nlm.nih.gov/sites; http://blast.ncbi.nlm.nih.gov/Blast.cgi). We identified putative small RNAs (sRNAs) using the Rfam database (http://www.sanger.ac.uk/Software/Rfam/) [17]. The ribosomal operon sequences of each of the 24 species were aligned in pairs using ClustalW (http://www.ebi.ac.uk/Tools/clustalw2/index.html) to identify intervening sequences (IVS) for each pair [18]; the number of tandem repeat sequences in each of the 24 species was calculated using the Tandem Repeats Finder platform (http://minisatellites.u-psud.fr/Tandem) [19]. The number of poly (A) tails containing more than five adenine bases was calculated for the evaluated species using a custom algorithm. The Bravais-Pearson correlation coefficient was used to determine whether the number of poly (A) tails was statistically related to the GC% content. Text-mining searches were conducted in the GenBank protein database for the seven following type II toxin-antitoxin (TA) families: VapB/C, RelE/B, ParE/D, MazE/F, phd/doc, ccdA/B and higA/B. Each protein was used in a BLASTN query, and hits were defined based on an e-value threshold of 10e-5 with more than 30% identity and at least 70% coverage.

A principal component analysis (PCA) was performed using the R package for statistical computing (www.r-project.org/).

We analyzed the presence or absence of every gene in each of the 23 COG functional categories from the NCBI database. The species were compared in pairs, i.e., “bad bug” vs. control. To visualize the presence and absence of each gene in every species, each category was represented by a microarray using MeV software (http://www.tm4.org/mev) [20], [21]. Only entire genes were used in this portion of the study; split or degenerated genes were not considered. The same software was used to construct phylogenomic trees based on a presence/absence matrix for each functional category.

For all of the genes that appeared to be specific, a phylogenetic tree was constructed using Mega4 software [22], and the possibility of gene acquisition through horizontal transfer was tested. A GC% content comparison was also performed as a complementary study to phylogenetic trees. For genes present only in the “bad bugs” or only in the controls, the possible protein-protein interactions were identified using the STRING database (http://string.embl.de/newstring_cgi/show_input_page.pl?UserId=EOVfAJha6h_n&sessionId=QQtQlOKZCaj_) to evaluate whether several missing genes belonged to the same network [23].

We tested the presence or absence of the set of 100 genes lost in obligate intracellular bacteria [10] in our 24 bacterial species using a tblastn search in NCBI (http://blast.ncbi.nlm.nih.gov/).

## Results

### Genomic comparison

The genome sizes of the “bad bugs” were significantly inferior to those of their controls (p = 0.0009) (**Table 1**), and the greatest differences were observed in *Mycobacteria* spp. In fact, the genomes of *M. leprae* and *M. tuberculosis* were smaller than their related species, reaching 2,206,288 and 2,576,677 base pairs, respectively. Moreover, the controls had significantly more ORFs (p = 0.004); *M. avium* had 3515 more ORFs than *M. leprae*. The GC% content and the percentage of coding sequences were not significantly different between the two groups (p = 0.57 and p = 0.15 respectively), even though these percentages were often smaller in the “bad bugs”. The most important difference in GC% content was observed in *M. leprae* and its relative *M. avium*, which demonstrated percentages of 57% and 68%, respectively (**Supporting Information S1**). *M. leprae*, *M. tuberculosis*, *S. Typhi*, *S. pyogenes* and *V. cholerae* had a greater number and percentage of poly (A) tails compared to their controls (**Table 2**). This difference was statistically significant (p = 0.0001). A correlation test between the poly (A) tails and the GC% content revealed that these two parameters were strongly correlated (r =  −0.7738). Concerning the “virulence factors”, “bad bugs” demonstrated significantly fewer “virulence factors” than did controls (p = 0.0091) (**Supporting Information S1; Supporting Information S2**). We focused our research on the virulence factors described by Audic *et al*. [12] including two-component systems that represent 30% of the putative virulence factors. Such systems consist of a sensor histidine kinase and a response regulator. We also looked for autotransporter proteins usually used by gram-negative bacteria to deliver large-size virulence factors and for iron uptake proteins [12]. We found that *Y. pestis*, *B. pertussis*, *S. pneumoniae*, *S. dysenteriae* and *V. cholerae* had more eukaryotic-like motifs than did their related control species. However, *M. smegmatis*, *R. africae*, *C. glutamicum*, *T. denticola* and *S. suis* possessed more eukaryotic-like motifs compared to their “bad bug” relatives, but this difference was not statistically significant (p>0.05) (**Supporting Information S1; Supporting Information S2**). We identified all of the protein secretion systems in our 24 bacterial species (type I secretion system, type II secretion system, type III secretion system (component, ATPase, apparatus, lipoprotein, pore protein, chaperone protein, outer membrane pore, low Ca^++^ chaperone, needle protein) and type IV secretion system (ATPase, lipoprotein)). Only epidemic *S. dysenteriae* presented a greater number of protein secretion systems compared to its control, whereas the other controls possessed more protein secretion systems than did their “bad bug” relatives; this difference was statistically significant (p = 0.003) (**Supporting Information S1; Supporting Information S2**). With respect to transposable and selfish elements, more IVSs were present in “bad bugs” than in the controls in four cases. *C. diphtheriae*, *S. pyogenes*, *Y. pestis* and *V. cholerae* had one sequence each that appeared to be an IVS, but their control species did not possess this sequence. *S. Typhi* had five IVSs, whereas its relative *S. Schwarzengrund* possessed six. Finally, *E. coli HS* and *T. denticola* each had two IVSs, whereas their “bad bug” counterparts *S. dysenteriae* and *T. pallidum* had none (**Supporting Information S1; Supporting Information S2**). We also observed a smaller number of ribosomal operons in *C. diphtheriae*, *V. cholerae*, *M. tuberculosis* and *S. pneumoniae* compared to their controls (p = 0.95). *M. tuberculosis*, *S. Typhi*, *S. pyogenes*, *S. pneumoniae* and *V. cholerae* possessed more tandem repeat sequences than did their related species. The remaining “bad bugs” had significantly fewer repeat sequences than did their relatives (p = 0.0001) (**Supporting Information S1; Supporting Information S2**).

**Table 1:** Twelve bacterial couples and their genomic characteristics.

	Bacteria	genome size (bp)	GC%	coding %	ORFs	pseudogenes	phylum
**1**	M. leprae	3, 268, 20	57%	49%	1605	1115	Actinobacteria
	M. avium	5, 475, 491	68%	88%	5120	1143	Actinobacteria
**2**	M. tuberculosis	4, 411, 532	65%	90%	3988	8	Actinobacteria
	M. smegmatis	6, 988, 209	67%	90%	6716	168	Actinobacteria
**3**	R. prowazekii	1, 111, 523	29%	75%	835	0	Proteobacteria
	R. africae	1, 278, 540	32%	72%	1030	87	Proteobacteria
**4**	C. diphtheriae	2, 488, 635	53%	87%	2272	48	Actinobacteria
	C. glutamicum	3, 314, 179	54%	86%	3052	0	Actinobacteria
**5**	T. pallidum	1, 139, 457	52%	93%	1028	9	Spirochetes
	T. denticola	2, 843, 201	37%	91%	2767	19	Spirochetes
**6**	Y. pestis	4, 600, 755	47%	82%	4048	54	Proteobacteria
	Y. pseudotuberculosis	4, 744, 671	47%	82%	3901	73	Proteobacteria
**7**	B. pertussis	4, 086, 189	67%	82%	3436	358	Proteobacteria
	B. bronchiseptica	5, 339, 179	68%	91%	4994	12	Proteobacteria
**8**	S. pneumoniae	2, 078, 953	39%	85%	2115	0	Firmicutes
	S. agalactiae	2, 160, 267	35%	86%	2124	0	Firmicutes
**9**	S. pyogenes	1, 852, 442	38%	83%	1696	35	Firmicutes
	S. suis	2, 096, 309	41%	86%	2186	0	Firmicutes
**10**	S. Typhi	4, 809, 037	52%	83%	4391	205	Proteobacteria
	S. Schwarzengrund	4, 709, 075	52%	85%	4502	152	Proteobacteria
**11**	S. dysenteriae	4, 369, 232	51%	76%	4270	284	Proteobacteria
	E. coli HS	4, 643, 538	50%	86%	4378	95	Proteobacteria
**12**	V. cholerae	4, 132, 319	47%	87%	3875	1	Proteobacteria
	V.parahaemolyticus	45,165,770	45%	86%	4832	0	Proteobacteria

**Table 2:** Number and percentage of poly (A) tails in all bacteria used.

	Bad bugs	poly (A) tails	poly (A) %	poly (A) %	poly (A) tails	Control species
**1**	M. leprae *	1533	0.04	0.01	616	M. avium
**2**	M. tuberculosis *	653	0.01	0.006	481	M. smegmatis
**3**	R. prowazekii	5488	0.49	0.46	5988	R. africae
**4**	C. diphtheriae	2746	0.1	0.09	3021	C. glutamicum
**5**	T. pallidum	2353	0.2	0.6	17510	T. denticola
**6**	Y. pestis	189	0.004	0.18	8845	Y. pseudotuberculosis
**7**	B. pertussis	861	0.02	0.02	1084	B. bronchiseptica
**8**	S. pneumoniae	6906	0.32	0.4	8478	S. agalactiae
**9**	S. pyogenes *	7363	0.4	0.3	6282	S. suis
**10**	S. Typhi *	508	0.01	0.0004	20	S. Schwarzengrund
**11**	S. dysenteriae	506	0.01	0.17	8244	E. coli HS
**12**	V. cholerae *	5676	0.1	0.07	3821	V. parahaemolyticus

Five bad bugs (*) have more poly (A) tails than their controls in number and percentage. The difference is statistically significant (p = 0.0001).

Concerning possible sRNAs, we found that four “bad bugs” had more sRNAs compared to their controls, and five controls had more sRNAs compared to their corresponding “bad bugs” (**Supporting Information S1; Supporting Information S2**). The predictions on sRNA content made with Rfam, based on multiple alignments, are combined with references of previous experimental and computational studies validating these findings [24]–[26].

**Figure 1:**
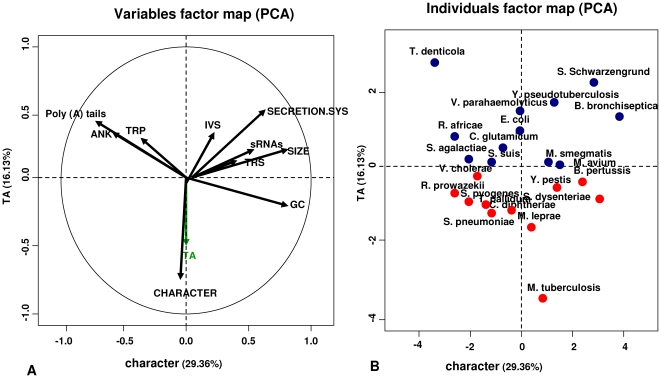
Principal Component Analysis of 11 genomic characteristics. A. Toxin-antitoxin systems (TA) characterize “bad bugs”. B. Bacterial character (“bad bugs”/controls) according to the TA content. The red dots representing the “bad bugs” are positioned separately from the blue dots representing the controls.

We searched the 24 genomes for members of the seven known toxin-antitoxin modules and found that seven “bad bugs” contained significantly more TA systems than did their controls (p = 0.043) (**Table 3** **, Supporting Information S1; Supporting Information S2**). Our results agree with the findings of previous studies [27], [28] and with the data in the TADB (Toxin-Antitoxin Database) [29]. Differences between our results and the TADB are due to the different parameters used for the Blast search [29]. Furthermore, a PCA of these 11 genomic characteristics revealed that the “bad bugs” were characterized by a greater number of TA systems compared to the controls (**Figure 1**).

**Table 3:** Number of toxin-antitoxin in each of the seven known toxin-antitoxin families for the 24 analyzed genomes.

TA families	VapB/C	RelB/E	ParE/D	MazE/F	phd/doc	ccdA/B	higA/B	unclassified	Σ
Species									
M. leprae	-	-	-	-	-	-	-	-	0
M. avium	1	-	-	-	-	-	-	-	1
M. tuberculosis *	38	-	-	-	-	-	-	-	38
M. smegmatis	-	-	-	-	-	-	-	-	0
R. prowazekii	-	-	-	-	-	-	-	-	0
R. africae	2	-	-	4	2	-	2	-	10
C. diphtheriae	-	-	-	-	-	-	-	-	0
C. glutamicum	-	-	-	-	1	-	-	-	1
T. pallidum	-	-	-	-	-	-	-	-	0
T. denticola	1	-	-	-	-	-	-	-	1
Y. pestis *	-	-	-	-	-	-	2	3	5
Y. pseudotuberculosis	-	-	-	-	-	-	-	-	0
B. pertussis	-	-	-	-	-	-	-	-	0
B. bronchiseptica	-	-	-	-	-	-	-	-	0
S. pneumoniae *	-	-	-	-	1	-	1	2	5
S. agalactiae	-	1	1	-	-	-	-	-	2
S. pyogenes *	-	-	1	1	-	-	-	-	2
S. suis	-	-	-	-	-	-	-	-	0
S. Typhi *	2	2	-	-	-	-	2	-	6
S. Schwarzengrund	-	-	-	-	-	-	-	1	1
S. dysenteriae *	-	-	-	2	-	-	-	-	2
E. coli	-	-	-	1	-	-	-	-	1
V. cholerae *	-	2	4	-	-	-	1	6	13
V. parahaemolyticus	-	2	2	-	-	-	-	-	4

Seven “bad bugs” (*) have more TA than “controls”. The difference was statistically significant (p = 0. 043).

**Figure 2:**
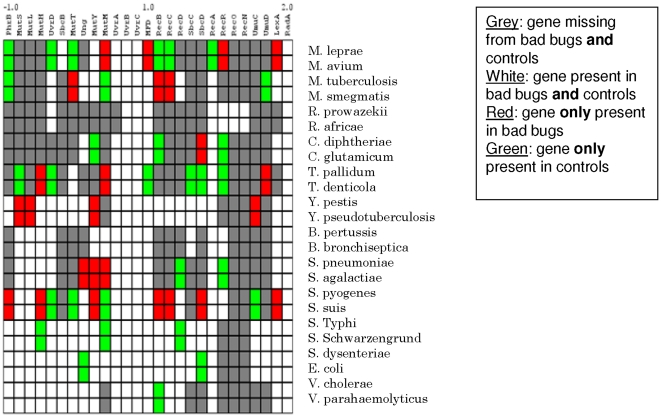
Recombination and repair genes. The “bad bugs” lack recombinational repair genes, whereas the controls lack mismatch excision repair genes.

Ten of the 23 functional categories tested presented a significantly lower number of genes in the “bad bugs” compared to the controls (**Supporting Information S1; Supporting Information S2**). These categories included transcription (p<0.0001), recombination, replication and repair (p<0.0001), signal transduction mechanisms (p<0.0001), cell motility (p = 0.0004), energy production and conversion (p<0.0001), carbohydrate transport and metabolism (p<0.0001), amino acid transport and metabolism (p<0.0001), lipid transport and metabolism (p<0.0001), inorganic ion transport and metabolism (p<0.0001) and secondary metabolite transport and metabolism (p<0.0001). (**Supporting Information S1**). *M. leprae* demonstrated a very significant genome reduction and an important associated difference in gene content for every functional category, as compared to *M. avium*. Similarly, *M. tuberculosis* and *S. dysenteriae* underwent significant genome degradation in most of the functional categories. Analyses of the microarrays revealed the same categories and two more that presented fewer genes in the “bad bugs”: nucleotide transport and metabolism; coenzyme transport and metabolism (**Supporting Information S1**). No statistically significant differences in these categories (p = 0.56 and p = 0.06, respectively) were observed. The 27 repair, replication and recombination genes were separated into the following six categories: direct repair, mismatch excision repair, base excision repair, nucleotide excision repair, recombinational repair and other repair (**Supporting Information S2**). *R. prowazekii* lacked the *rec*B, C, D complex and the DNA mismatch repair enzymes *mut*H, Y, L, S. In contrast, genes involved in the repair of ultraviolet DNA damage, such as *uvr*B and C, the transcription repair coupling factor *MFD*, and the homologous recombination genes *rec*A and *rec*N, were conserved. The *rec*O and *rec*N genes were only found in *Rickettsia* spp. In *M. leprae*, the *rec*A gene and the *rec*B, C, D complex were absent. *S. dysenteriae* only lacked two genes compared to *E. coli HS*, one of which, *Ung*, encodes uracil glycogenase. In general, the “bad bugs” lacked recombinational repair genes, whereas the controls lacked mismatch excision repair genes (**Figure 2**). A phylogenomic tree was constructed for every functional category. In most cases, the phylogenomic trees resembled the phylogenetic tree. However, two trees presented different topologies. For the functional category of cell wall biogenesis genes, *M. smegmatis*, *M. avium*, *T. denticola* and *S. agalactiae* clustered together and *M. avium*, *R. africae*, *C. glutamicum*, *S. suis* and *E. coli HS* clustered together in the phylogenomic tree of defense mechanism genes (**Supporting Information S1**). Furthermore, phylogenetically related species did not cluster together in the phylogenomic tree for virulence factors (**Supporting Information S1**). For all of the genes that were not present in “bad bugs” or controls, a phylogenetic tree was constructed for each respective species pair (**Supporting Information S3**). The two genes trans-2-enoyl-CoA-reductase and thioredoxin reductase were only found in controls and non-pathogenic species; an alignment provided no hits with pathogenic species sequences. However, the four other genes (rhamnolipids biosynthesis-3-oxoloacyl reductase, cinnamoyl-ester-hydrolase, pentachlorophenol-4-monooxygenase and 6-hydroxy-D-nicotine oxidase) were acquired by controls based on horizontal gene transfers (HGT), whereas only one gene (methionin-S-oxide-reductase) was acquired by the “bad bug” *T. pallidum*. Different bacterial members of *β-* and *δ- proteobacteria*, *Firmicutes* and Bacillus, as well as fungi and insects, were found to be possible gene donors. Most of these genes demonstrated an oxidoreductase or a hydrolase activity (**Supporting Information S2**). Seven different protein networks contained genes that were missing in the “bad bugs” in one or more of the pairs. Most of these genes belonged to the functional categories of inorganic ion transport and metabolism, secondary metabolite transport and metabolism, amino acid transport and metabolism and coenzyme transport and metabolism. We also discovered one case in which two genes of *V. cholerae*, which were absent in *V. parahaemolyticus*, were part of the same network (**Supporting Information S2**). All of the other genes of interest were not members of the same networks. Genes that were missing from the controls were mostly transcription and defense mechanism genes, whereas the genes missing in the “bad bugs” demonstrated mostly metabolic and transport functions (**Supporting Information S2**).

Finally, for the set of 100 genes lost in obligate intracellular bacteria [10], 35 of them were unexpectedly found in *M. leprae* and 13 in *T. denticola* (**Supporting Information S2**). Ten of these genes were not found in any of the 24 species assessed in the present study (**Supporting Information S2**). A phylogenomic tree was constructed based on the absence/presence of these genes, and it revealed that there was no convergence in the loss of these genes in the “bad bugs”. Therefore, “bad bugs” cannot be characterized based on the absence or presence of these genes (**Supporting Information S1**).

## Discussion

In our study we wished to compare the 12 most dangerous epidemic bacteria of all times with their phylogenetically closest related species that are non pathogenic or not as dangerous as their epidemic cousins. By doing so, we demonstrated that even when two species are really closely related their evolutionary histories and gene repertoires can differ due to the extreme specialization of one of the two. By using only the closest species as controls, and not more divergent species, we did not bias our results and our findings are eloquent. *R. prowazekii/R. africae* is the only couple with very similar genomic characteristics. However, our choice was based on the fact that *R. prowazekii* is the only dangerous epidemic rickettsial species and *R. africae* is its closest annotated species with the lowest pathogenicity [9]. *R. canadensis* could be another option but its genome is not annotated and is more distant.

Our comparison of “bad bugs” and their respective controls confirmed the findings of previous studies [3]-[9], revealing a significant reduction in the genome size of “bad bugs”. This genome reduction was accompanied by a significant decrease in ORF content, which demonstrates that many genes are progressively disappearing from the genomes of “bad bugs”. The smaller number of ORFs in “bad bugs” results from their host-parasite relationships, which allows them to use the metabolic substrates present in the infected organism. Thus, any enzymes that are essential for the synthesis of such substrates become useless to “bad bugs” [10], [30]-[32]. The mechanism responsible for pseudogenization and the eventual loss of genes is a stepwise process. It begins with a shift toward a higher A + T nucleotide composition and in turn leads to an excess of homopolymers (poly (A) tails). This accumulation leads to gene inactivation, and pseudogenes are eventually removed via large deletions [33]. Indeed, our study showed that some “bad bugs” possessed a larger percentage of poly (A) tails, and these species also demonstrated the smallest coding percentage. Furthermore, if one looks across all low GC% bacteria will not see more poly (A) tails, but when comparing the epidemic species of each couple to the “control” species of the corresponding couple, the species with the lowest GC% (epidemic) have often more poly (A) tails and the two features are significantly correlated. Our work on the recombination and repair system demonstrated that among the studied “bad bugs”, many recombinational repair genes were generally lost. For example, *Rickettsia* spp. have lost all the mismatch excision repair and a big part of the recombinational repair machinery. One interesting feature however, is the gene *rec*O found only in *Rickettsia*. It was recently demonstrated that inactivation of this gene may act as a trigger in the loss of virulence of *R. prowazekii*. Its reactivation in an avirulent strain restored a virulent phenotype [34]. We conclude that in a general manner “bad bugs” have a deficient repair system that renders them incapable of repairing any mutation and of overcoming gene degradation that will eventually lead to pseudogenization and total gene loss. The increased replication error rate leads to faster genome decay and deregulation [34], [35]. In a study focusing on a clade of bacteria that have recently established systematic association with insect hosts it was demonstrated that during the evolution of symbiosis, symbiont genomes typically lack recombination repair genes and have reduced numbers of ribosomal operons [36]. These results are compatible with the punctuated equilibrium theory [37], which postulates that evolution occurs when critical changes in lifestyle lead to the steady and gradual transformation of whole lineages. Defects in the repair machinery of “bad bugs” may explain our results concerning important gene losses in the 10 COG functional categories. The functions of the missing genes are mostly related to metabolic activity, the production of energy and cell motility, and transcription. Intracellular bacteria do not require a large amount of energy because they have no metabolic functions. In addition, most pathogenic bacteria are often completely immobile in the cytoplasm [38]. Twenty-five of the genes that have been lost in “bad bugs” are found in protein-protein interaction networks; eight of them are associated with inorganic ion transport and metabolism, whereas the other seven are related to secondary metabolites, coenzymes and amino acid transport and metabolism. This observation suggests that whole metabolic networks tend to disappear from some “bad bugs”, especially *M. leprae*. This bacterium has lost approximately one-third of the genes involved in metabolism and cellular processes, and about one-fifth of those involved in information functions. Remnants of these once functional genes are found as 1114 pseudogenes within its genome [39]. The *M. leprae* genome presents a remarkable genome reduction that explains why the *M. leprae/M. avium* pair is often differentiated from the other pairs assessed in this study. The phylogenomic trees constructed for each functional category present a topology that differs from the one provided by the phylogenetic trees. Furthermore, the clustering of control species with respect to cell wall biogenesis and defense mechanism trees, or the non-clustering of phylogenetically related species for other tree categories, demonstrate how the gene repertoires of closely related species can possess different histories. Distant species can have a similar gene repertoire due to related evolutionary events. For example, HGT occurs more often in control species than in “bad bugs” that are highly specialized and isolated to a strictly intracellular environment. A recent study demonstrated that host-dependent bacteria favor genome reduction and that the evolution of specialized human pathogens consists mainly of gene loss [10]. This phenomenon of genome reduction is emphasized in our study, because the “bad bugs” are hyperspecialized human pathogens. Taken together, our data confirm that specialized bacteria (“bad bugs”) lose regulation (as their niches are very restricted), repair genes (leading to accelerated gene reduction) and metabolic and energy capabilities linked to their genetic lifestyle.

We noticed that the number of genes identified in the past as “virulence factors” is statistically more important in controls than in “bad bugs”. Furthermore, we investigated the pairs with respect to protein-protein interaction motif-containing proteins and protein secretion systems, which are considered as virulence factors. Eukaryotic-like motifs, such as ANK or TPR repeats, act as signal transducers and transcriptional initiators [40], [41], and therefore, they are considered as important elements in bacterial infection [41]. Our study demonstrated no significant differences in the number of eukaryotic-like motifs between “bad bugs” and controls. Likewise, all the protein secretion systems, due to their roles as communication ports with eukaryotic cells [42], are considered a part of the virulence mechanism comprising the injection of proteins that facilitate bacterial pathogenesis in eukaryotic cells [43]-[48]. Our study revealed that among the “bad bugs”, only *S. dysenteriae* had more secretion system proteins than did its control; the controls generally had significantly more protein secretion systems than did their epidemic relatives. Moreover, IVSs present in bacterial genomes are believed to be in the origin of the pathogenic capacity of some of these organisms, because IVSs result in chromosomal rearrangements or regulatory mutations [11]. In the present study, however, we did not find significantly more IVSs in “bad bugs” compared to their related species. Small RNAs are short RNA transcripts of 100 bp to 300 bp in length and are regulators of transcription factors [49]. They are responsible for the posttranscriptional regulation of gene expression, they also control the transposition of insertion elements, the regulation of the plasmid copy number, they are involved in stress response pathways and they regulate metabolism and transport. Finally, they are believed to have a role in pathogenesis [50], [51]. We found more sRNAs in only four epidemic species and the difference was not statistically significant. So apparently their role in pathogenesis is doubtful for bad bugs. It is necessary for pathogenic bacteria to be well adapted to their hosts to survive in the host environment. Tandem repeat sequences could be in the origin of the phenotypic flexibility of pathogens, thus leading to better host adaptation [52]. A greater number of repeat sequences were found in “bad bugs” in only five of our 12 bacterial pairs. In contrast, in the case of the *M. leprae*/*M. avium* pair, the smallest number of repeats was detected in *M. leprae,* which infects only one host with a very stable environment. Therefore, no additional phenotypic flexibility is required by this bacterium. For the same reason, the number of ribosomal operons is often diminished in highly specialized bacteria [10]. Indeed, we observed a statistically significant difference for *C. diphtheriae*, *V. cholerae*, *M. tuberculosis* and *S. pneumoniae* compared to their related species.

Toxin-antitoxin systems were initially identified as plasmid stabilization factors and then these molecules were also found on chromosomes often in multiple copies and they seem to have a role in the stabilization of integrons in bacterial chromosomes [53]. It has also been proposed that they might be playing a role in protein-expression control especially during starvation periods. [54], [55]. A recent study on *Pseudomonas aeruginosa* showed that TA systems may be used to control the environment of pathogenic bacteria [56]. In fact, TA systems are selfish genes that inhibit detachment of the operon from organisms. They are described as addiction molecules [57]-[59]. Because the two toxin and antitoxin genes are next to each other in the genome, the possibility of eliminating only the toxin and not the antitoxin is limited; any attempt to eliminate the operon consistently leads to the death of the bacterium [60]. Under these conditions, addiction molecules are selected not because they are indispensable to the organism but because the organism cannot be separated from them. This hypothesis has recently been reviewed, and these genes should not be considered as essential [60]. It is interesting to note that the “bad bugs” not only possess a greater number of TA modules compared to controls but also a smaller genome. This finding indicates that the “bad bugs” were incapable of losing their TA modules. A recent study identified active addiction toxins in the *Y. pestis* chromosome [28]. The role of TA systems as “virulence factors” has not yet been elucidated. In a novel study performed in our laboratory, liberation of the toxin into the cytoplasm of infected cells was demonstrated to kill cells via apoptosis [57]. Furthermore, expression of the bacterial toxin by eukaryotic cells initiates apoptotic death [28] or at least inhibits growth especially in yeast cells [61]-[63]. Other than their role in addiction in host bacteria, TA systems could have played a role in bacterial virulence given the pathogenicity initiated after attempts to limit their translation in bacteria presenting such modules.

**Figure 3:**
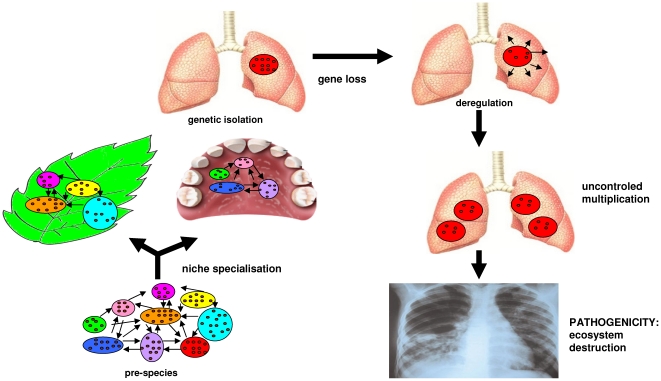
Hyperspecialized pathogenic species evolution. The circles with various colors represent different bacteria; the small brown circles represent the gene repertoire; the arrows around the bacteria represent gene exchange; the leaf, mouth and lungs represent the different potential niches colonized by a species. The red circle is a hyperspecialized bacterium with a decreased gene repertoire *via* gene loss.

Apparently, adaptation does not result in an increase in the complexity of organisms by genome expansion; rather, it is the consequence of a weak, purifying selection [64]. Bacterial species constitute melting pots of strains with different genome repertoires, from which specialized species arise regularly [65]-[68]. Non-specialized species are in fact “pre-species” that enjoy a community lifestyle, which allows them to exchange genes. Such populations can be structured within hosts at microgeographic levels and at larger geographic scales [69]. At some point in their evolution, the organisms specialize in different niches, and subsequently, gene exchanges decrease, and the gene repertoires undergo changes. The specialization of organisms results in gene loss and therefore the loss of regulation genes. Deregulation eventually leads to uncontrolled multiplication, and pathogenicity is demonstrated by destruction of the ecosystem of the organism (**Figure 3**). Therefore, epidemic bacteria are highly specialized species that, following adaptation to their hosts, begin to undergo a genome reduction. Our study suggests that the pathogenic capacity of bacteria is not the result of “virulence factors”. The generic term “virulence factors” is misleading and may be later redefined. Except from toxins that have a direct effect and can constitute in a particular genomic context a possible virulence factor, other factors named “virulence factors” are in reality factors associated to fitness in a tested experimental model [70]. Our analysis showed that specialized, pathogenic bacteria have smaller genomes than non-specialized bacteria. Therefore, it is not possible to say that supplementary virulence factors establish a pathogenic capacity, but that a gene-repertoire is associated to virulence more than specific genes. Furthermore, we showed that all features that are still widely considered as playing a role in virulence are not significantly more abundant in epidemic species. We propose that the term “virulence factor” be abandoned and the following characteristics emphasized: the outcome of a virulent gene repertoire resulting from different evolutionary histories of species, compiling genes (niche adaptation), the lack of others (regulation genes) and the regulation and epigenetic modifications that remain to be described.

## Supporting Information

Supporting Information S1 **Supplementary Figures (S1-S14).**
(PDF)

Supporting Information S2 **Supplementary Tables (S1-S13).**
(PDF)

Supporting Information S3 **Phylogenetic trees of specific genes and HGT.**
(PDF)
