# Supplementary material for: Genomes of the Most Dangerous Epidemic Bacteria Have a Virulence Repertoire Characterized by Fewer Genes but More Toxin-Antitoxin Modules
Source: PLoS One. 2011 Mar 18;6(3):e17962. doi: 10.1371/journal.pone.0017962 (PMC3060909; doi:10.1371/journal.pone.0017962)
Supplement: Supporting Information S1 — Supplementary Figures (S1-S14). (PDF) [file pone.0017962.s001.pdf]

Figure S1 (A ; B):

Graphical representation of the genomic characteristics of "bad bugs" and controls

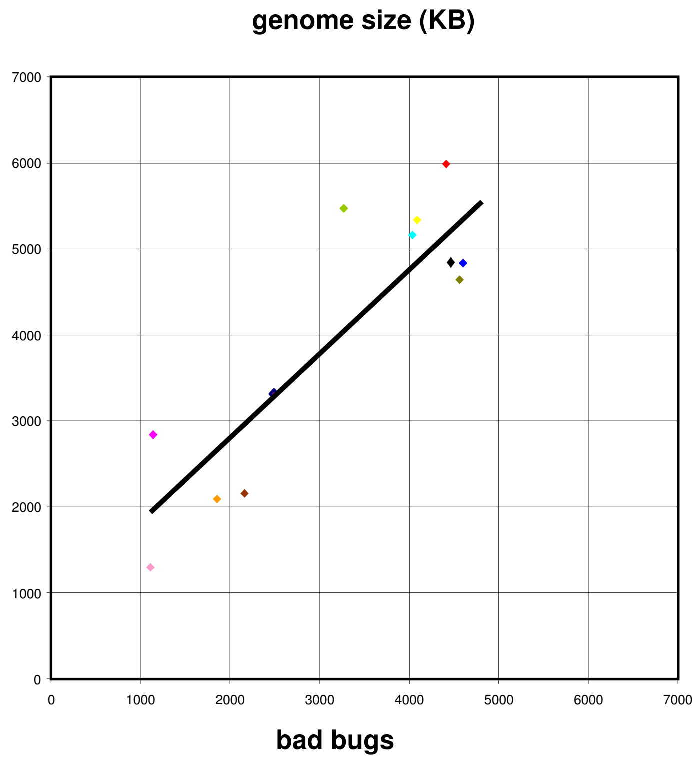

y= 0.6276x + 2240.6
$R^2 = 0.3115$
p value = 0.004

A. Genome size in kilobases

M. leprae/M. avium
M. tuberculosis/M. smegmatis
R. prowazekii/R. africae
C. diphtheriae/C. glutamicum
T. pallidum/T. denticola
Y. pestis/Y. pseudotuberculosis
B. pertussis/ B. bronchiseptica
S. pneumoniae/S. agalactiae
S. pyogenes/S. suis
S. Typhi/ S. Schwarzengrund
S. dysenteriae/E. coli HS
V. cholera/V. parahaemolyticus

y= 0.6276x + 2240.6
$R^2 = 0.3115$
p value = 0.004

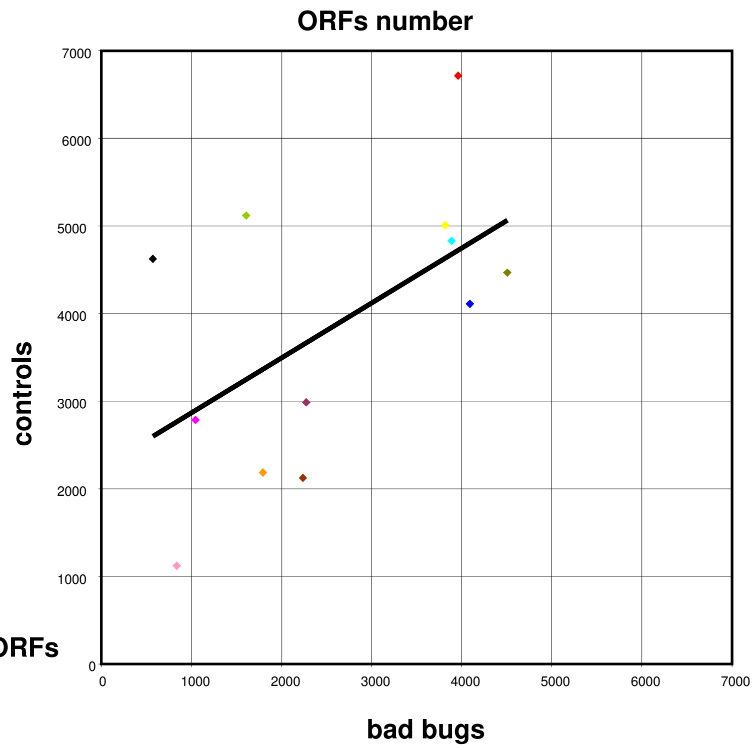

B. Number of ORFs

Figure S1 (C ; D):

Graphical representation of the genomic characteristics of “bad bugs” and controls

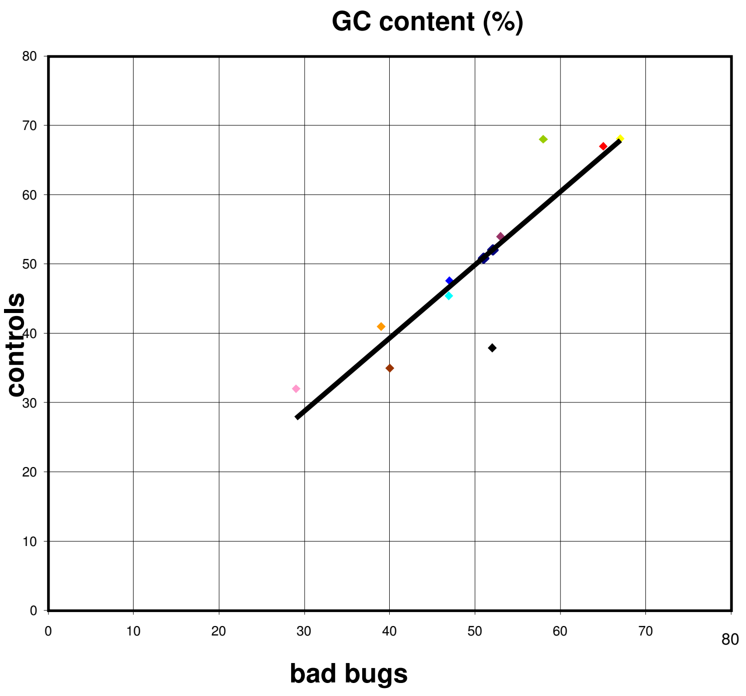

y= 1.0533x – 28.631
$R^2 = 0.8063$
p value =0.5771

C. **GC % content**

M. leprae/M. avium
M. tuberculosis/M. smegmatis
R. prowazekii/R. africae
C. diphtheriae/C. glutamicum
T. pallidum/T. denticola
Y. pestis/Y. pseudotuberculosis
B. pertussis/ B. bronchiseptica
S. pneumoniae/S. agalactiae
S. pyogenes/S. suis
S. Typhi/ S. Schwarzengrund
S. dysenteriae/E. coli HS
V. cholera/V. parahaemolyticus

y = 0.0317x + 84.843
$R^2 = 0.0055$
p value = 0.1533

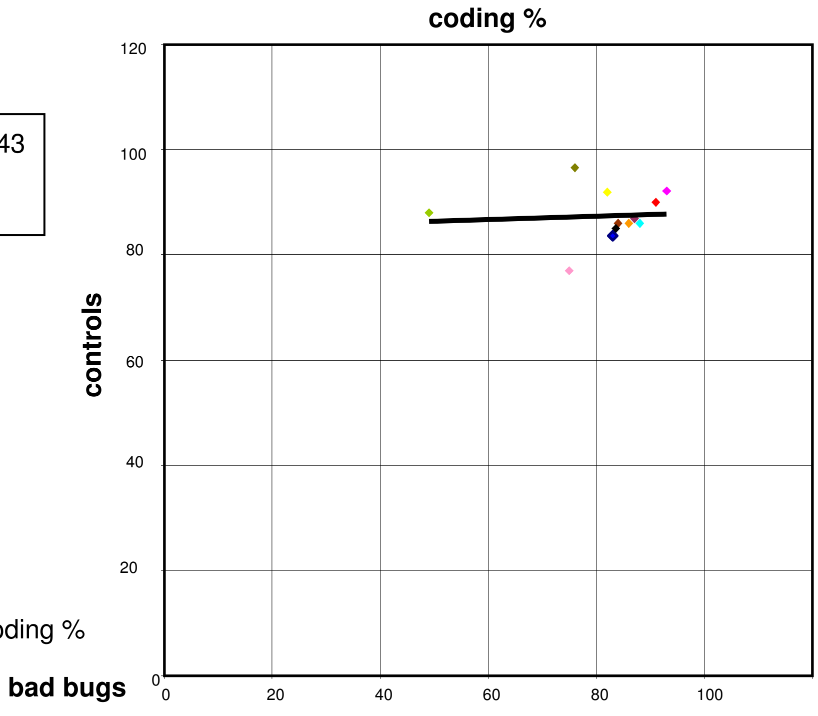

D. Coding %

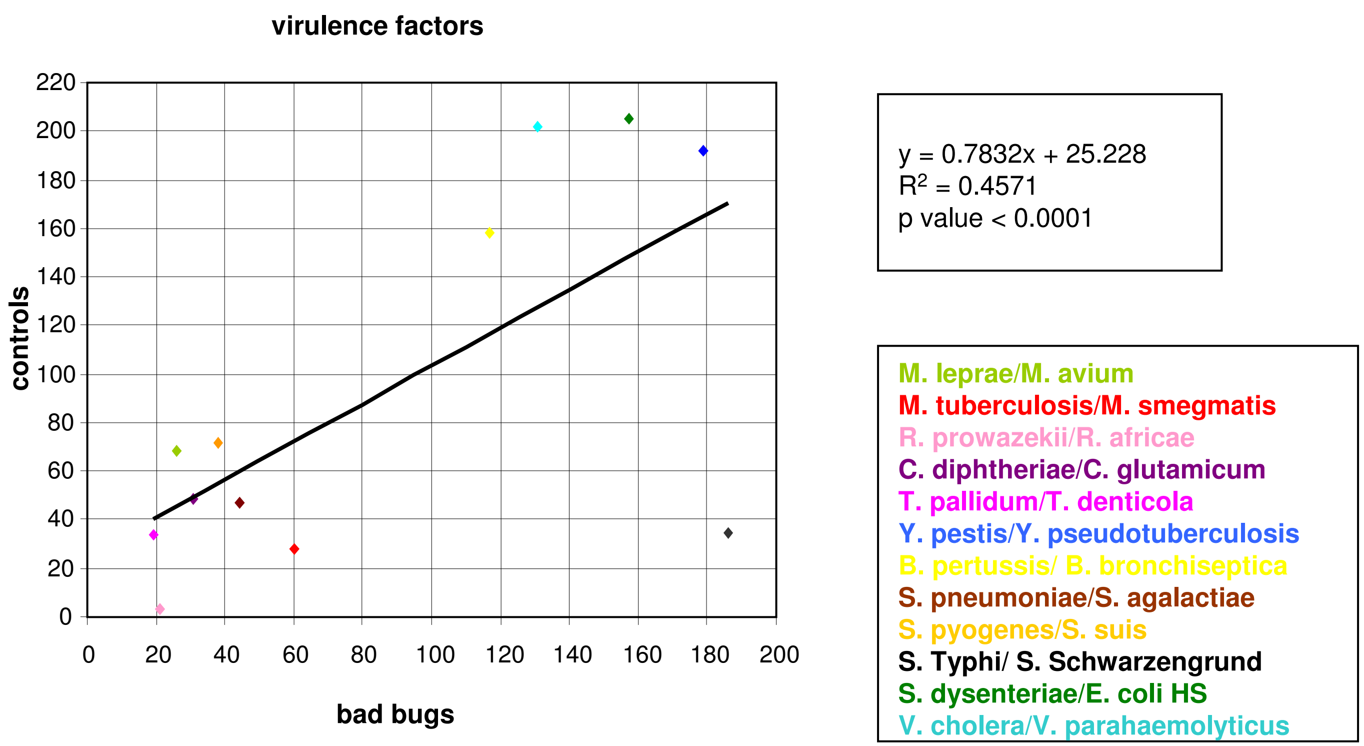

Figure S2:

Graphical representation of the “virulence factor” content

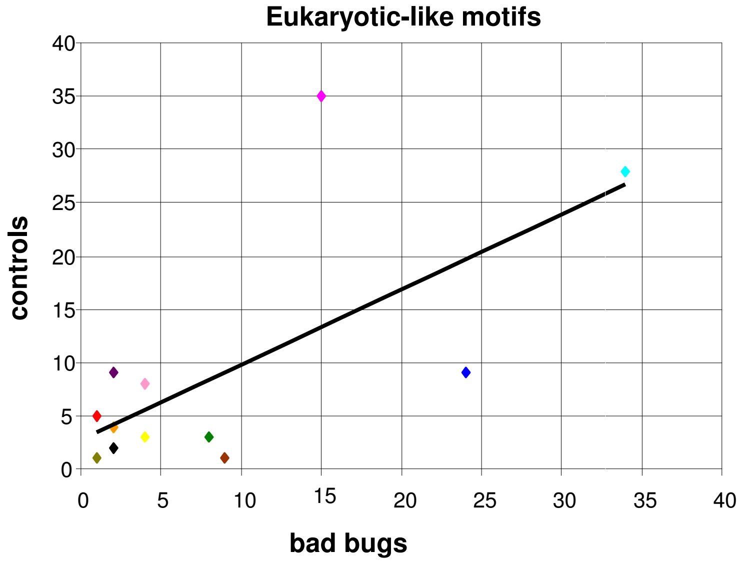

y= 1.7032x + 2.7887
$R^2 = 0.4511$
p value (epidemic more) = 0.1465
p value (non-epidemic more) = 0.8693

M. leprae/M. avium
M. tuberculosis/M. smegmatis
R. prowazekii/R. africae
C. diphtheriae/C. glutamicum
T. pallidum/T. denticola
Y. pestis/Y. pseudotuberculosis
B. pertussis/ B. bronchiseptica
S. pneumoniae/S. agalactiae
S. pyogenes/S. suis
S. Typhi/ S. Schwarzengrund
S. dysenteriae/E. coli HS
V. cholera/V. parahaemolyticus

Figure S3:

Graphical representation of the number of genes encoding eukaryotic-like motifs

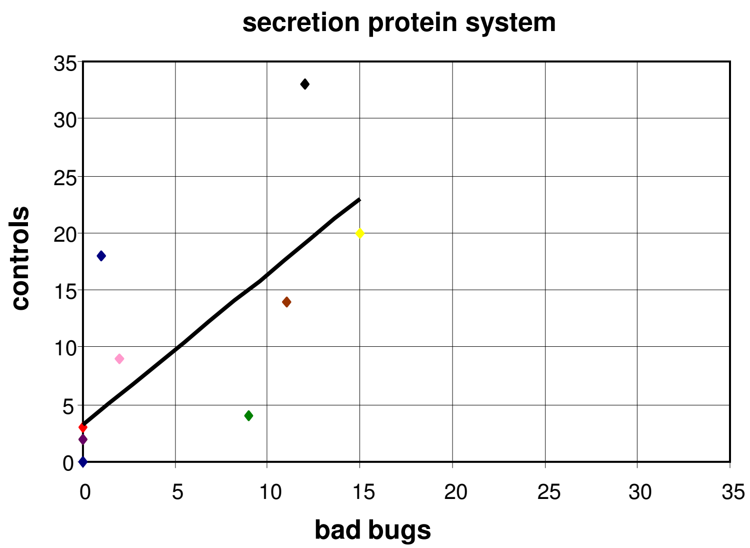

y = 1,3178x + 3.2593
R^2 = 0.5302
p value = 0.0036

M. leprae/M. avium
M. tuberculosis/M. smegmatis
R. prowazekii/R. africae
C. diphtheriae/C. glutamicum
T. pallidum/T. denticola
Y. pestis/Y. pseudotuberculosis
B. pertussis/ B. bronchiseptica
S. pneumoniae/S. agalactiae
S. pyogenes/S. suis
S. Typhi/ S. Schwarzengrund
S. dysenteriae/E. coli HS
V. cholera/V. parahaemolyticus

Figure S4:

Graphical representation of the number of proteins in the secretion systems in the 24 tested bacterial species

Figure S5 (A ; B):

Position of IVSs in "bad bugs" and in controls

```
                       5230      5240      5250      5260      5270      5280
                          |         |         |         |         |         |
C_diphtheriae     -AA-AACTTTCCACCACACACACTA-----AAGCATGGTCCTATCCAG--TATTAGACCC
C_diphtheriae     -AA-AACTTTCCACCACACACACTA-----AAGCATGGTCCTATCCAG--TATTAGACCC
C_diphtheriae     -AA-AACTTTCCACCACACACACTA-----AAGCATGGTCCTATCCAG--TATTAGACCC
C_glutamicum      TAA-GGCTTTCCACACCACCCCTAC-----GGTGGTGTGAATATTCGG--TATTAGACCC
C_glutamicum      TAA-AGCTTTCCACACCACCCCTAC-----GATGATGTGAATATTCGG--TATTAGACCC
C_glutamicum      TAA-AGCTTTCCACACCACCCCTAC-----GATGATGTGAATATTCGG--TATTAGACCC
C_diphtheriae     TAATAACATTGTGTAACGAGCAAAAAAAACAACCATGAACATGTCACCTTTGTTGGGTGA
C_diphtheriae     TAATAACATTGTGTAACGAGCAAAAAAAACAACCATGAACATGTCACCTTTGTTGGGTGA
C_glutamicum      TAACAACA----------------------AACCAATACTGGTTTGG---TGTTGCGCAA
C_glutamicum      TAACAACA----------------------AACCATTA-TGGTTTGG---TGTTGCGCAA
C_glutamicum      TAACAACA----------------------AACCATTA-TGGTTTGG---TGTTGCGCAA
                   **   *                                    *       * **

Prim.cons.        TAACAACTTTCCACAACAC2CA2AAAAAACAACCATGA2CATATTCGGTTTATTAGACCC
```

A IVS in *C. diphtheriae*

```
                     1870      1880      1890      1900      1910      1920
                        |         |         |         |         |         |
T_denticola     AAAGAAAACTAAAAATACAAAACTTCTTTTTAAGCGAAAAGTGTAAAGAAAGAAGGGAC
T_denticola     AAAGAAAACTAAAAATACAAAACTTCTTTTTAAGCGAAAAGTGTAAAGAAAGAAGGGAC
T_pallidum      AGGGTGGGGTGTGAA---------------------------GTTGAGAAGGGATGG--
T_pallidum      AGGGTGGGGTGTGAA---------------------------GTTGAGAAGGGATGG--
                *  *     *   **                           **  ****  * *  **

Prim.cons.      A22G22222T222AATACAAAACTTCTTTTTAAGCGAAAAG2GT22AGAA2G2A2GGAC
```

```
                     4990      5000      5010      5020      5030      5040
                        |         |         |         |         |         |
T_denticola     ATTTTAAGTTTTAAGTCCTTAATTTGTTTATTATTGCCAGGTGGCCATAGTGGAGAGGTA
T_denticola     AAATAAAGTTTTAAGTCCTTAATTTGTTTATTATTGCCAGGTGGCCATAGTGGAGAGGTA
T_pallidum      ----------------------------TATT-TCGCCTGGTTGCCATGGTGGAGAGGTC
T_pallidum      ----------------------------TATT-TCGCCTGGTTGCCATGGTGGAGAGGTC
                                            **** * *** *** ***** **********

Prim.cons.      A22T2AAGTTTTAAGTCCTTAATTTGTTTATTAT2GCC2GGT2GCCAT2GTGGAGAGGT2
```

B IVS in *T. denticola*

Figure S5 (C)

```
                                1630      1640      1650      1660      1670      1680
                                   |         |         |         |         |         |
Y_pestis                    GAACCTGCGGTTGGATCAC-CTCCTTACCTAACGATACGCATTGCGCAGTGCCCACACAG
Y_pestis                    GAACCTGCGGTTGGATCAC-CTCCTTACCTAACGATACGCATTGCGCAGTGCCCACACAG
Y_pestis                    GAACCTGCGGTTGGATCAC-CTCCTTACCTAACGATACGCATTGCGCAGTGCCCACACAG
Y_pestis                    GAACCTGCGGTTGGATCAC-CTCCTTACCTAACGATACGCATTGCGCAGTGCCCACACAG
Y_pestis                    GAACCTGCGGTTGGATCAC-CTCCTTACCTAACGATACGCATTGCGCAGTGCCCACACAG
Y_pseudotuberculosis        GAACCTGCGGTTGGATCAC-CTCCTTACCT------------------------------
Y_pseudotuberculosis        GAACCTGCGGTTGGATCAC-CTCCTTACCT------------------------------
Y_pseudotuberculosis        GAACCTGCGGTTGGATCAC-CTCCTTACCT------------------------------
Y_pseudotuberculosis        GAACCTGCGGTTGGATCAC-CTCCTTACCT------------------------------
Y_pseudotuberculosis        GAACCTGCGGTTGGATCAC-CTCCTTACCT------------------------------
Y_pestis                    CAATCTGAGCCATGATCAAACTCTTCAATT------------------------------
Y_pestis                    CAATCTGAGCCATGATCAAACTCTTCAATT------------------------------
Y_pseudotuberculosis        CAATCTGAGCCATGATCAAACTCTTCAATTT-----------------------------
Y_pseudotuberculosis        CATTCTGAGCCATGATCAAACTCTTCAATTT-----------------------------
                             *  *** *    *****  *** * *  *
Prim.cons.                  GAACCTGCGGTTGGATCACACTCCTTACCTAACGATACGCATTGCGCAGTGCCCACACAG

                                1690
                                   |
Y_pestis                    ATTGTCTGAT
Y_pestis                    ATTGTCTGAT
Y_pestis                    ATTGTCTGAT
Y_pestis                    ATTGTCTGAT
Y_pestis                    ATTGTCTGAT
Y_pseudotuberculosis        ----------
Y_pseudotuberculosis        ----------
Y_pseudotuberculosis        ----------
Y_pseudotuberculosis        ----------
Y_pseudotuberculosis        ----------
Y_pestis                    ----------
Y_pestis                    ----------
Y_pseudotuberculosis        ----------
Y_pseudotuberculosis        ----------

Prim.cons.                  ATTGTCTGAT
```

C. IVS in *Y. pestis*

Figure S5 (D)

```
                        5230      5240      5250      5260      5270      5280
                           |         |         |         |         |         |
S_pyogenes        TGTAGTTGGGGTTGCCCCCTGTGAGATAAGGTAGTCGCTTAGC----------------
S_pyogenes        TGTAGTTGGGGTTGCCCCCTGTGAGATAAGGTAGTCGCTTAGC----------------
S_pyogenes        TGTAGTTGGGGTTGCCCCCTGTGAGATAAGGTAGTCGCTTAGC----------------
S_suis            TGTAGTTGGGGTTGCCCCCTGTGAGATAAGGTAGTCGCTTAGC----------------
S_suis            AGTAGTTGGGGTTGCCCCCTGTTAGATACGGTAGTCGCTTAGCA---------------
S_suis_05ZYH33    AGTAGTTGGGGTTGCCCCCTGTTAGATACGGTAGTCGCTTAGCA---------------
S_suis_05ZYH33    AGTAGTTGGGGTTGCCCCCTGTTAGATACGGTAGTCGCTTAGCA---------------
S_suis_05ZYH33    AGTAGTTGGGGTTGCCCCCTGTTAGATACGGTAGTCGCTTAGCA
S_pyogenes        -GTTCTCAGCGTTCTACTTGCATGTATTAGGCACGCCGCC-AGCGTTCGTCCTGAGCCAG
S_pyogenes        -GTTCTCAGCGTTCTACTTGCATGTATTAGGCACGCCGCC-AGCGTTCGTCCTGAGCCAG
                   **  *  * * *   *     *    ** *   * ***  ***

Prim.cons.        2GTAGTTGGGGTTGCCCCCTGTGAGATA2GGTAGTCGCTTAGCATTCGTCCTGAGCCAG

                       5290
                          |
S_pyogenes        -----------------
S_pyogenes        -----------------
S_pyogenes        -----------------
S_suis            -----------------
S_suis1           -----------------
S_suis_05ZYH33    -----------------
S_suis_05ZYH33    -----------------
S_suis_05ZYH33
S_pyogenes        GATCAAACTCTCTTTAA
S_pyogenes        GATCAAACTCTCTTTAA

Prim.cons.        GATCAAACTCTCTTTAA
```

D IVS in *S. pyogenes*

Figure S5 (E ; F...)

```
                   5350      5360      5370      5380      5390      5400
                      |         |         |         |         |         |
S_Typhi            GGCCCGAAGGTCCCCCTCT-----------TTGGTCTTGCGACGTTATGCGGTATTAGCC
S_Typhi            GGCCCGAAGGTCCCCCTCT-----------TTGGTCTTGCGACGTTATGCGGTATTAGCC
S_Typhi            GGCCCGAAGGTCCCCCTCT-----------TTGGTCTTGCGACGTTATGCGGTATTAGCC
S_Typhi            GGCCCGAAGGTCCCCCTCT-----------TTGGTCTTGCGACGTTATGCGGTATTAGCC
S_Typhi            GGCCCGAAGGTCCCCCTCT-----------TTGGTCTTGCGACGTTATGCGGTATTAGCC
S_Typhi            GGAACGAAAG--------------------------------------------------
S_Typhi            GGAACGAAAG--------------------------------------------------
S_Schwarzengrund   GGAACGAAAG--------------------------------------------------
S_Schwarzengrund   GGAAAGGAAGGAGCATACAGAAGTATGTGACTGACTTTACGAGCGCAGGCAACGCCGCTG
S_Schwarzengrund   GAAAAGGAAGGAGCATACTGAAGTATGTGACTGACTTTGCGAATGCAGCCAACGCAGC-A
                   *    * * *
Prim.cons.         GG22CGAA2GTCCCCCTCTGAAGTATGTGATTGGTCTTGCGACGTTATGCGGTATTAGCC
```

E IVS in *S. Typhi*

```
                   1690      1700      1710      1720      1730      1740
                      |         |         |         |         |         |
S_Typhi            GTTAAAGTGAACCGGATTTACCTG----GAACACATACCTAC---------------ACG
S_Typhi            GTTAAAGTGAACCGGATTTACCTG----GAACACATACCTAC---------------ACG
S_Typhi            GTTAAAGTGAACCGGATTTACCTG----GAACACATACCTAC---------------ACG
S_Typhi            GTTAAAGTGAACCGGATTTACCTG----GAACACATACCTAC---------------ACG
S_Typhi            GTTAAAGTGAACCGGATTTACCTG----GAACACATACCTAC---------------ACG
S_Typhi            GTCT-TGCGAAGCAGACTGATACG--T--------CCCCT-----------------TCG
S_Typhi            GTCT-TGCGAAGCAGACTGATACG--T--------CCCCT-----------------TCG
S_Schwarzengrund   CTCTACAGGCTTGTAGCTCAGGTGGTTAGAGCGCACCCCTGATAAGGGTGAGGTCGGTGG
S_Schwarzengrund   CTCTACAGGCTTGTAGCTCAGGTGGTTAGAGCGCACCCCTGATAAGGGTGAGGTCGGTGG
S_Schwarzengrund   GTCT-TGCGATTGAGACTTCAGTG-T---------CCCCT-----------------TCG
                    *      *        *     *             ***                   *
Prim.cons.         GT22AAGTGAACCGGA2TTACCTGGTTAGAACACA22CCTACTAAGGGTGAGGTCGG2CG
```

IVS in *S. Schwarzengrund*

Figure S5 (F)

```
                              1750      1760      1770      1780      1790      1800
                                 |         |         |         |         |         |
S_Typhi             CTTAAACC-----GGGACAACC-----------------------GTCGCCCGGCCA--A
S_Typhi             CTTAAACC-----GGGACAACC-----------------------GTCGCCCGGCCA--A
S_Typhi             CTTAAACC-----GGGACAACC-----------------------GTCGCCCGGCCA--A
S_Typhi             CTTAAACC-----GGGACAACC-----------------------GTCGCCCGGCCA--A
S_Typhi             CTTAAACC-----GGGACAACC-----------------------GTCGCCCGGCCA--A
S_Typhi             TCTAG--------AGGCCCA--------------------------------GG-----A
S_Typhi             TCTAG--------AGGCCCA--------------------------------GG-----A
S_Schwarzengrund    TTCAAGTCCACTCAGGCCTACCAAATTTTCCCTGAATACTGCGTTGTGAAATAACTCACA
S_Schwarzengrund    TTCAAGTCCACTCAGGCCTACCAGATTCGCTCCCG-TGCTTTGTTGTGGCAAAGCTCGCA
S_Schwarzengrund    TCTAG--------AGGCCCA-------------------------------G-G-----A
                       *          ** * *                                       *

                              1810      1820      1830      1840      1850      1860
                                 |         |         |         |         |         |
S_Typhi             CATAGCCTTCTCCGTCCCCCCTTCGCA--GTAACACCAAGTA------CGGGAATATTAA
S_Typhi             CATAGCCTTCTCCGTCCCCCCTTCGCA--GTAACACCAAGTA------CGGGAATATTAA
S_Typhi             CATAGCCTTCTCCGTCCCCCCTTCGCA--GTAACACCAAGTA------CGGGAATATTAA
S_Typhi             CATAGCCTTCTCCGTCCCCCCTTCGCA--GTAACACCAAGTA------CGGGAATATTAA
S_Typhi             CATAGCCTTCTCCGTCCCCCCTTCGCA--GTAACACCAAGTA------CGGGAATATTAA
S_Typhi             CAC------------CGCCCTTTCACG--GC------------------GGTAACAGGGG
S_Typhi             CAC------------CGCCCTTTCACG--GC------------------GGTAACAGGGG
S_Schwarzengrund    TACTGATGTATGCTTCGTTATTCCACGCCTTGTCTCAGGAAAAATTATCGGTAAAGAGGT
S_Schwarzengrund    TACTTCAGTATGCTTCGCTTCACCACGCCGCGCCCGGAAACGAATCAAAGGTAA-GAGGT
S_Schwarzengrund    CAC---------C---GCCCTTTCACG--GC------------------GGTAACAGGGG
                     *                     * *                       **  **

                              1870      1880      1890      1900      1910      1920
                                 |         |         |         |         |         |
S_Typhi             CCCGTTTCCCATCGACTAC----------------GCCTTTCGGCCTC----------GC
S_Typhi             CCCGTTTCCCATCGACTAC----------------GCCTTTCGGCCTC----------GC
S_Typhi             CCCGTTTCCCATCGATTAC----------------GCCTTTCGGCCTC----------GC
S_Typhi             CCCGTTTCCCATCGACTAC----------------GCCTTTCGGCCTC----------GC
S_Typhi             CCCGTTTCCCATCGACTAC----------------GCCTTTCGGCCTC----------GC
S_Typhi             TTCGAATCCCCTAGGGGAC----------------GC-CACTTGCG-------C-----G
S_Typhi             TTCGAATCCCCTAGGGGAC----------------GC-CACTTGCG-------C-----G
S_Schwarzengrund    TCTGACTACACGATGGGGCTATAGCTCAGCTGGGAGAGCGCCTGCTTTGCACGCAGGAGG
S_Schwarzengrund    TCTGACTACACGATGGGGCTATAGCTCAGCTGGGAGAGCGCCTGCTTTGCACGCAGGAGG
S_Schwarzengrund    TTCGAATCCCCTAGGGGAC----------------GC-CACTTGCT------------GG
```

IVS in *S. Schwarzengrund*

Figure S5 (F ; G)

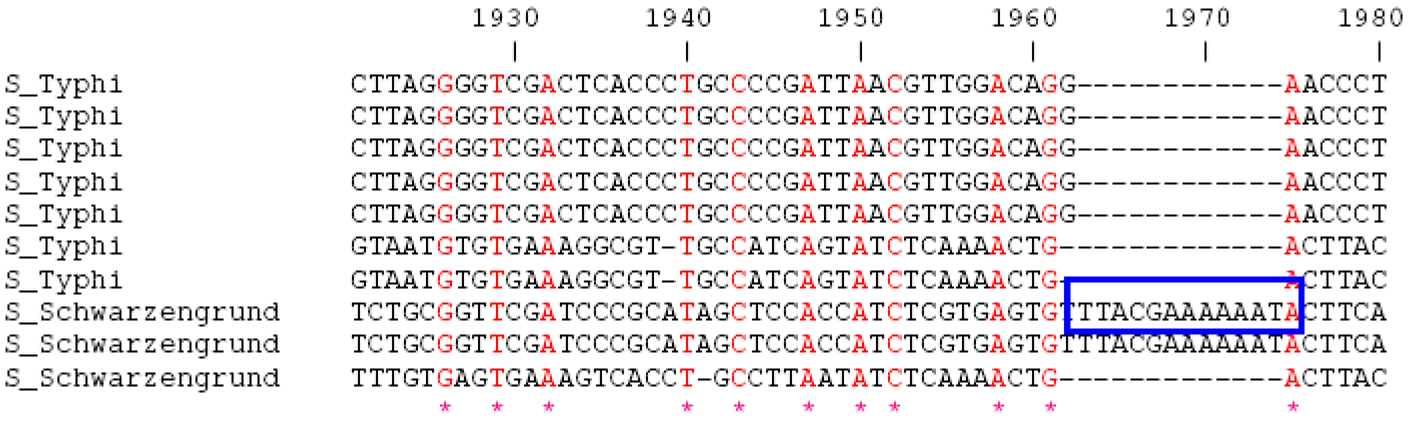

F IVS in *S. Schwarzengrund*

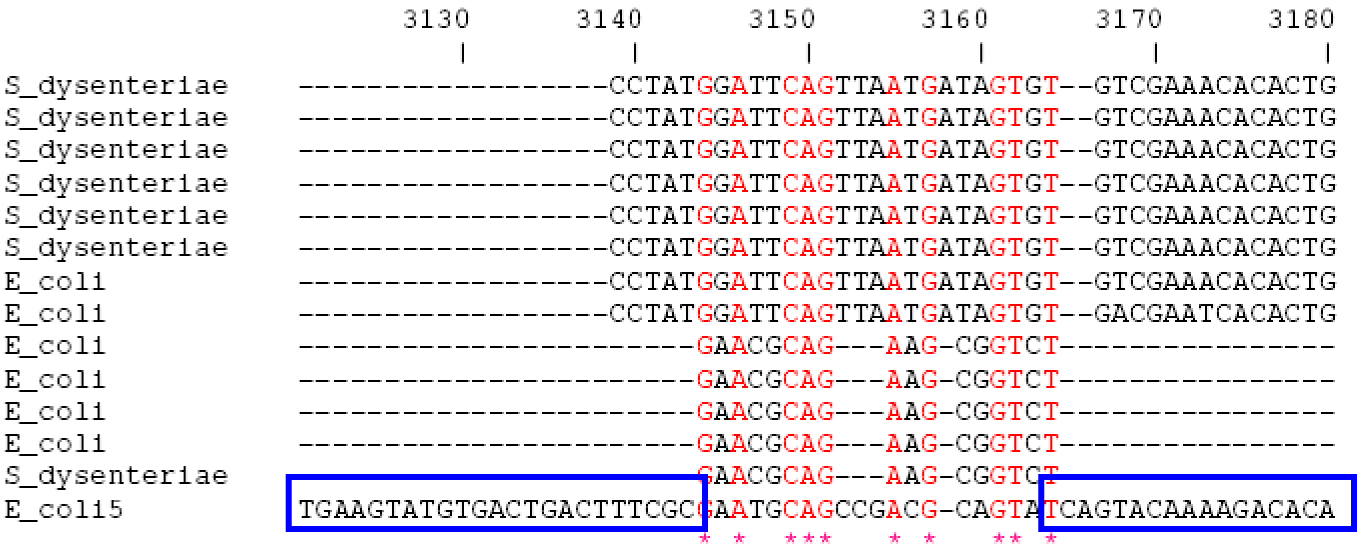

G IVS in *E. coli HS*

Figure S5 (H)

```
                                1570      1580      1590      1600      1610      1620
                                   |         |         |         |         |         |
V_parahaemolyticus        GCGTTCAATCTGAGCCATGATCAAACTCTTCAAT--------------------------
V_parahaemolyticus        GCGTTCAATCTGAGCCATGATCAAACTCTTCAAT--------------------------
V_parahaemolyticus        GCGTTCAATCTGAGCCATGATCAAACTCTTCAAT--------------------------
V_parahaemolyticus        GCGTTCAATCTGAGCCATGATCAAACTCTTCAAT--------------------------
V_parahaemolyticus        GCGTTCAATCTGAGCCATGATCAAACTCTTCAAT--------------------------
V_parahaemolyticus        GCGTTCAATCTGAGCCATGATCAAACTCTTCAAT--------------------------
V_parahaemolyticus        GCGTTCAATCTGAGCCATGATCAAACTCTTCAAT--------------------------
V_parahaemolyticus        GCGTTCAATCTGAGCCATGATCAAACTCTTCAAT--------------------------
V_cholerae                GCGTTCAATCTGAGCCATGATCAAACTCT-------------------------------
V_cholerae                GCGTTCAATCTGAGCCATGATCAAACTCT-------------------------------
V_cholerae                GCGTTCAATCTGAGCCATGATCAAACTCT-------------------------------
V_cholerae                GCGTTCAATCTGAGCCATGATCAAACTCT-------------------------------
V_cholerae                GCAGGTAGTTTAACCTTCGGGAGGACGCTTGCCACTTTGTGGTTCATGACTGGGGTGAAG
V_cholerae                GCAGGTAGTTTAACCTCCGGGAGGACGCTTGCCACTTTGTGGTTCATGACTGGGGTGAAG
V_cholerae                GCAGGTAGTTTAACCTTCGGGAGGACGCTTGCCACTTTGTGGTTCATGACTGGGGTGAAG
V_cholerae                GCAGGTAGTTTAACCTTCGGGAGGACGCTTGCCACTTTGTGGTTCATGACTGGGGTGAAG
V_parahaemolyticus        GTAGGTAGTTTAACCTTCGGGGGGACGC--------------------------------
V_parahaemolyticus        GTAGGTAGTTTAACCTTCGGGGGGGCGC--------------------------------
                          *     * * * * *   *      * *

                                1630      1640      1650      1660
                                   |         |         |         |
V_parahaemolyticus        ------------------------------------------------
V_parahaemolyticus        ------------------------------------------------
V_parahaemolyticus        ------------------------------------------------
V_parahaemolyticus        ------------------------------------------------
V_parahaemolyticus        ------------------------------------------------
V_parahaemolyticus        ------------------------------------------------
V_parahaemolyticus        ------------------------------------------------
V_parahaemolyticus        ------------------------------------------------
V_cholerae                ------------------------------------------------
V_cholerae                ------------------------------------------------
V_cholerae                ------------------------------------------------
V_cholerae                ------------------------------------------------
V_cholerae                TCGTAACAAGGTAGCGCTAGGGGAACCTGGCGCTGGATCACCTCCTTA
V_cholerae                TCGTAACAAGGTAGCGCTAGGGGAACCTGGCGCTGGATCACCTCCTTA
V_cholerae                TCGTAACAAGGTAGCGCTAGGGGAACCTGGCGCTGGATCACCTCCTTA
V_cholerae                TCGTAACAAGGTAGCGCTAGGGGAACCTGGCGCTGGATCACCTCCTTA
V_parahaemolyticus        ------------------------------------------------
```

IVS in *V. cholerae*

```
V_parahaemolyticus2     ---------------------------------------------------------
```

H IVS in *V. cholerae*

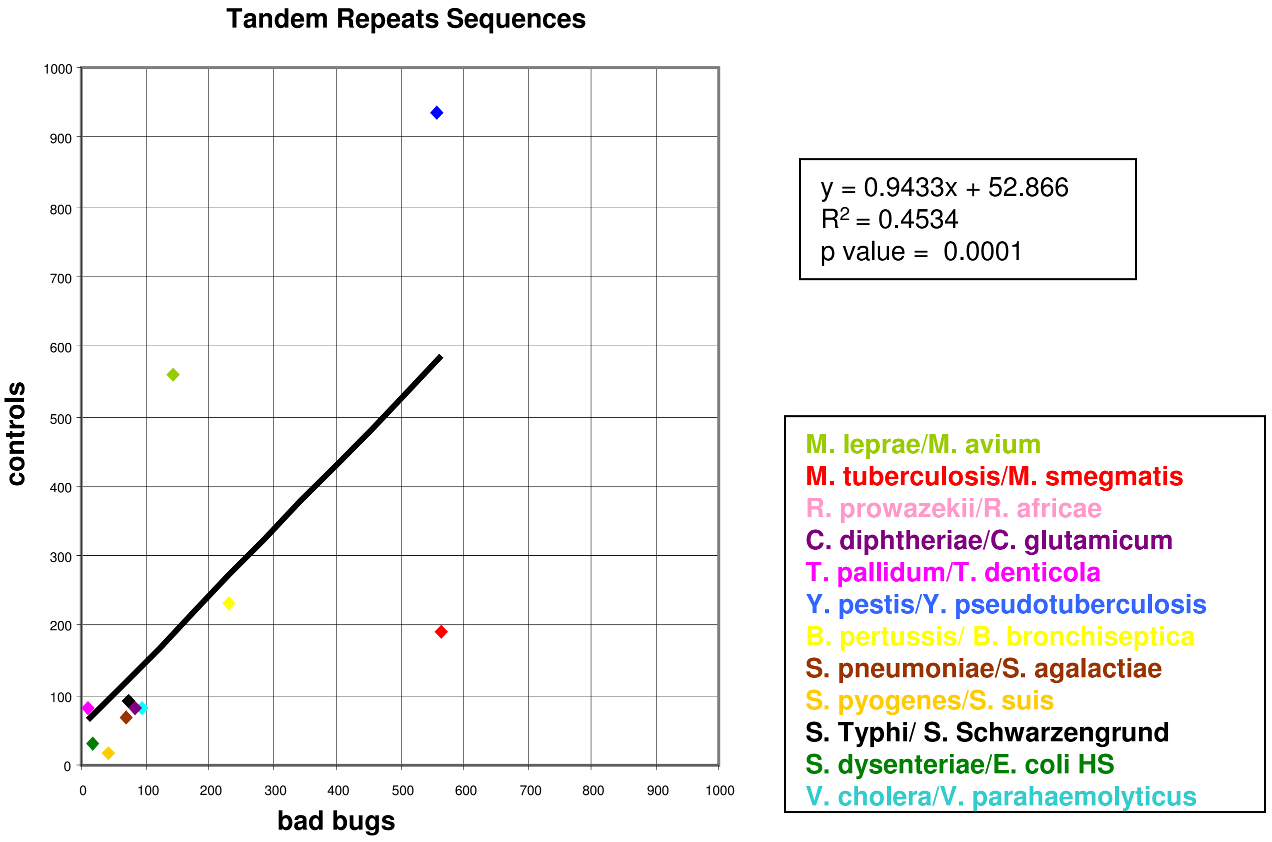

Figure S6:

Graphical representation of the tandem repeat sequence content of “bad bugs” and of controls

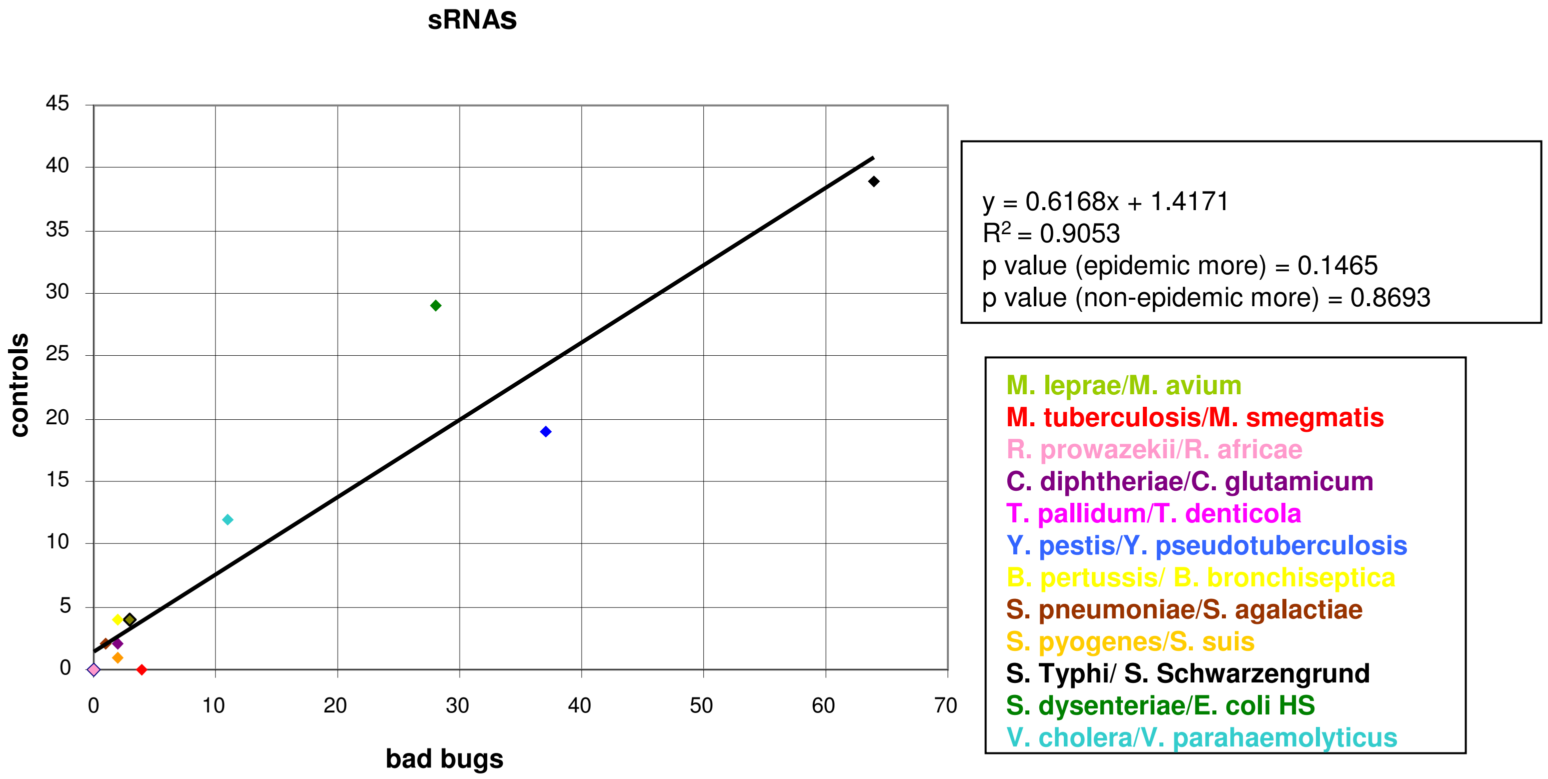

Figure S7:

Graphical representation of the possible sRNA content of “bad bugs” and of controls

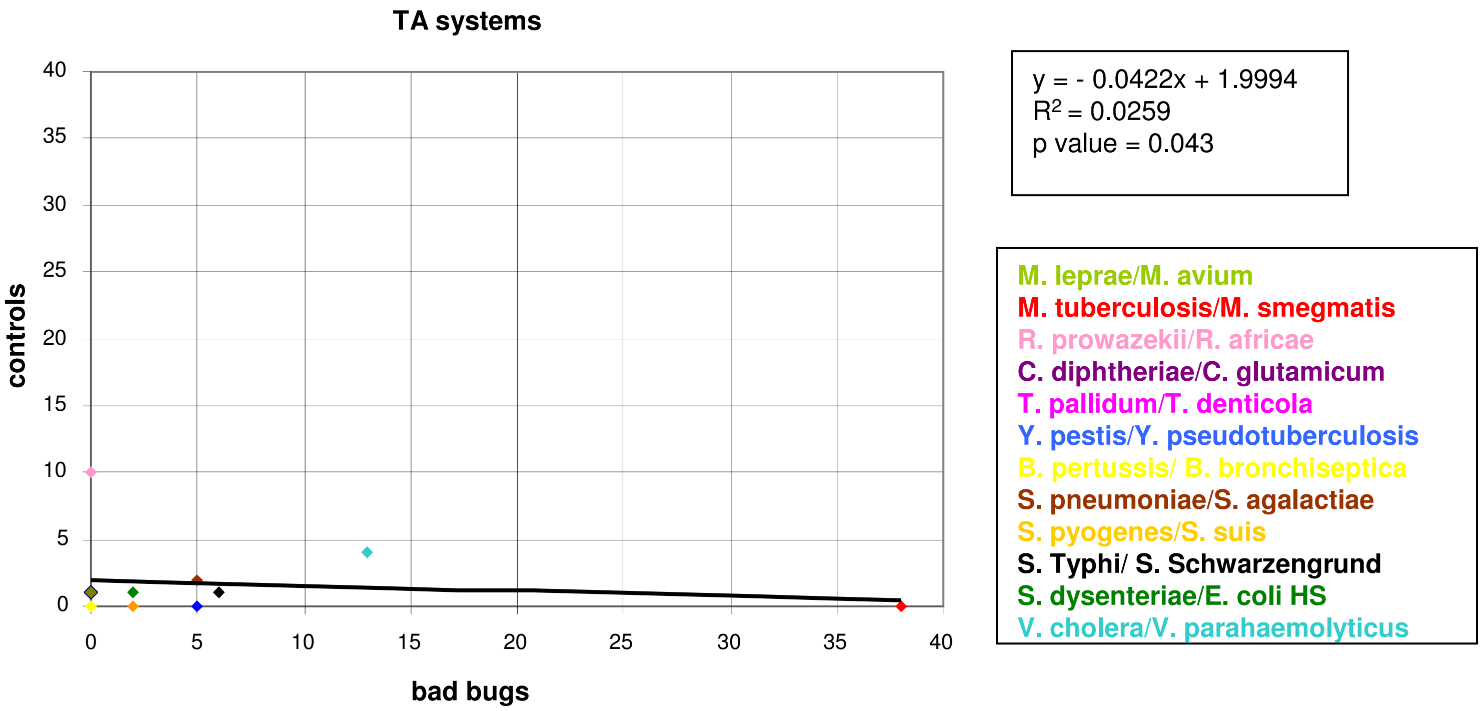

Figure S8:

Graphical representation of the toxin-antitoxin (TA) system content of "bad bugs" and of controls

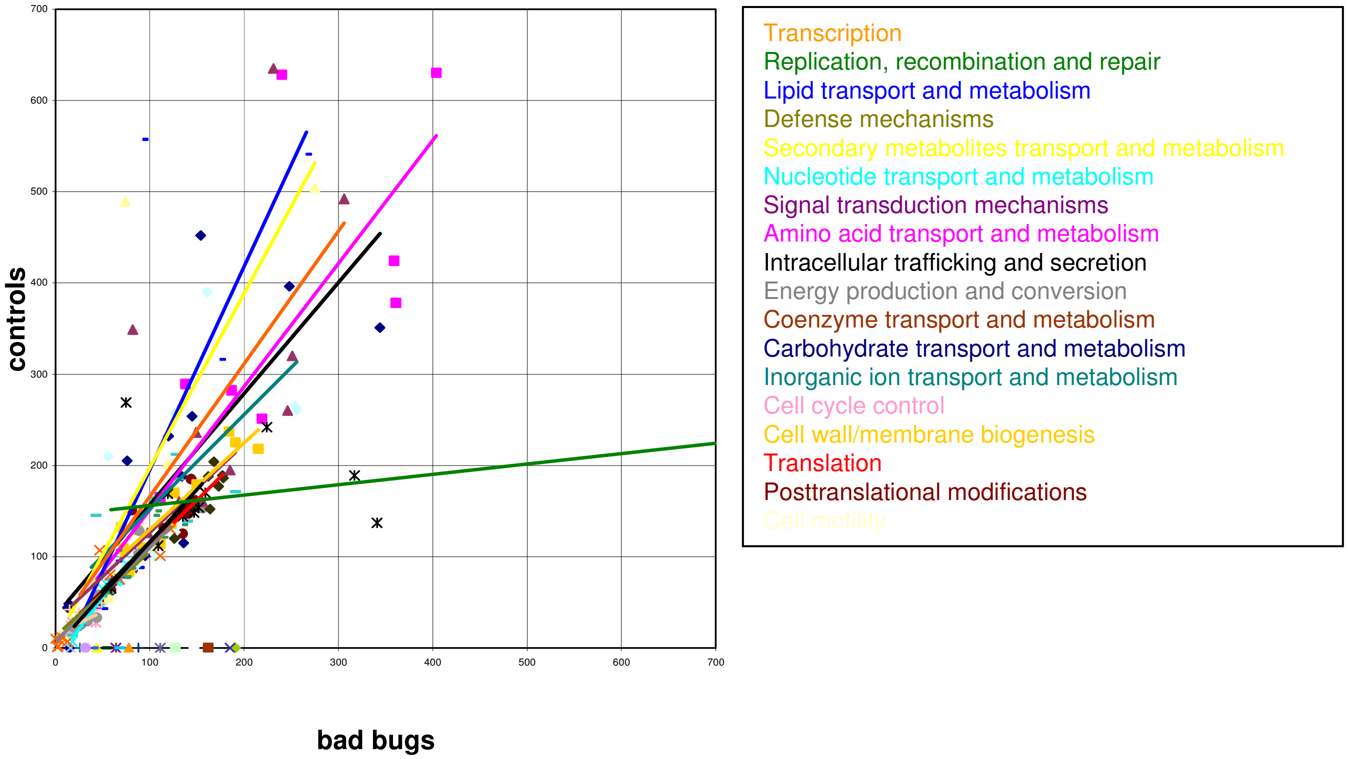

Figure S9:

Graphical representation of the gene content of the 23 COG functional categories

Figure S10:

Graphical representations of the gene content of each of the 18 COG functional categories

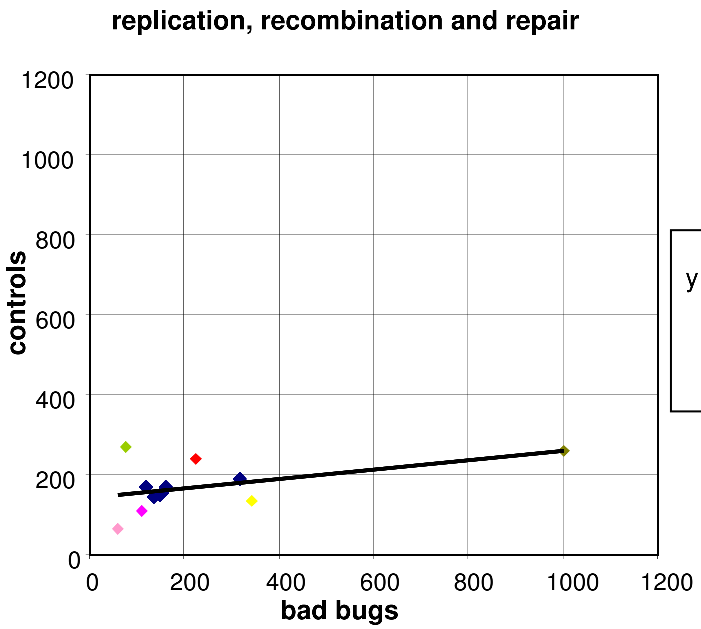

y = 0,1136x + 144,69
R^2 = 0,2319
p value < 0.0001

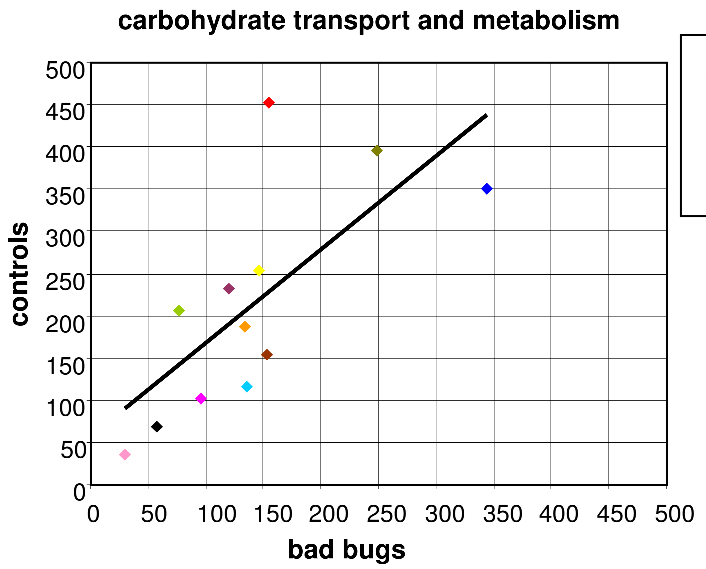

y = 1,1127x + 55,609
R^2 = 0,5127
p value < 0.0001

M. leprae/M. avium
M. tuberculosis/M. smegmatis
R. prowazekii/R. africae
C. diphtheriae/C. glutamicum
T. pallidum/T. denticola
Y. pestis/Y. pseudotuberculosis
B. pertussis/ B. bronchiseptica
S. pneumoniae/S. agalactiae
S. pyogenes/S. suis
S. Typhi/ S. Schwarzengrund
S. dysenteriae/E. coli HS
V. cholera/V. parahaemolyticus

Figure S10:

Graphical representations of the gene content of each of the 18 COG functional categories

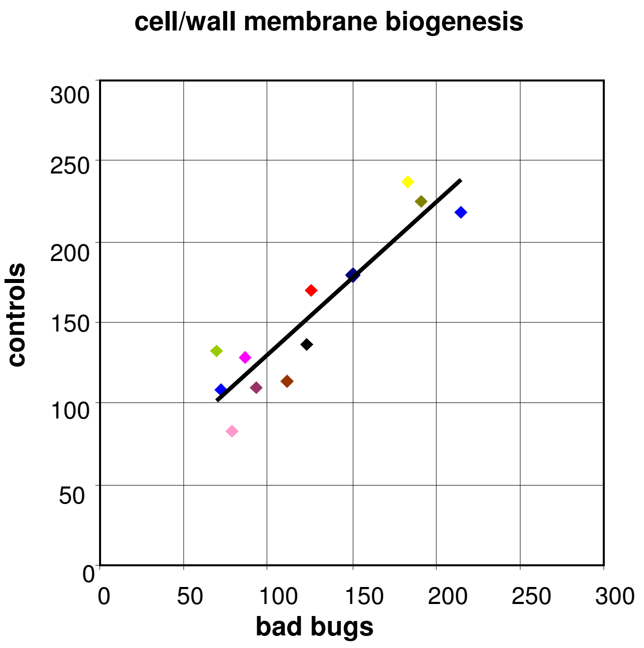

y = 0,9485x +34,935

R2=0,8479

p value = 0.0653

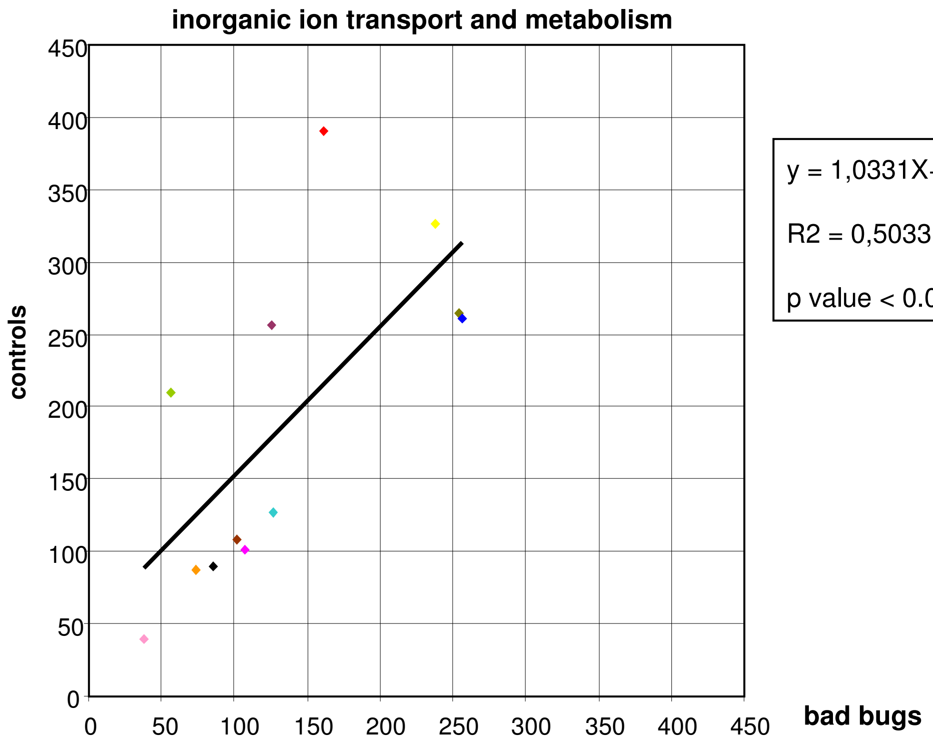

y = 1,0331X+48,942

R2 = 0,5033

p value < 0.0001

M. leprae/M. avium
M. tuberculosis/M. smegmatis
R. prowazekii/R. africae
C. diphtheriae/C. glutamicum
T. pallidum/T. denticola
Y. pestis/Y. pseudotuberculosis
B. pertussis/ B. bronchiseptica
S. pneumoniae/S. agalactiae
S. pyogenes/S. suis
S. Typhi/ S. Schwarzengrund
S. dysenteriae/E. coli HS
V. cholera/V. parahaemolyticus

Figure S10:

Graphical representations of the gene content of each of the 18 COG functional categories

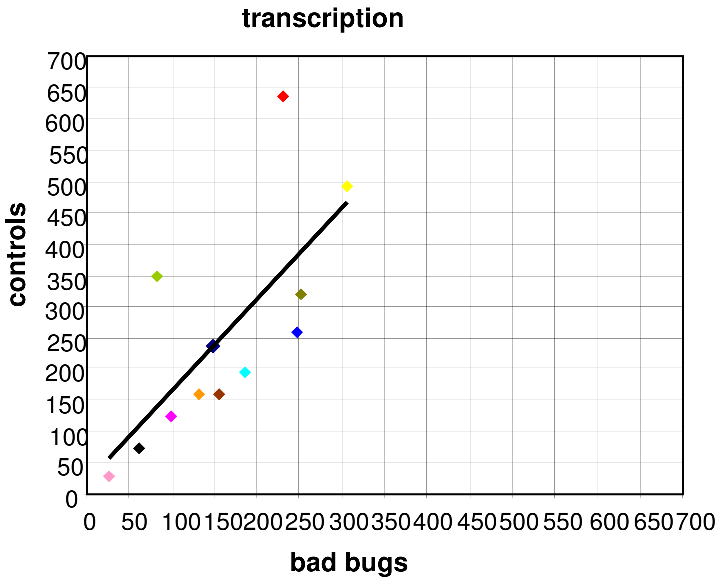

y = 1,4559x + 20,013
$R^2 = 0,5148$
p value < 0.0001

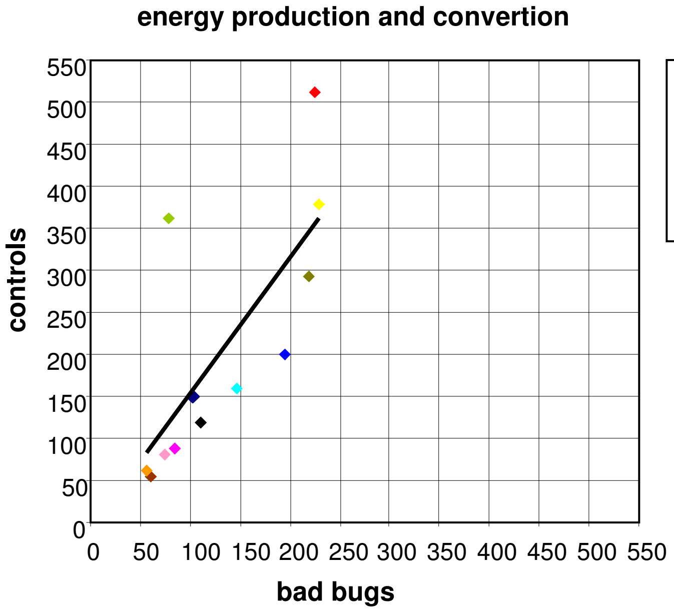

y = 1,6213x - 9,5621
$R^2 = 0,5453$
p value < 0.0001

M. leprae/M. avium
M. tuberculosis/M. smegmatis
R. prowazekii/R. africae
C. diphtheriae/C. glutamicum
T. pallidum/T. denticola
Y. pestis/Y. pseudotuberculosis
B. pertussis/ B. bronchiseptica
S. pneumoniae/S. agalactiae
S. pyogenes/S. suis
S. Typhi/ S. Schwarzengrund
S. dysenteriae/E. coli HS
V. cholera/V. parahaemolyticus

Figure S10:

Graphical representations of the gene content of each of the 18 COG functional categories

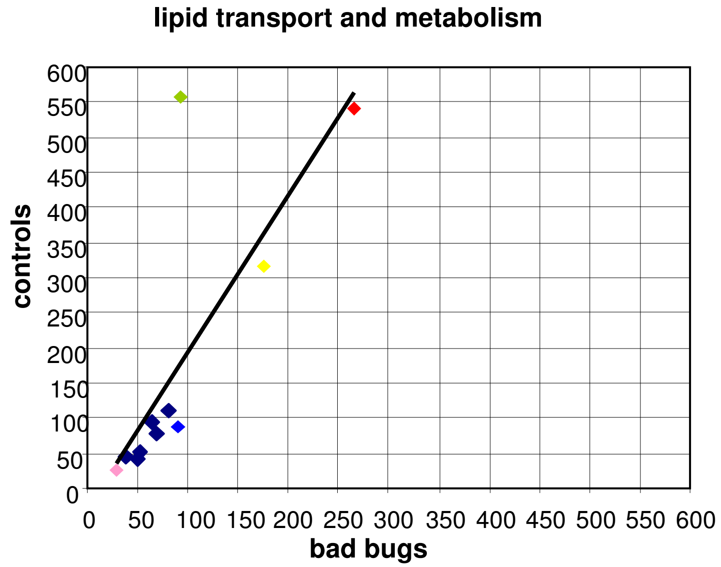

y = 2,2308x - 28,014

$R^2 = 0,6149$

p value < 0.0001

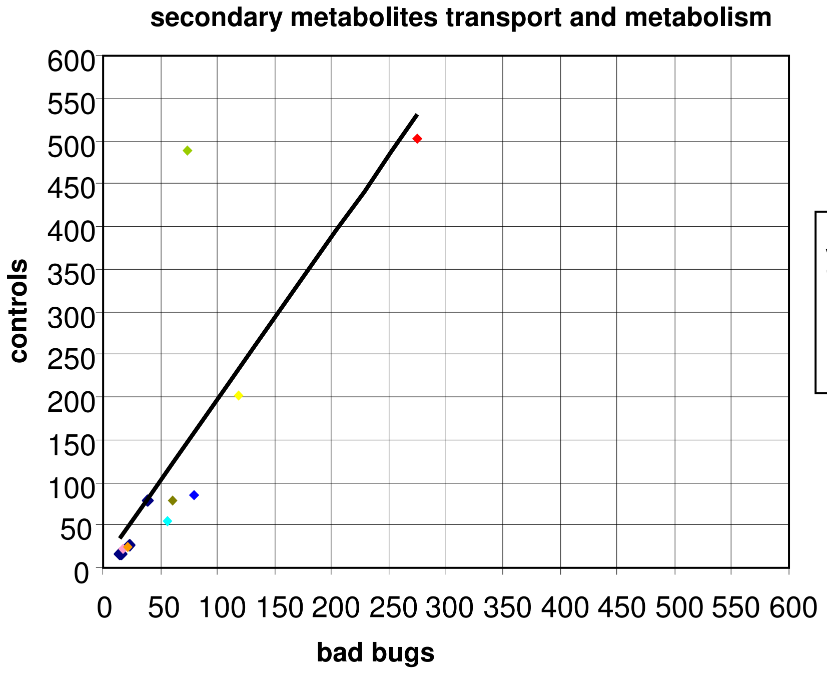

y = 1,9073x + 6,5753

$R^2 = 0,6202$

p value < 0.0001

M. leprae/M. avium
M. tuberculosis/M. smegmatis
R. prowazekii/R. africae
C. diphtheriae/C. glutamicum
T. pallidum/T. denticola
Y. pestis/Y. pseudotuberculosis
B. pertussis/ B. bronchiseptica
S. pneumoniae/S. agalactiae
S. pyogenes/S. suis
S. Typhi/ S. Schwarzengrund
S. dysenteriae/E. coli HS
V. cholera/V. parahaemolyticus

Figure S10:

Graphical representations of the gene content of each of the 18 COG functional categories

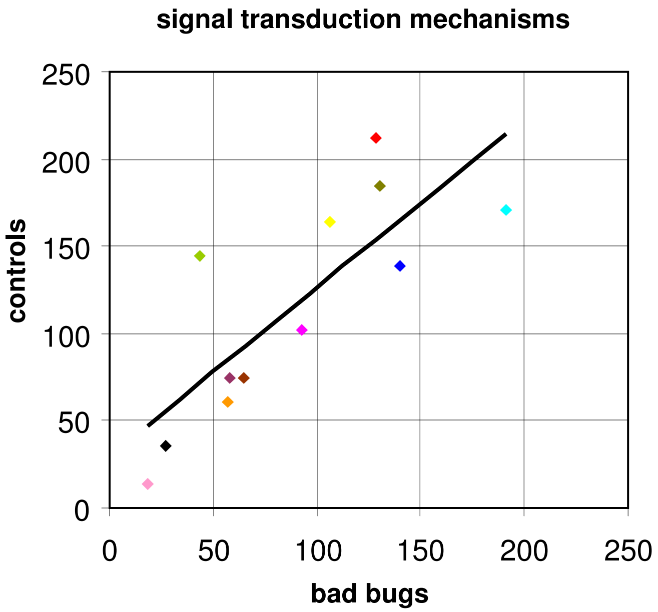

y = 0,967x+29,654

R2= 0,6347

p value < 0.0001

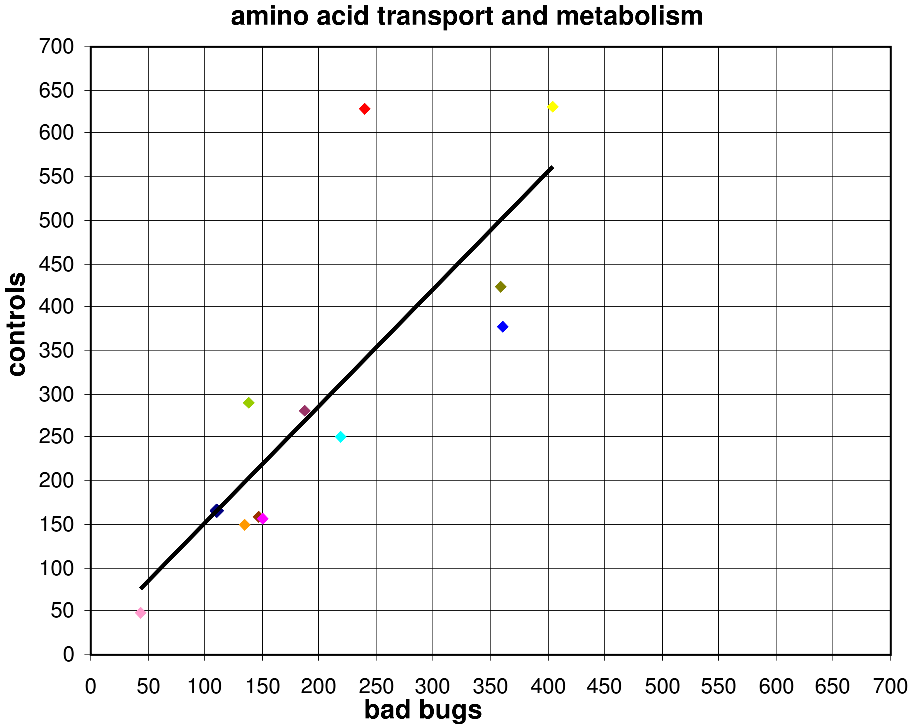

y = 1.3496x + 16.14
R2 = 0.6511
p value < 0.0001

M. leprae/M. avium
M. tuberculosis/M. smegmatis
R. prowazekii/R. africae
C. diphtheriae/C. glutamicum
T. pallidum/T. denticola
Y. pestis/Y. pseudotuberculosis
B. pertussis/ B. bronchiseptica
S. pneumoniae/S. agalactiae
S. pyogenes/S. suis
S. Typhi/ S. Schwarzengrund
S. dysenteriae/E. coli HS
V. cholera/V. parahaemolyticus

Figure S10:

Graphical representations of the gene content of each of the 18 COG functional categories

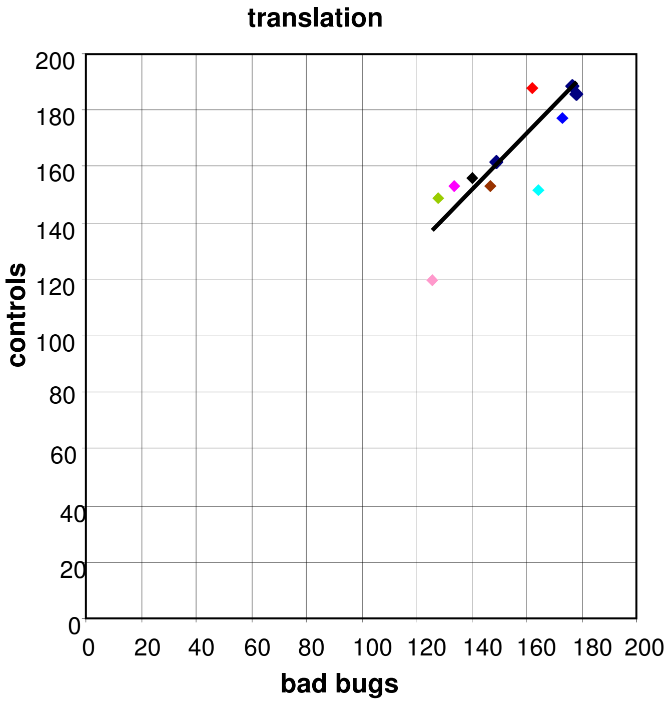

$y = 1{,}0179x + 9{,}162$

$R^2 = 0{,}6794$

p value = 0.8935

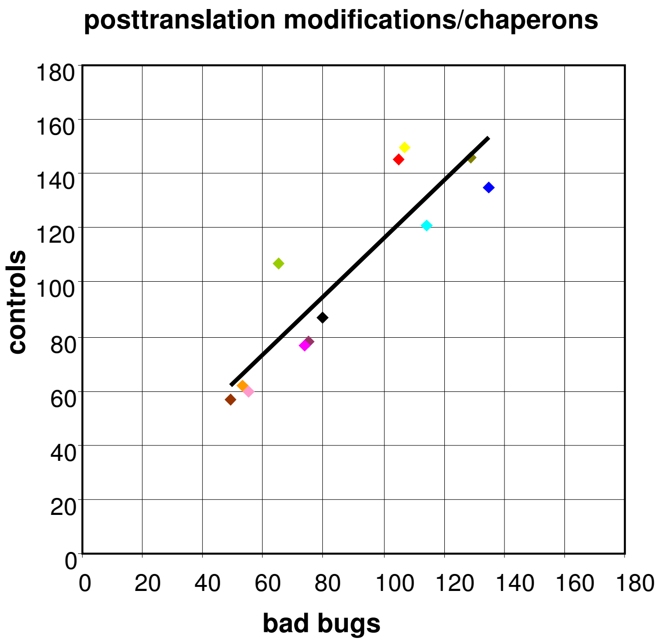

$y = 1{,}0712x + 9{,}1594$

$R^2 = 0{,}7972$

p value = 0.3498

M. leprae/M. avium
M. tuberculosis/M. smegmatis
R. prowazekii/R. africae
C. diphtheriae/C. glutamicum
T. pallidum/T. denticola
Y. pestis/Y. pseudotuberculosis
B. pertussis/ B. bronchiseptica
S. pneumoniae/S. agalactiae
S. pyogenes/S. suis
S. Typhi/ S. Schwarzengrund
S. dysenteriae/E. coli HS
V. cholera/V. parahaemolyticus

Figure S10:

Graphical representations of the gene content of each of the 18 COG functional categories

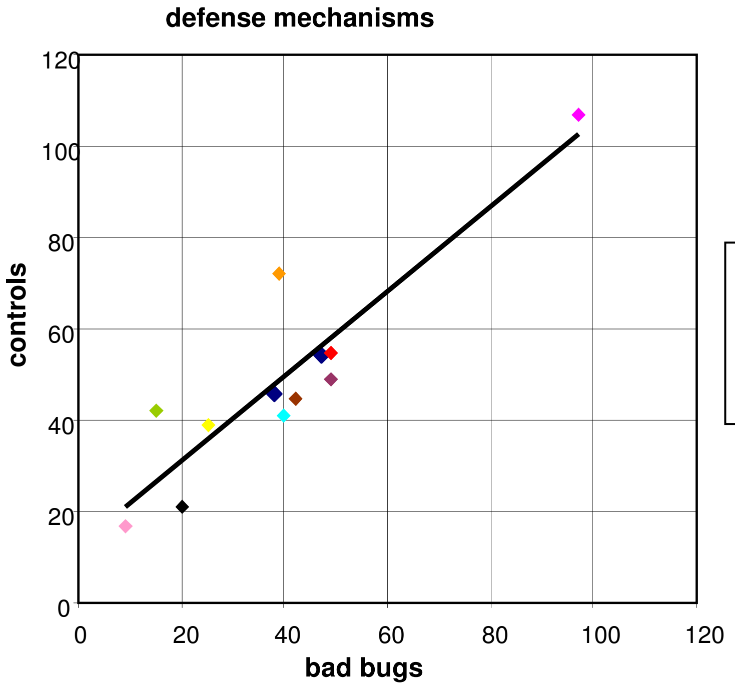

$y = 0{,}9251x + 12{,}766$

$R2 = 0{,}809$

p value = 0.0965

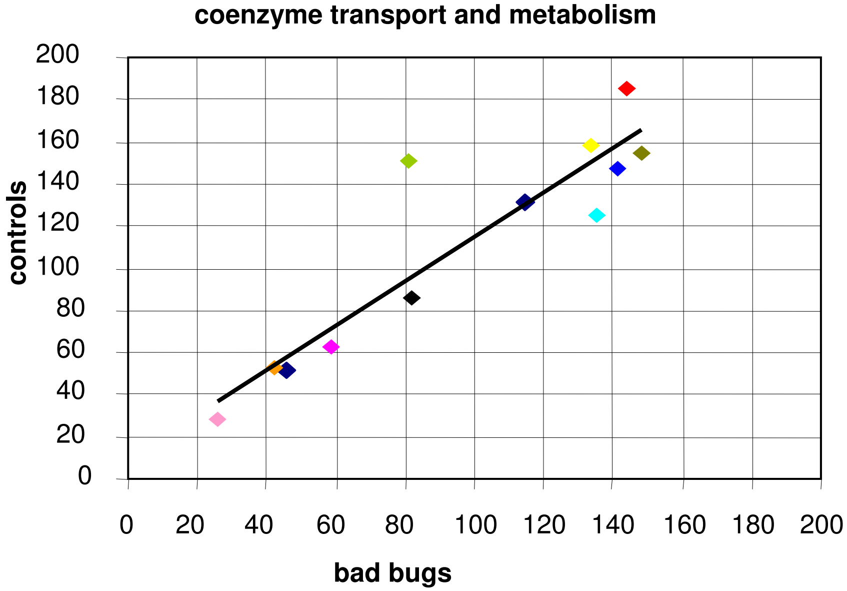

$y = 1{,}05x+10{,}113$

$R2 = 0{,}8309$

p value = 0.0628

M. leprae/M. avium
M. tuberculosis/M. smegmatis
R. prowazekii/R. africae
C. diphtheriae/C. glutamicum
T. pallidum/T. denticola
Y. pestis/Y. pseudotuberculosis
B. pertussis/ B. bronchiseptica
S. pneumoniae/S. agalactiae
S. pyogenes/S. suis
S. Typhi/ S. Schwarzengrund
S. dysenteriae/E. coli HS
V. cholera/V. parahaemolyticus

Figure S10:

Graphical representations of the gene content of each of the 18 COG functional categories

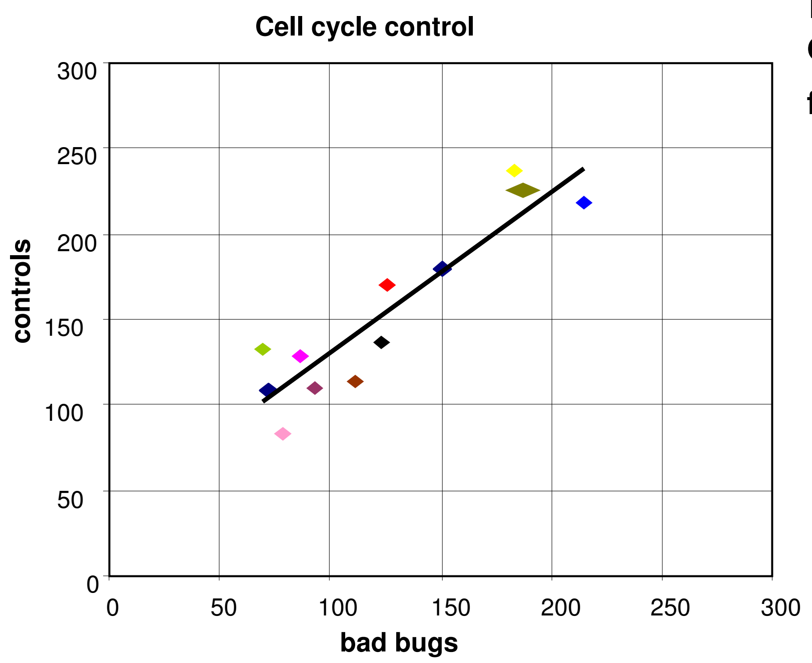

y = 0,9485x + 34,935
R^2 = 0,8479
p value = 0.7932

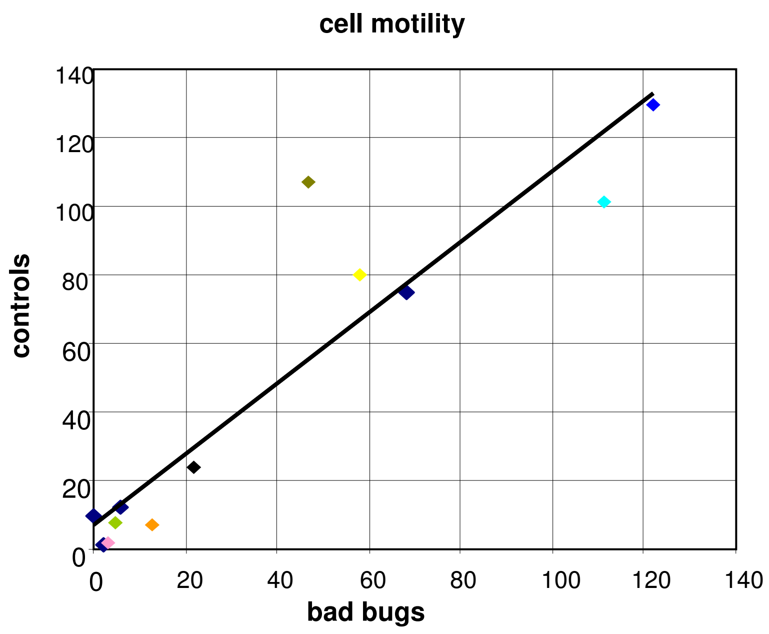

y = 1,031x + 7,1542
R^2 = 0,8585
p value = 0.0004

M. leprae/M. avium
M. tuberculosis/M. smegmatis
R. prowazekii/R. africae
C. diphtheriae/C. glutamicum
T. pallidum/T. denticola
Y. pestis/Y. pseudotuberculosis
B. pertussis/ B. bronchiseptica
S. pneumoniae/S. agalactiae
S. pyogenes/S. suis
S. Typhi/ S. Schwarzengrund
S. dysenteriae/E. coli HS
V. cholera/V. parahaemolyticus

Figure S10:

Graphical representations of the gene content of each of the 18 COG functional categories

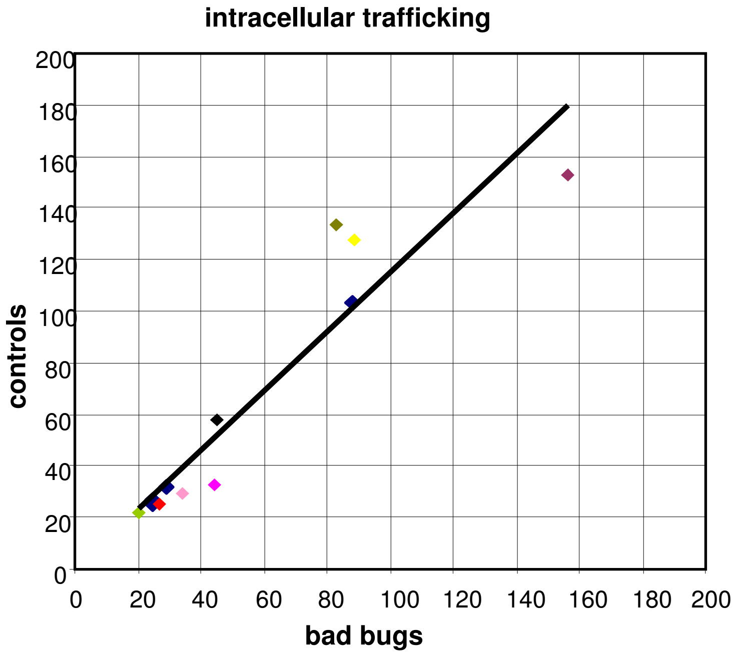

y = 1,1549x + 0,0814

R^2 = 0,8798

p value = 0.1154

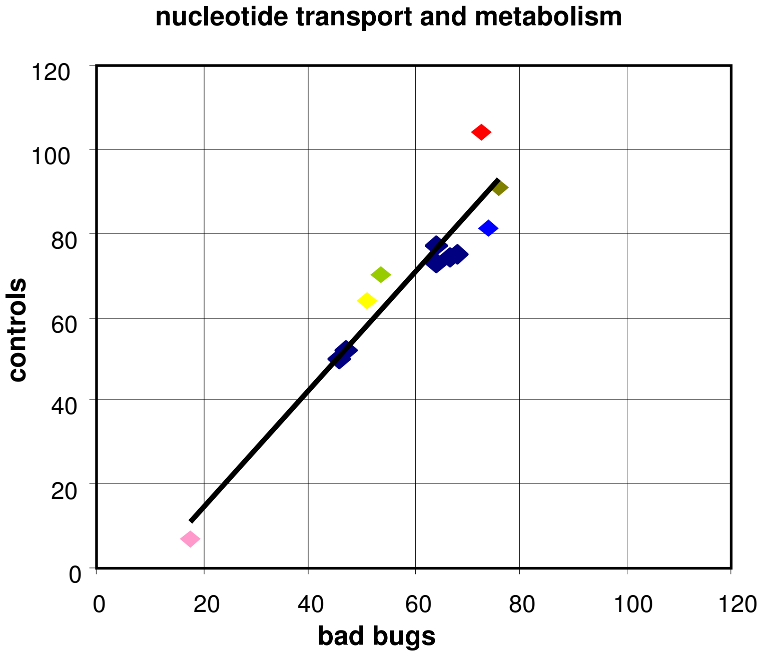

y = 1,4141x-14,556

R2 = 0,9175

p value = 0.5695

M. leprae/M. avium
M. tuberculosis/M. smegmatis
R. prowazekii/R. africae
C. diphtheriae/C. glutamicum
T. pallidum/T. denticola
Y. pestis/Y. pseudotuberculosis
B. pertussis/ B. bronchiseptica
S. pneumoniae/S. agalactiae
S. pyogenes/S. suis
S. Typhi/ S. Schwarzengrund
S. dysenteriae/E. coli HS
V. cholera/V. parahaemolyticus

Figure S11:
Microarray for each functional category

Functional categories for which bad bugs have less genes than their controls

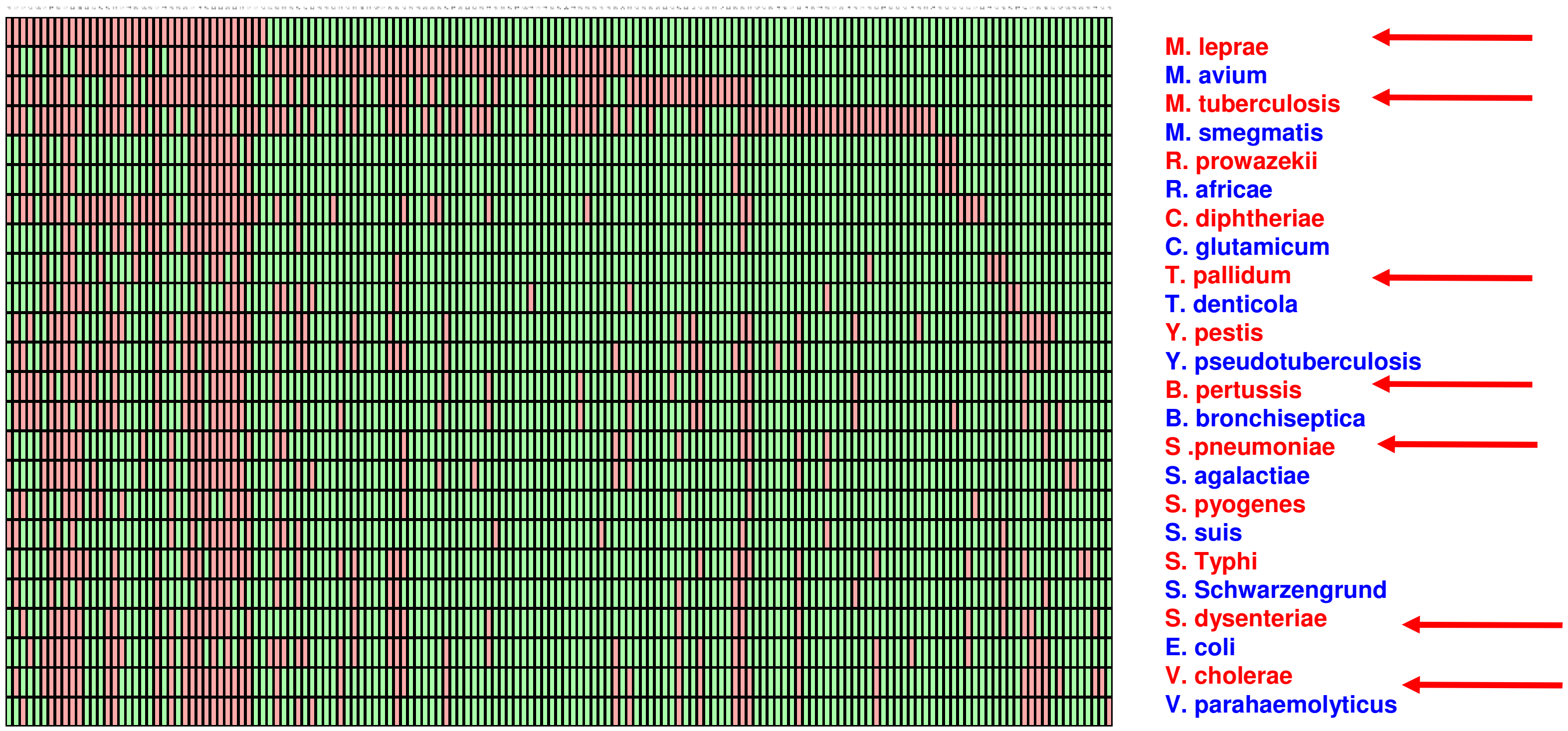

K: Transcription genes

Figure S11:

Microarray for each functional category

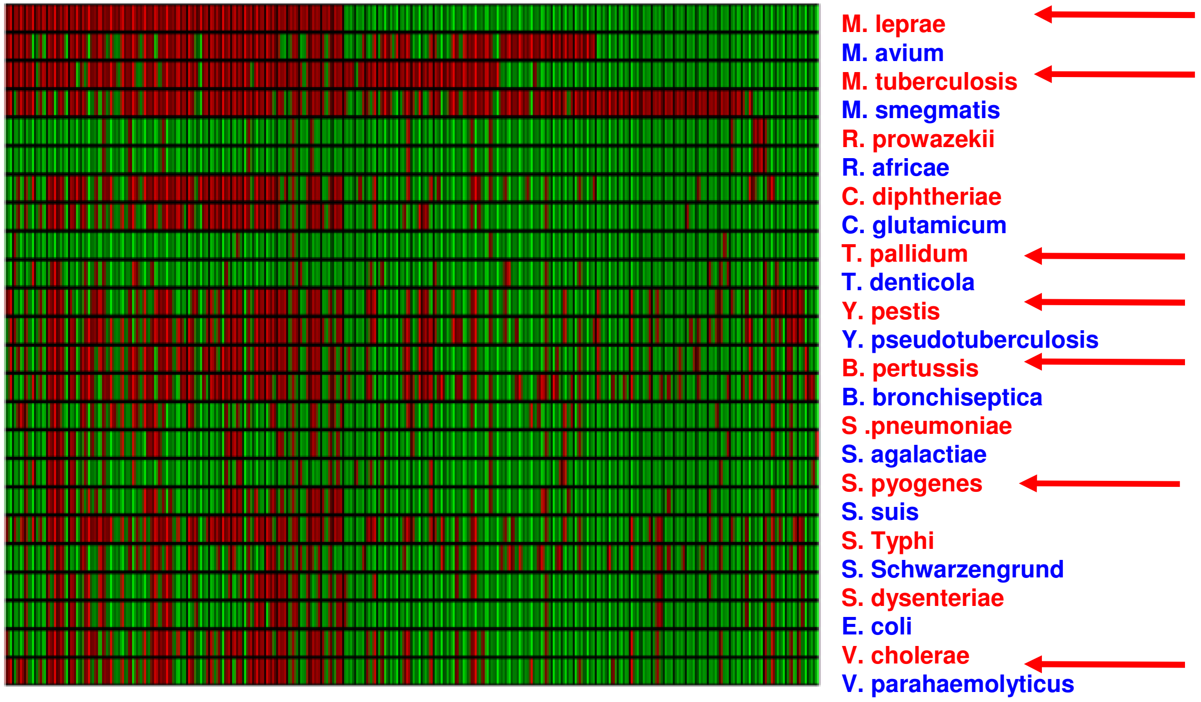

E: Amino acid transport and metabolism

Figure S11:

Microarray for each functional category

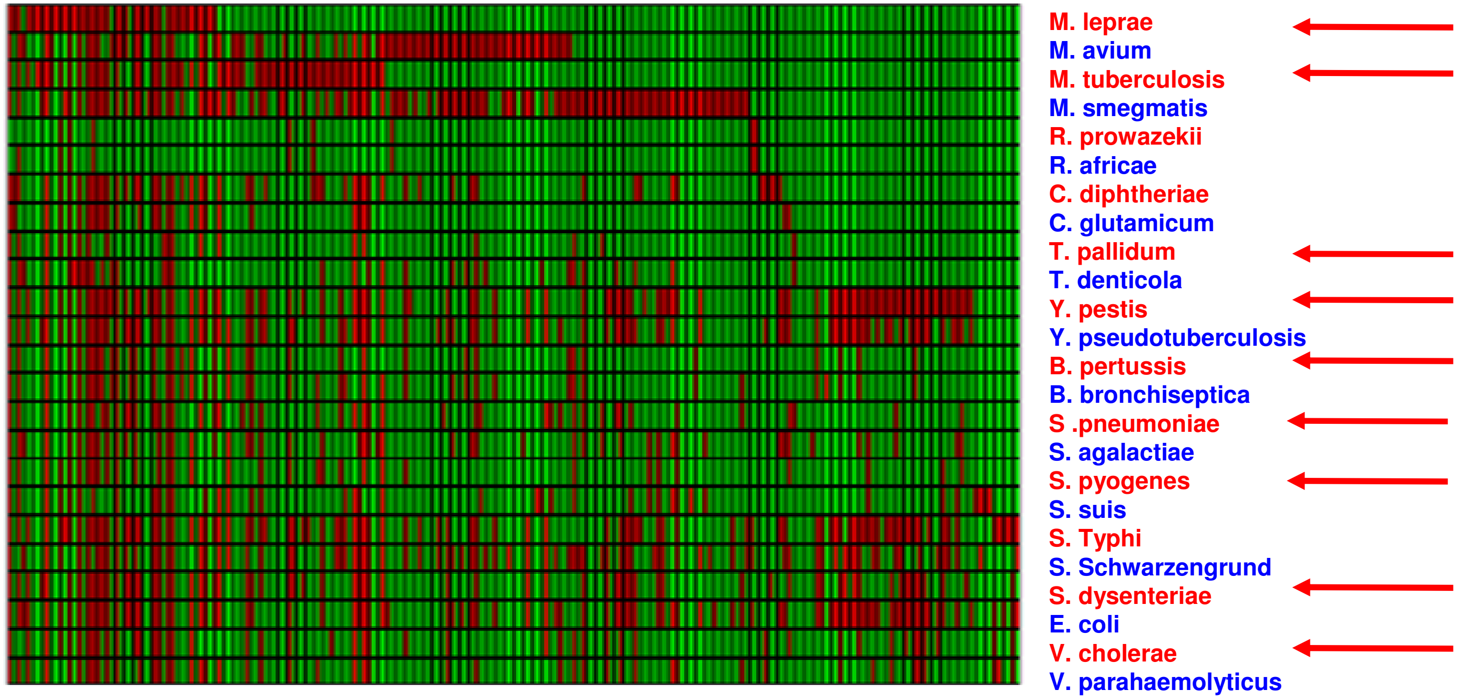

G: Carbohydrate transport and metabolism

Figure S11:

Microarray for each functional category

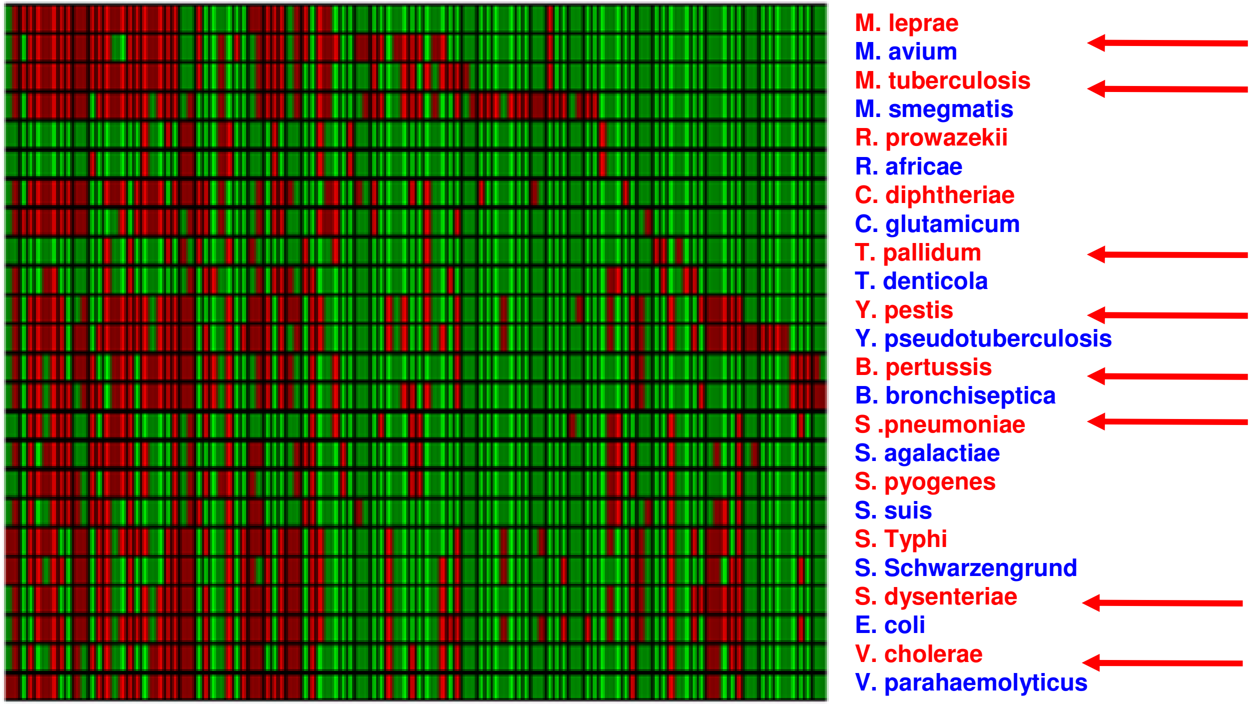

F: Nucleotide transport and metabolism

Figure S11:

Microarray for each functional category

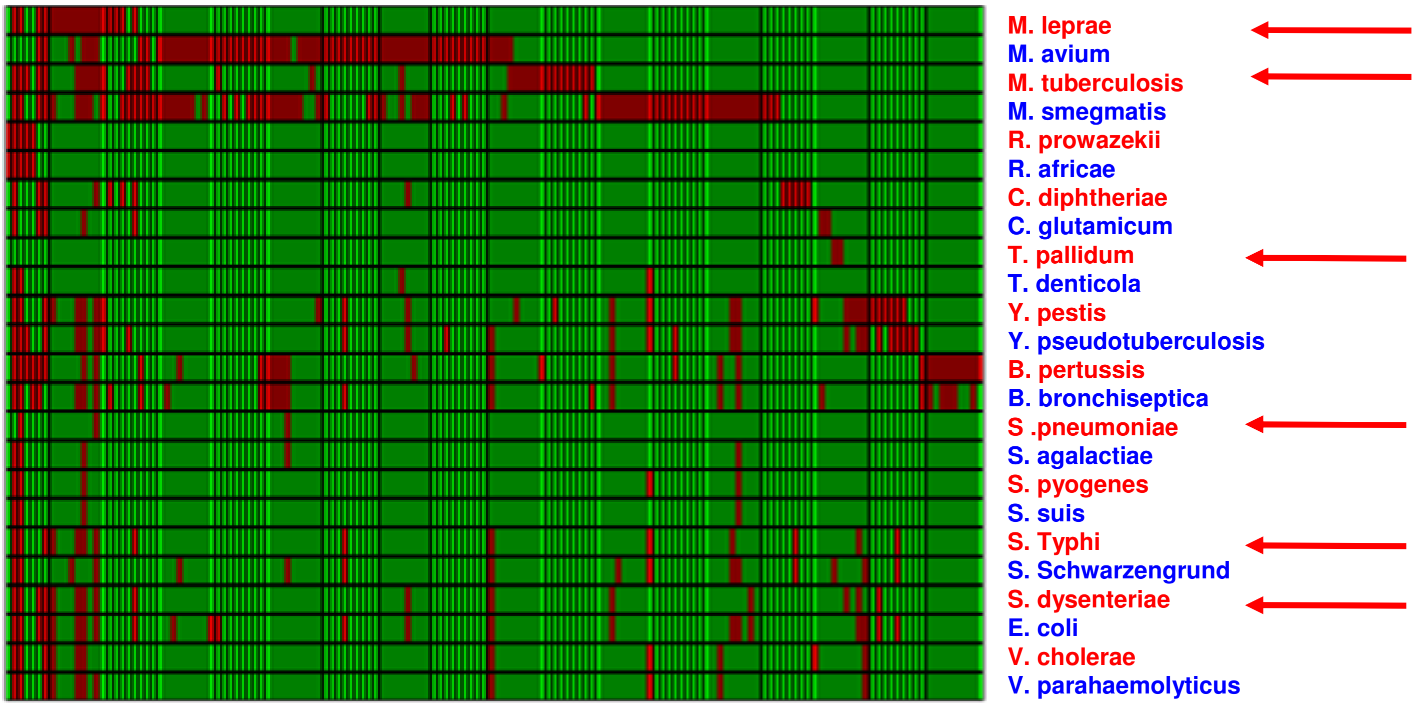

Q: Secondary metabolites transport and metabolism

Figure S11:

Microarray for each functional category

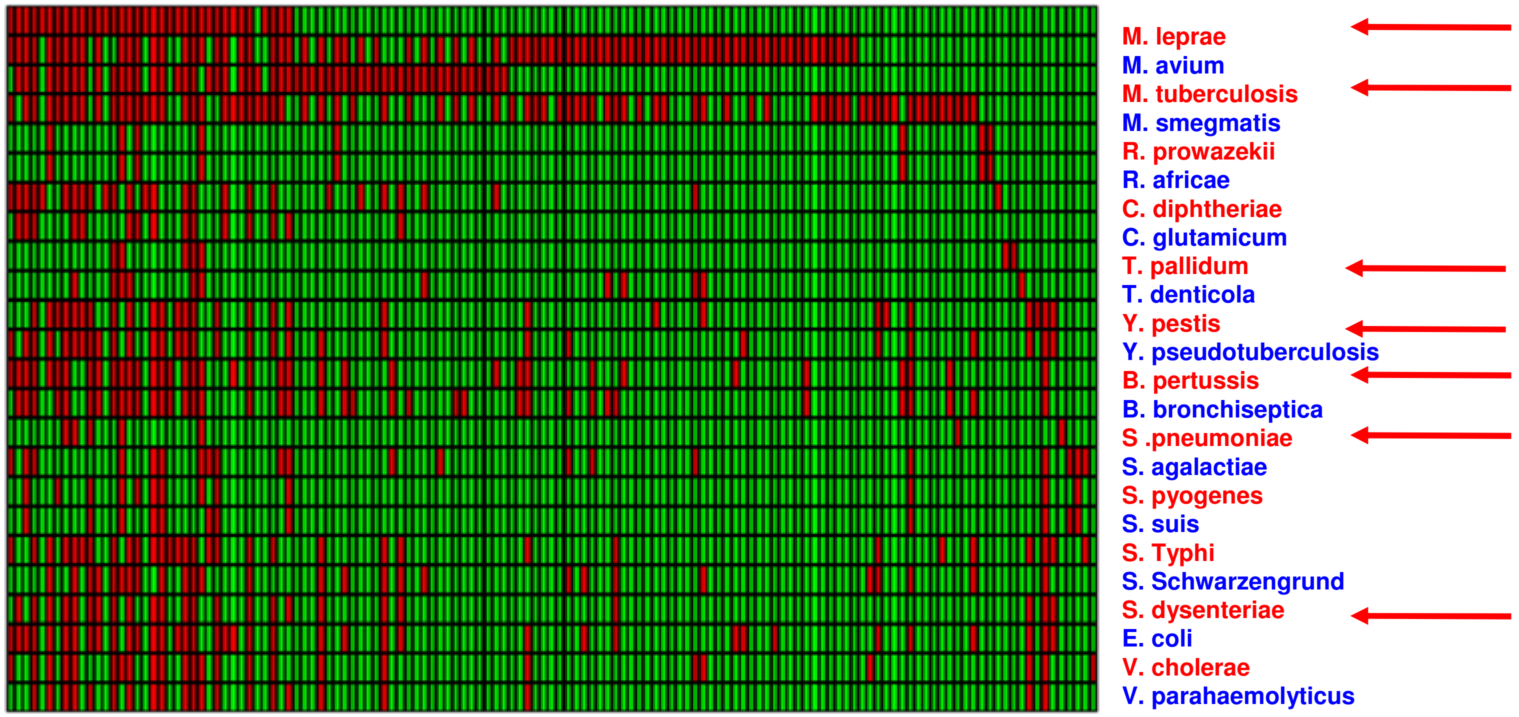

I: Lipid transport and metabolism

Figure S11:

Microarray for each functional category

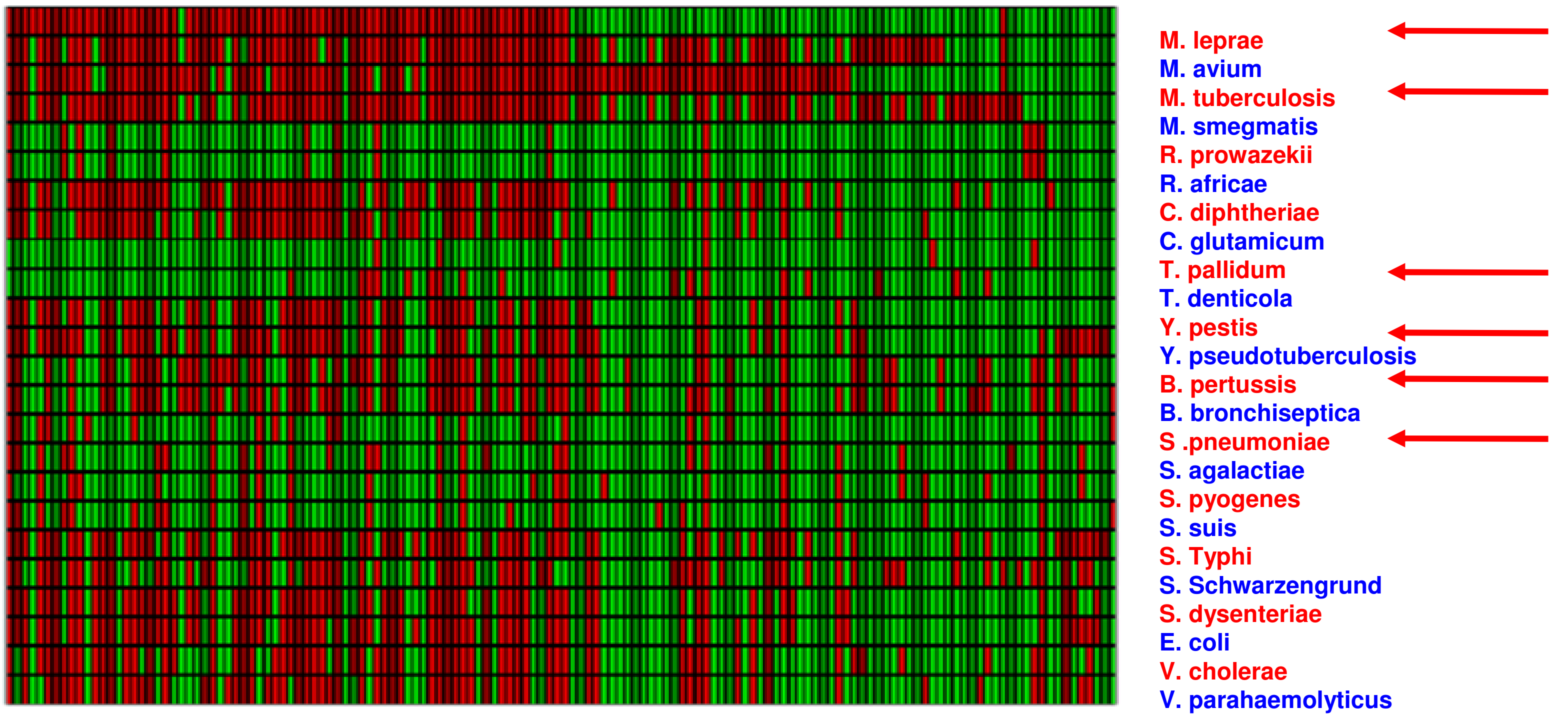

H: Coenzyme transport and metabolism

Figure S11:

Microarray for each functional category

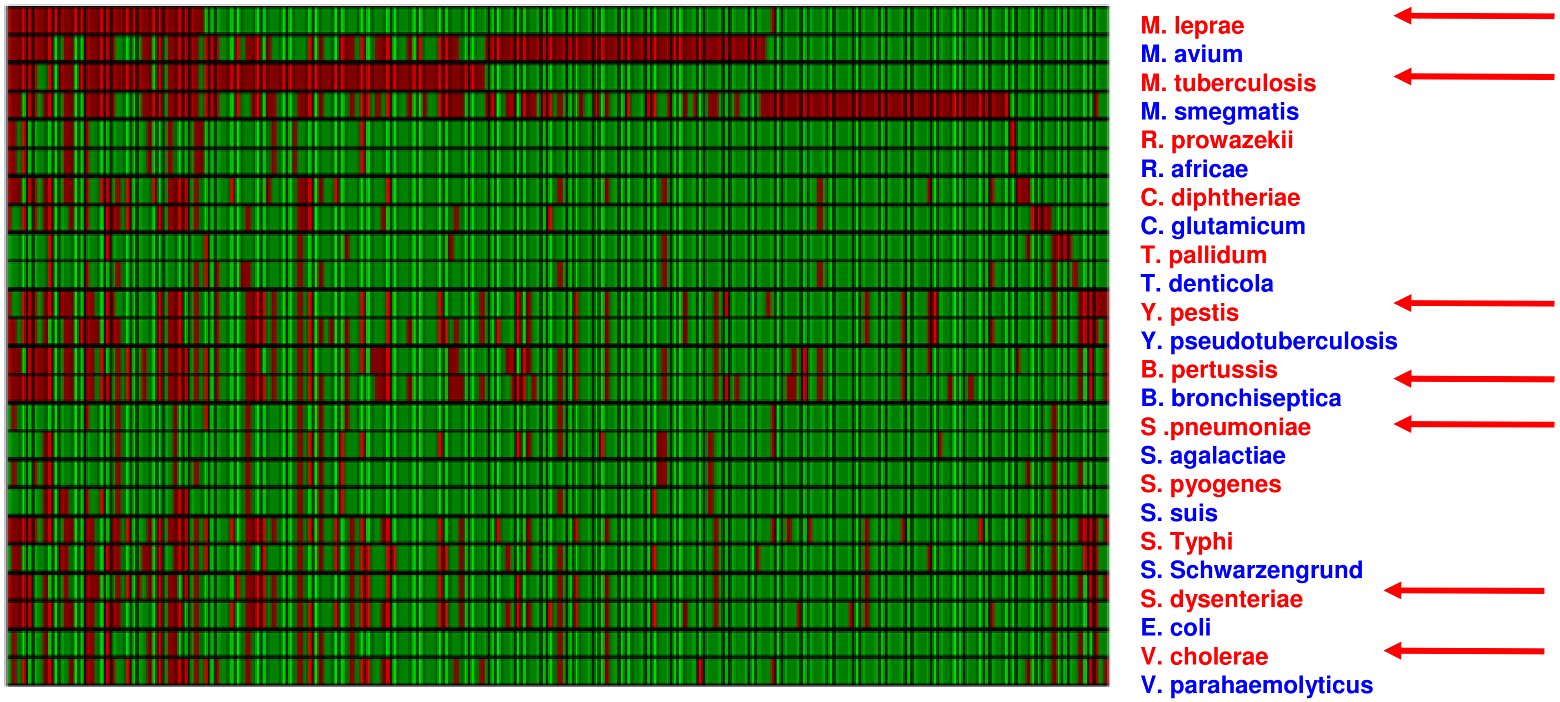

C: Energy production and conversion

Figure S11:

Microarray for each functional category

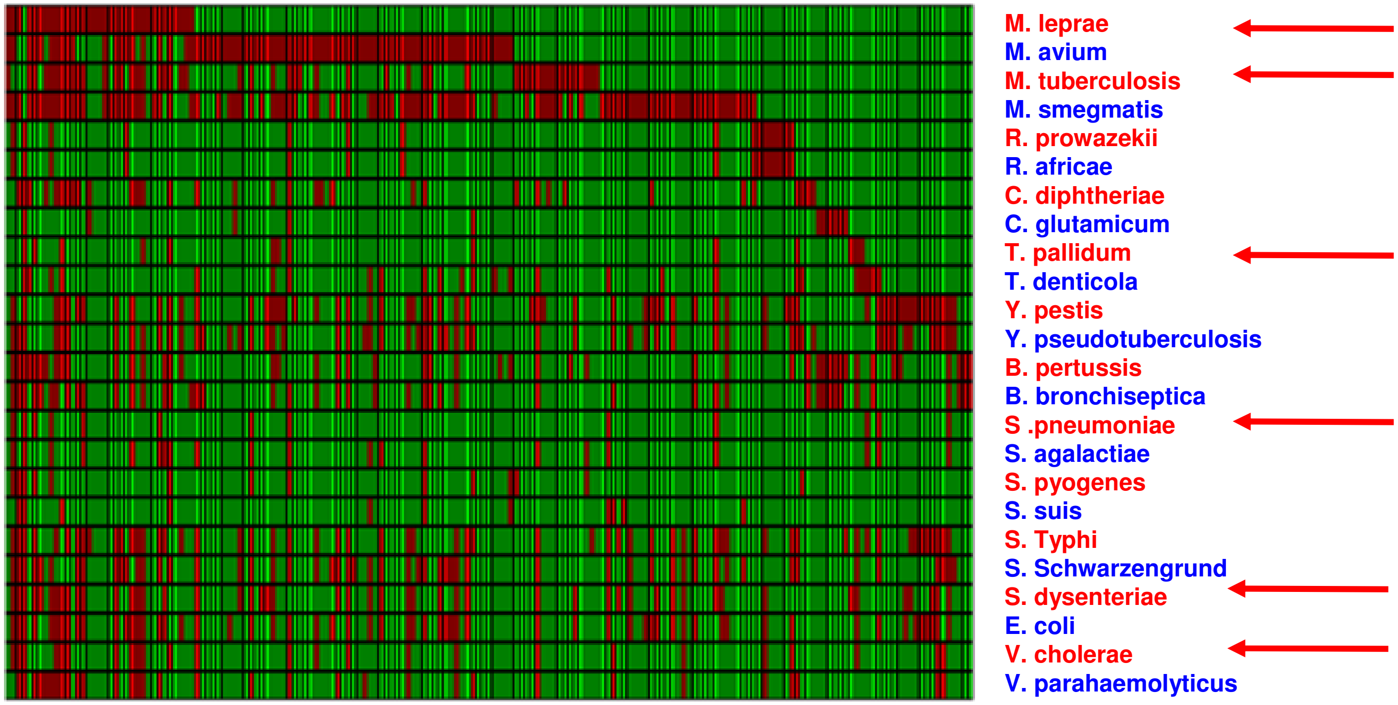

P: Inorganic ion transport and metabolism

Figure S11:

Microarray for each functional category

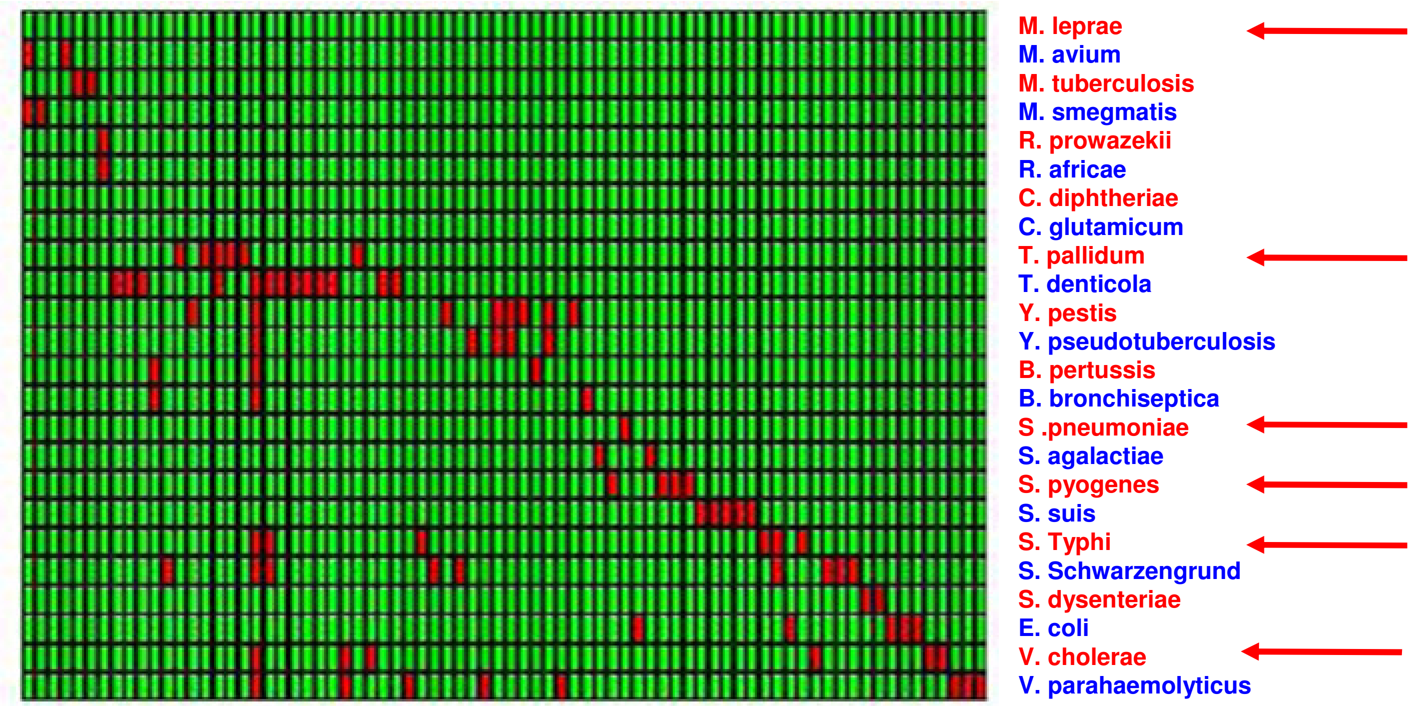

N: Cell motility

Figure S11:

Microarray for each functional category

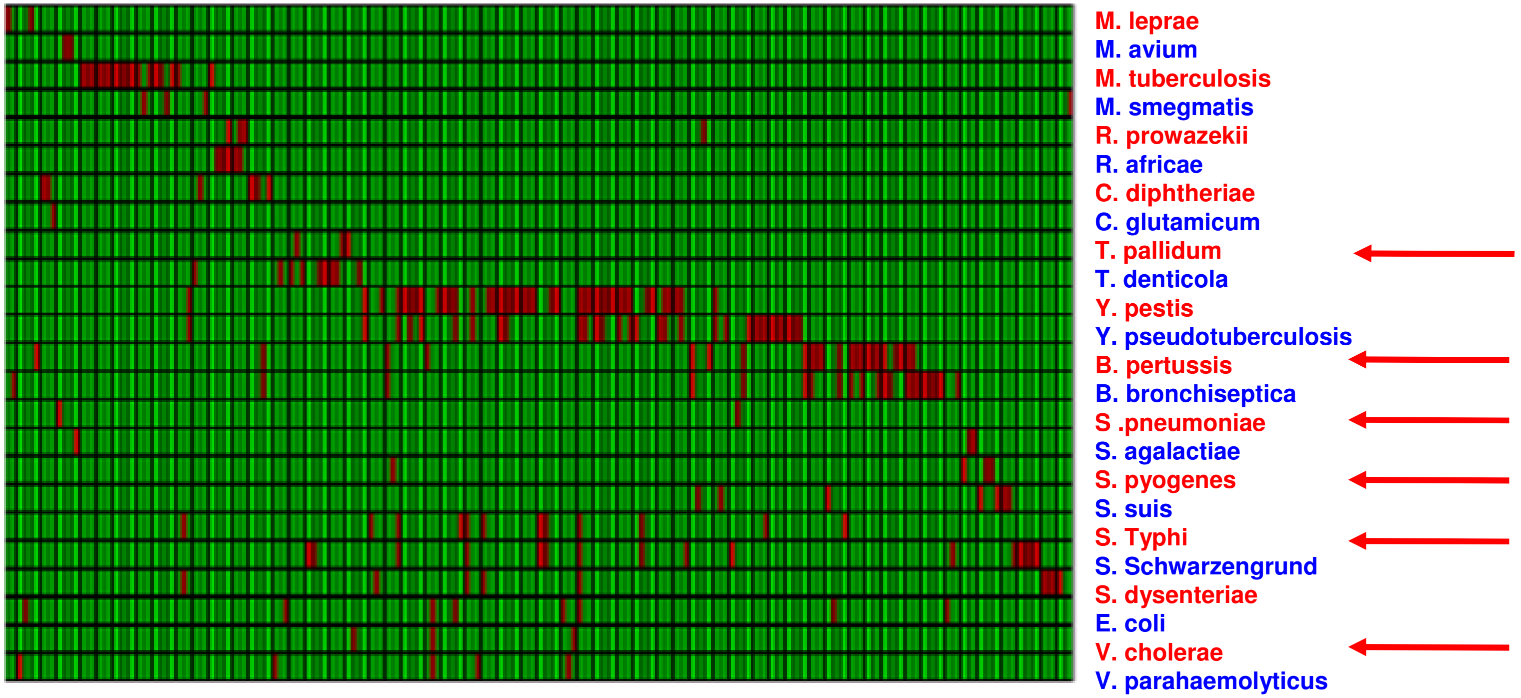

T: Signal transduction mechanisms

Figure S11:

Microarray for each functional category

Functional categories for which bad bugs do not present significantly less genes in each functional category

Figure S11:

Microarray for each functional category

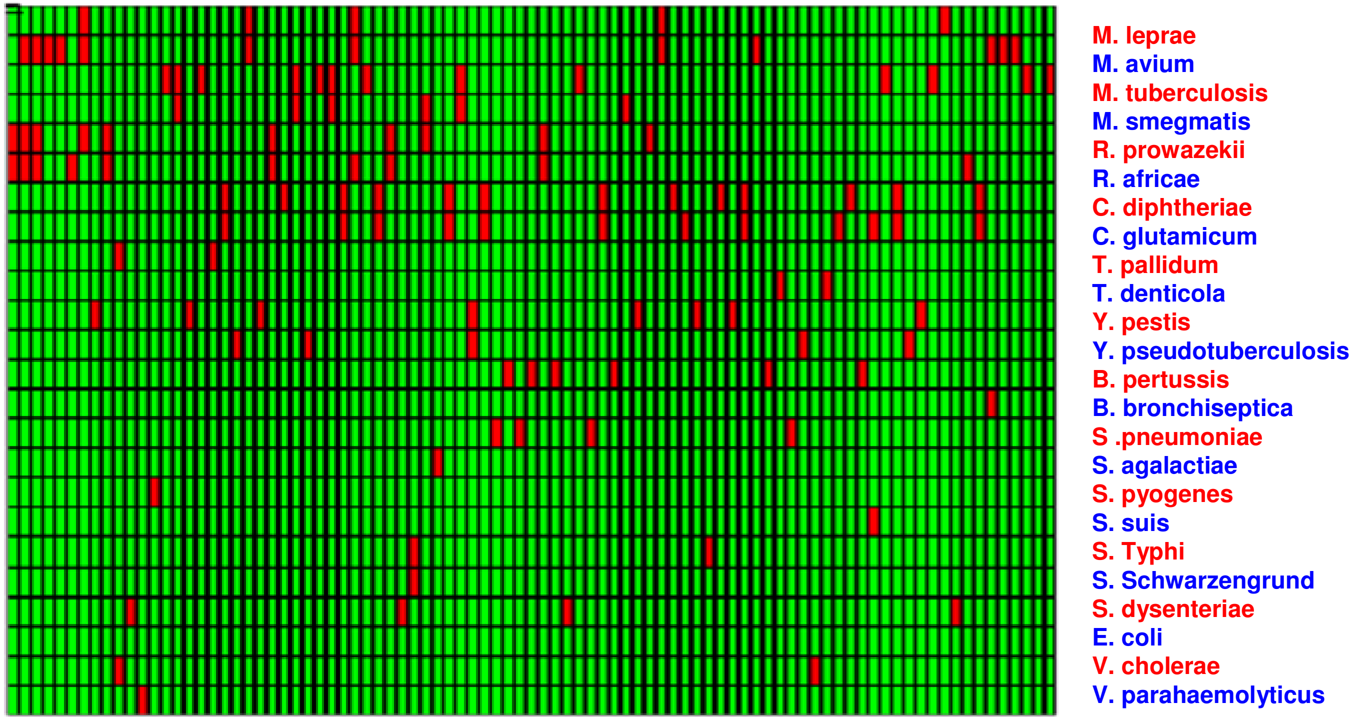

M: Cell wall/membrane biogenesis

Figure S11:

Microarray for each functional category

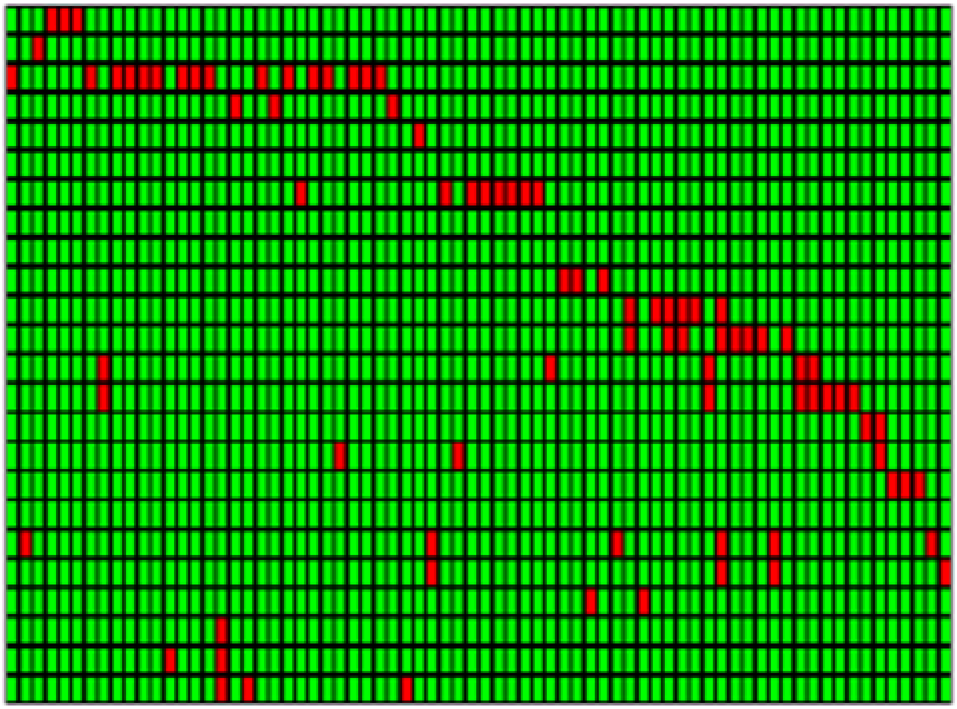

V: Defense mechanisms

Figure S11:

Microarray for each functional category

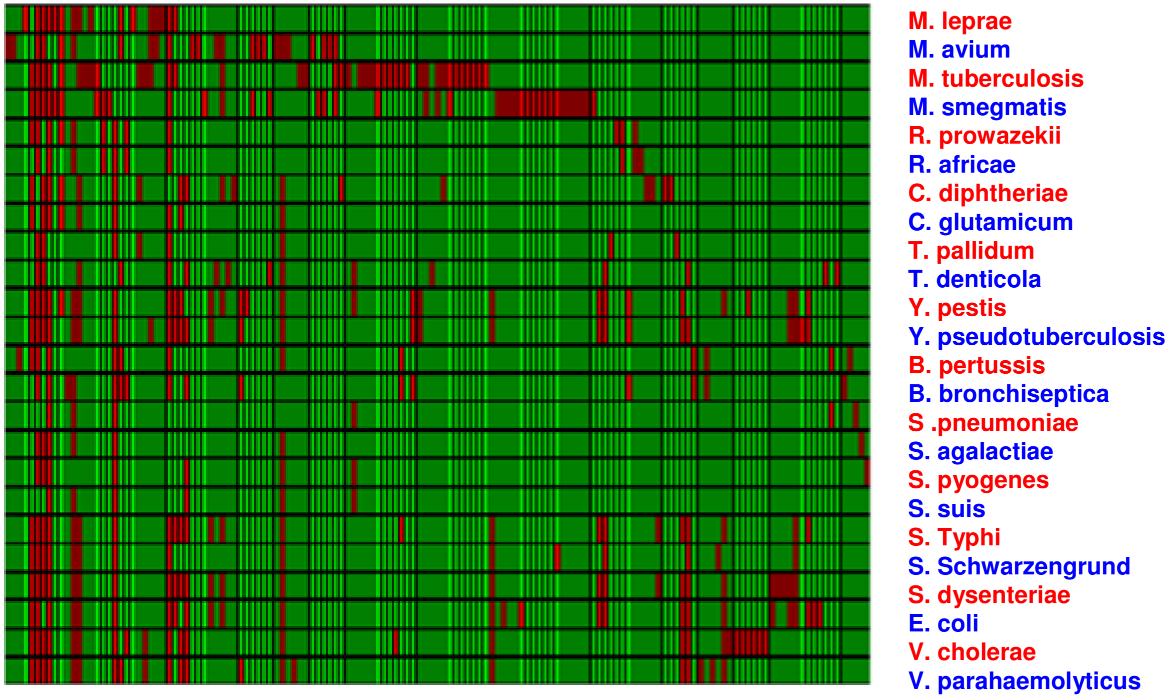

O: Posttranslational modification, protein turnover, chaperones

Figure S11:

Microarray for each functional category

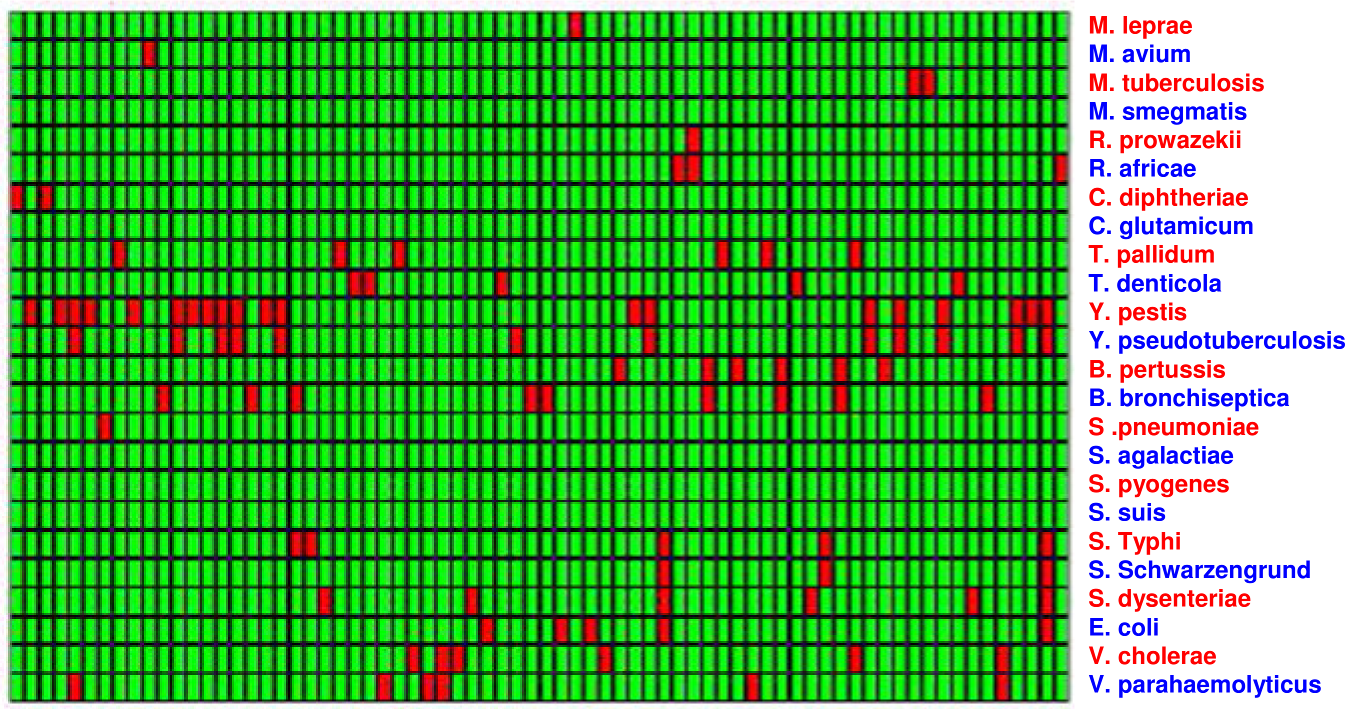

U: Intracellular trafficking and secretion

Figure S11:

Microarray for each functional category

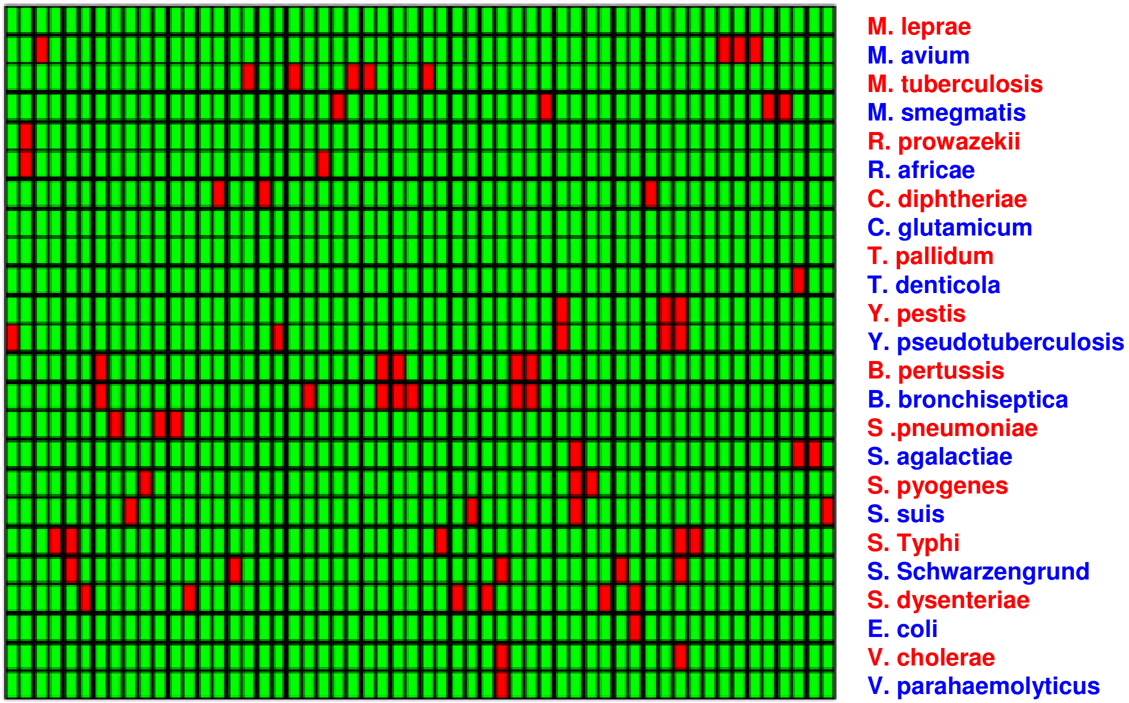

D: Cell cycle control, mitosis and meiosis

Figure S11:

Microarray for each functional category

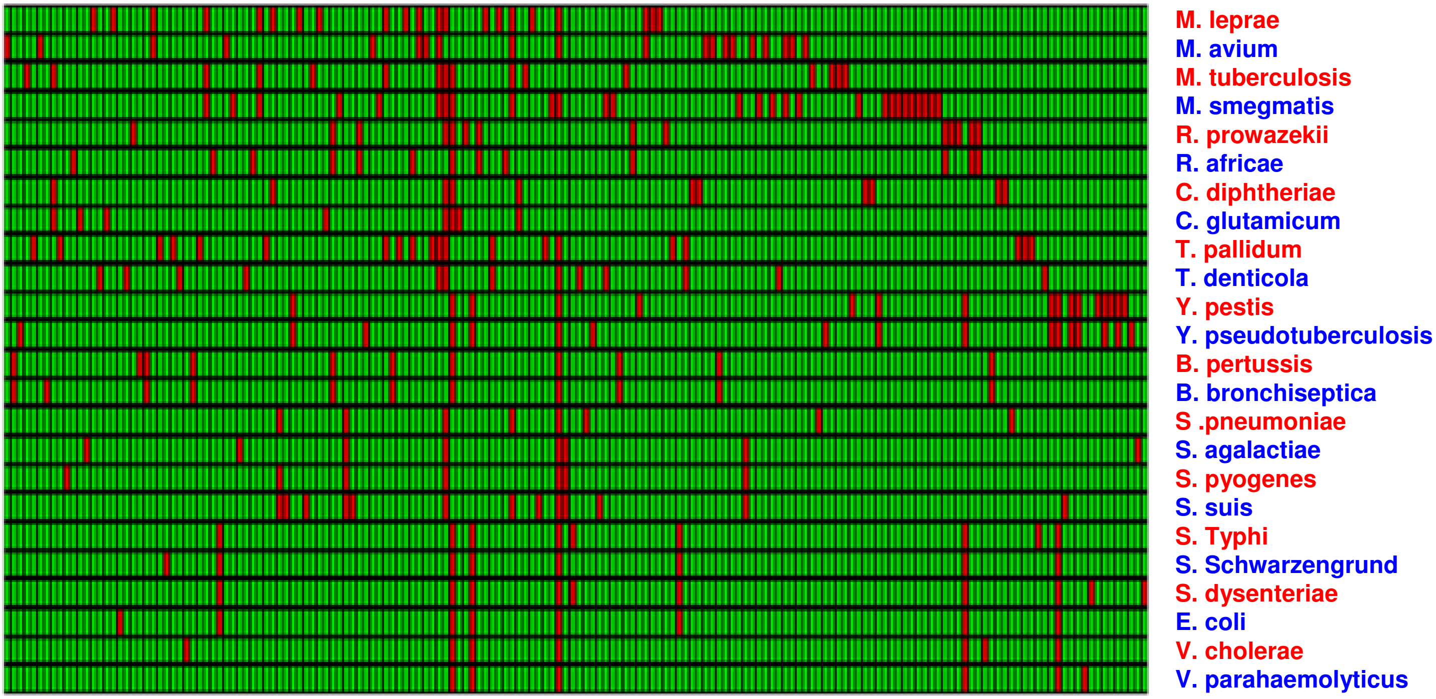

J: Translation

Figure S11:

Microarray for each functional category

Figure S11:

Microarray for each functional category

B: Chromatine structure (white: absence/ pink: presence)
Y: nuclear structure (green: absence)
Z: cytoskeleton (green: absence)
W: extracellular structure (light green: absence/ light red: presence)
A: RNA processing and modification (green absence/ red : presence)

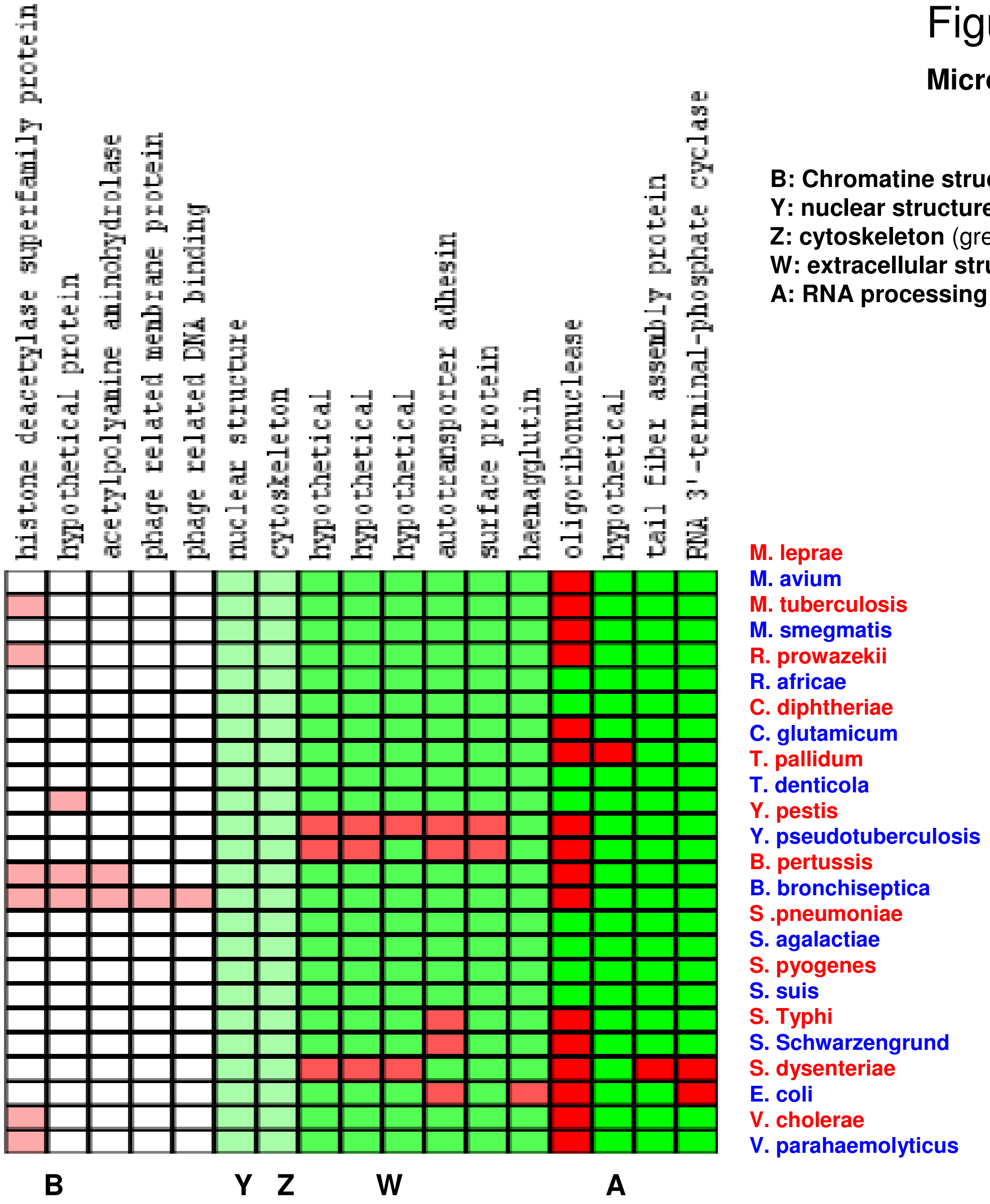

Figure S12:

Phylogenomic clustering of each functional category

Clustering of controls on phylogenomic trees

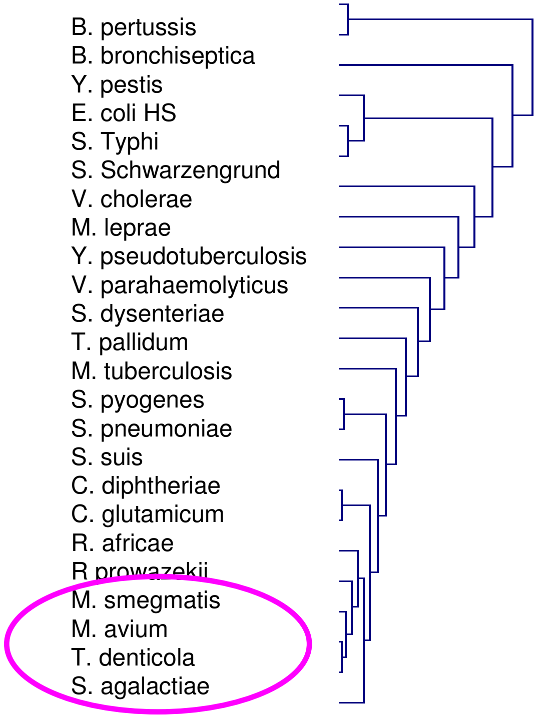

M: Cell wall/membrane biogenesis

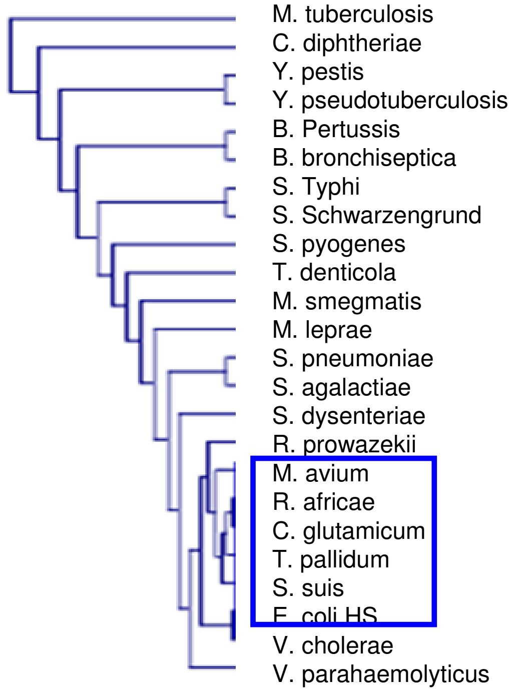

V: Defense mechanisms

Figure S12:

Phylogenomic clustering of each functional category

Figure S12:

Phylogenomic clustering of each functional category

Phylogenomic trees resemble to phylogenetic trees

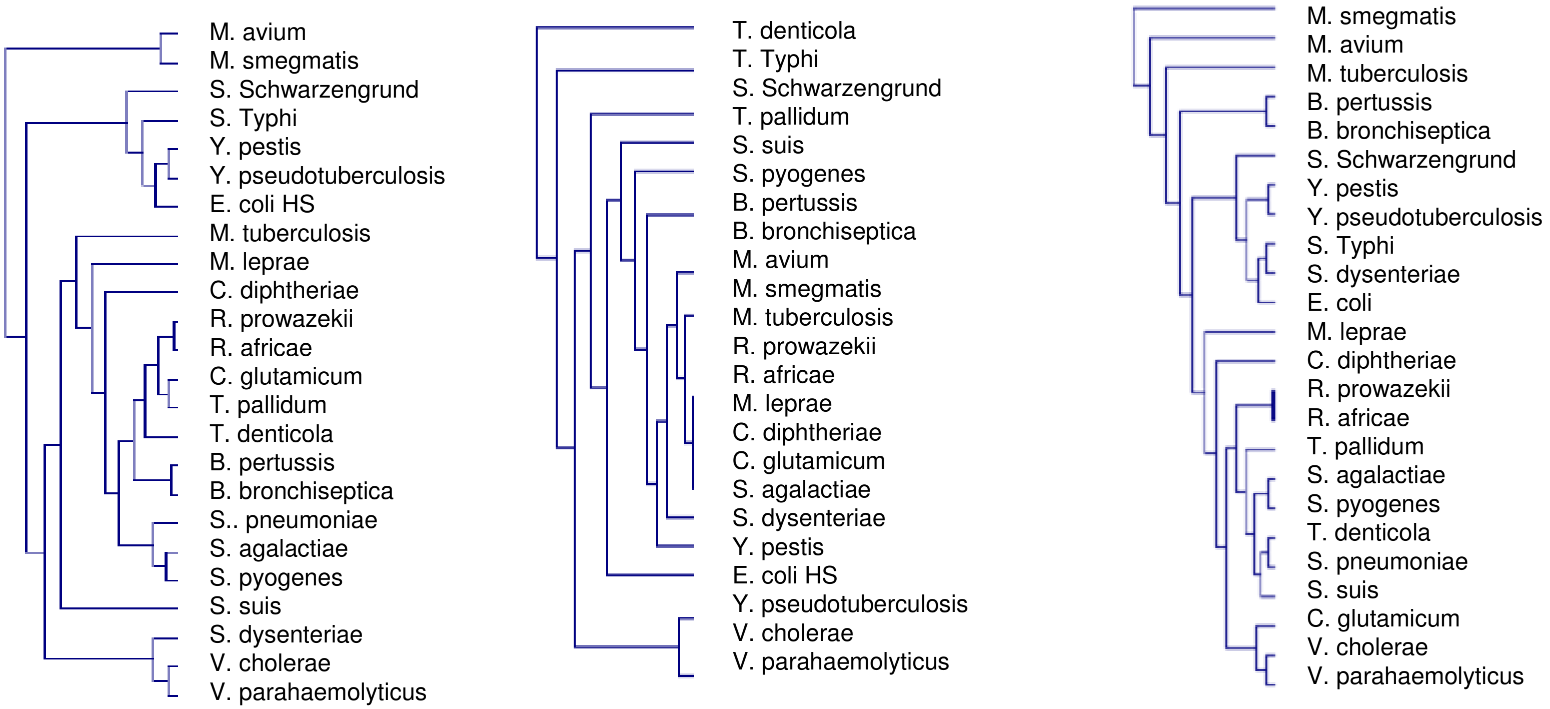

G: Carbohydrate transport and metabolism

N: Cell motility

C: Energy production and conversion

Figure S12:

Phylogenomic clustering of each functional category

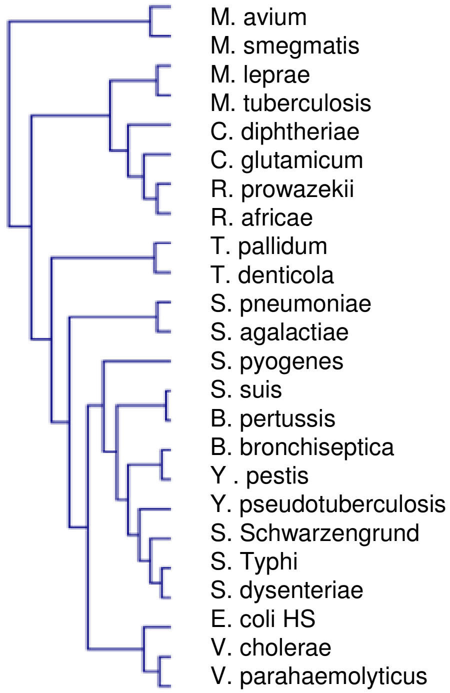

F: Nucleotide transport and metabolism

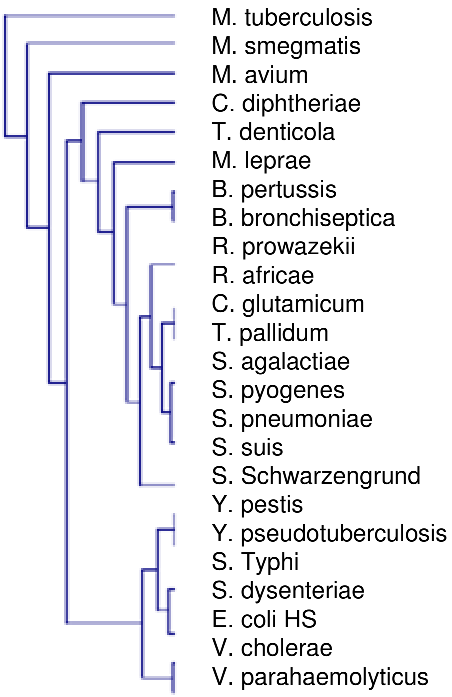

O: Posttranslational modification, protein turnover, chaperones

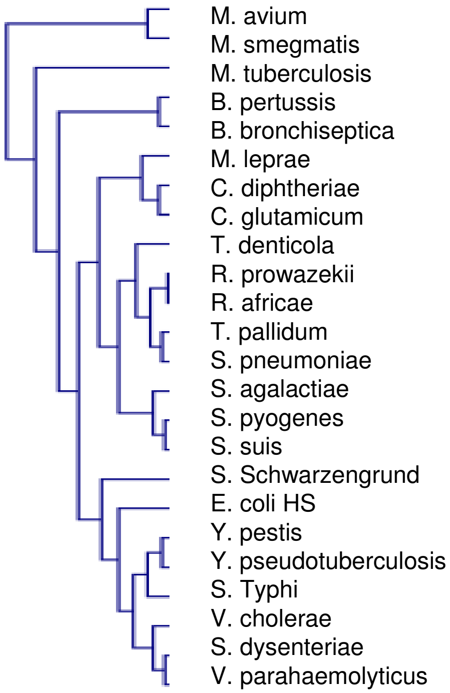

I: Lipid transport and metabolism

Figure S12:

Phylogenomic clustering of each functional category

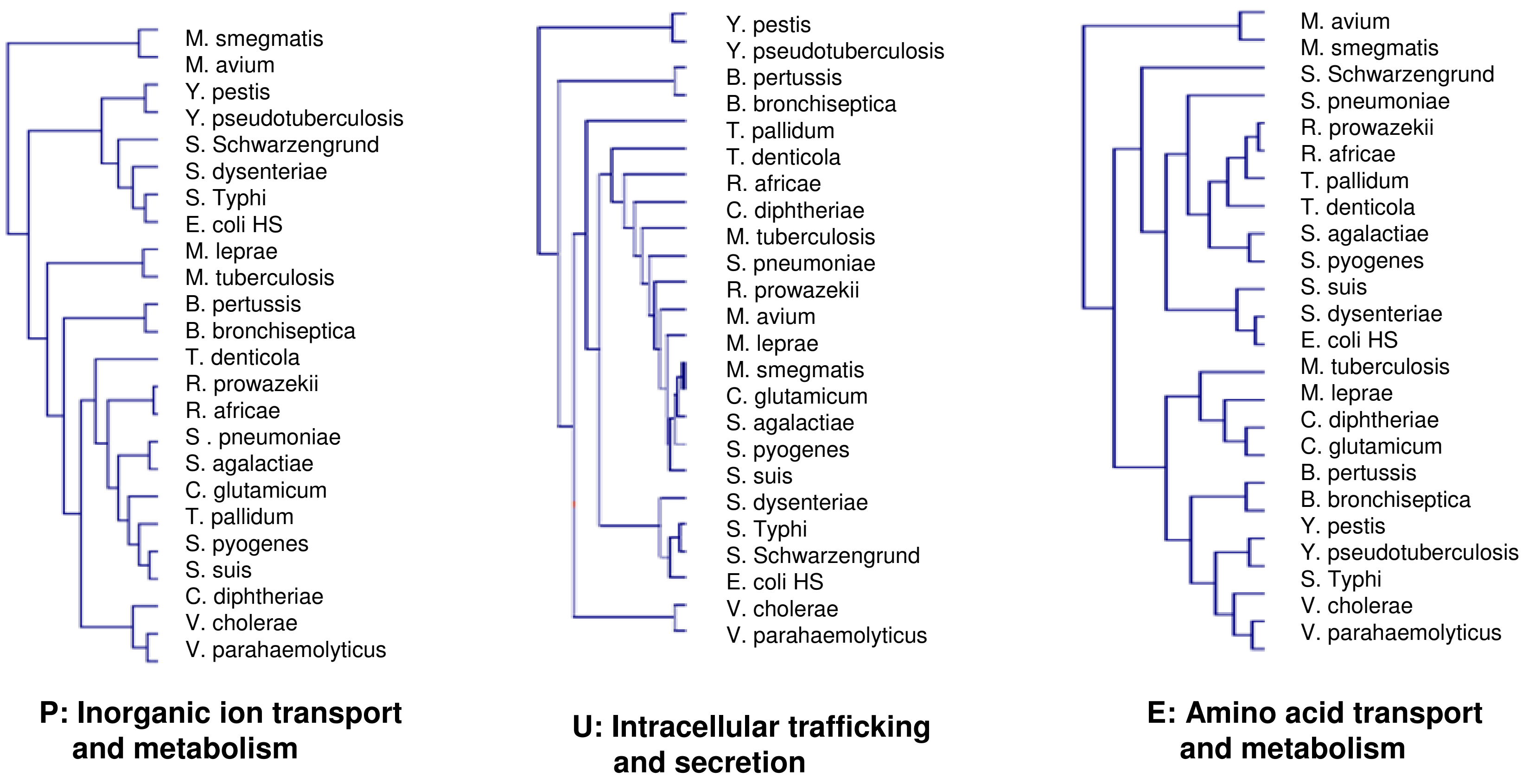

Figure S12:

Phylogenomic clustering of each functional category

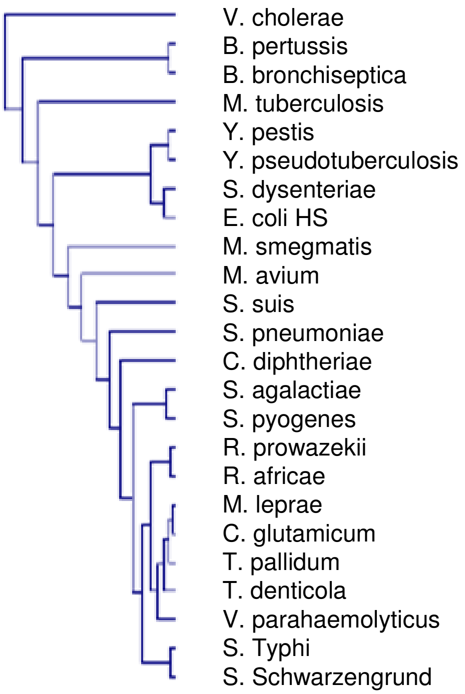

D: Cell cycle control mitosis and meiosis

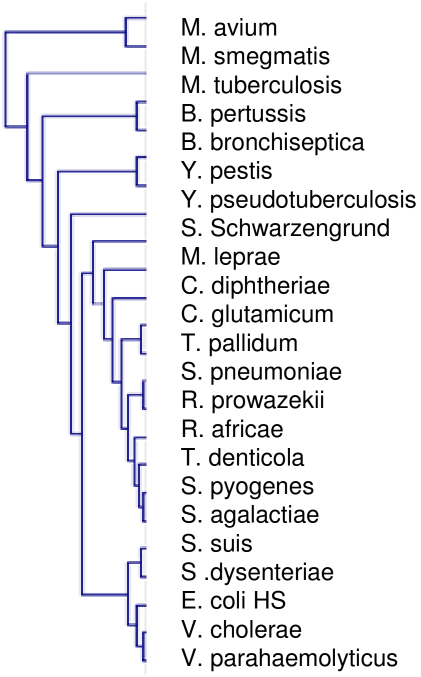

Q: Secondary metabolites transport and metabolism

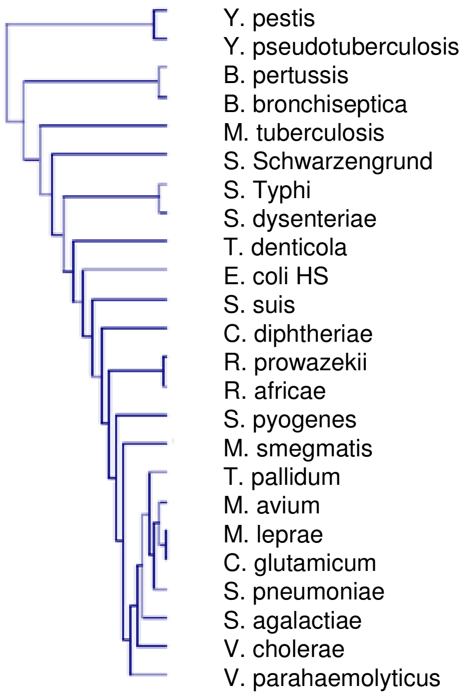

T: Signal transduction mechanisms

Figure S12:

Phylogenomic clustering of each functional category

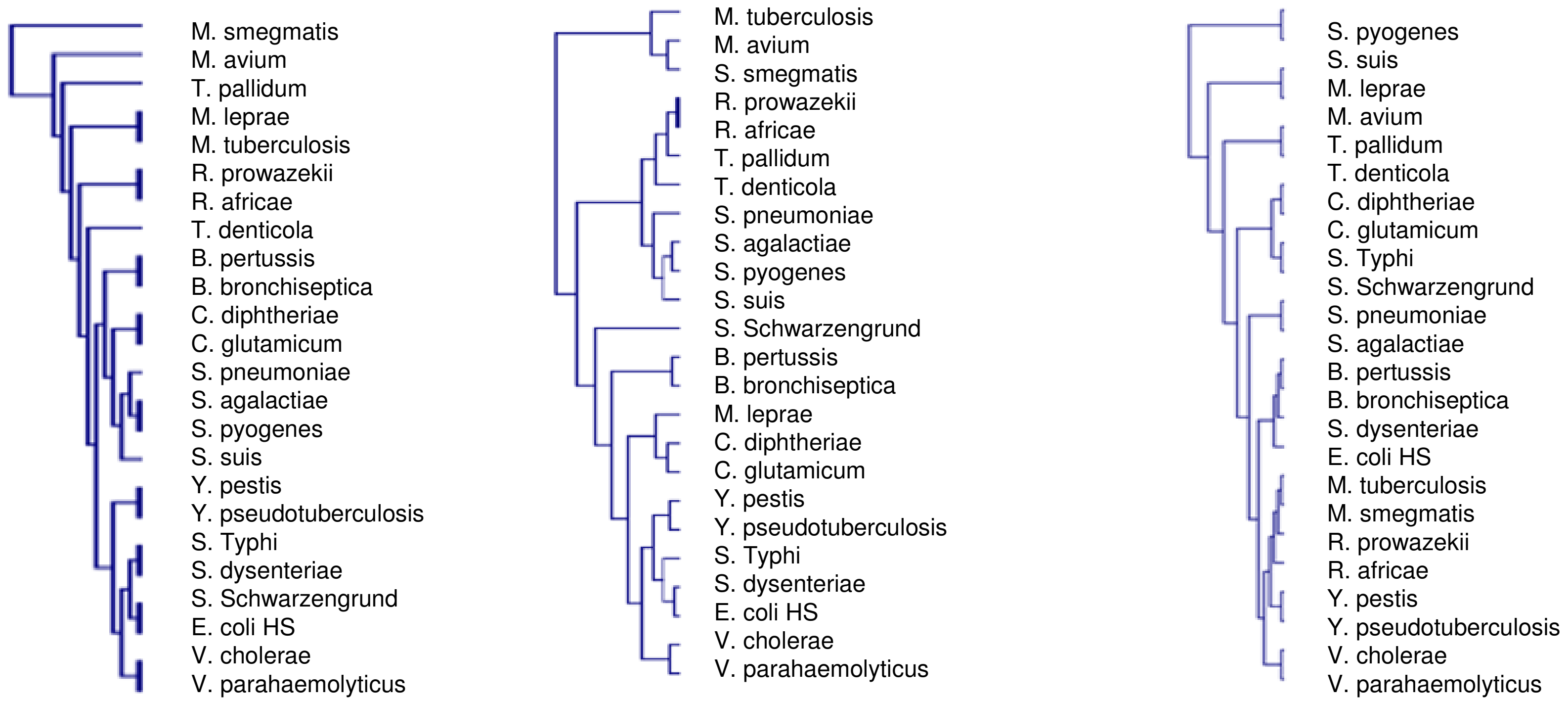

J: Translation

H: Coenzyme transport and metabolism

L: Repair, replication and recombination

Figure S12:

Phylogenomic clustering of each functional category

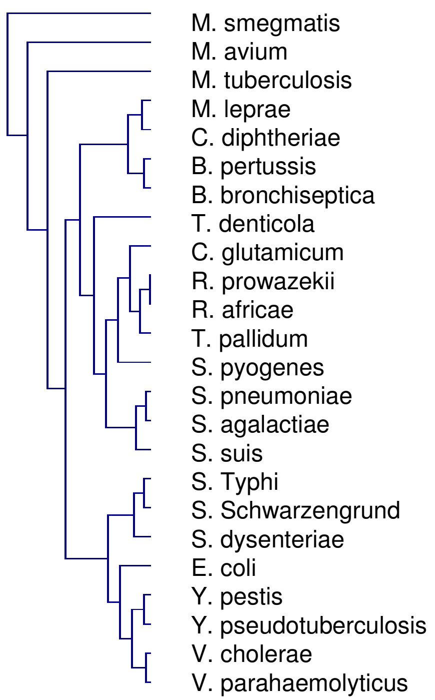

K: Transcription

Figure S12:

Phylogenomic clustering of each functional category

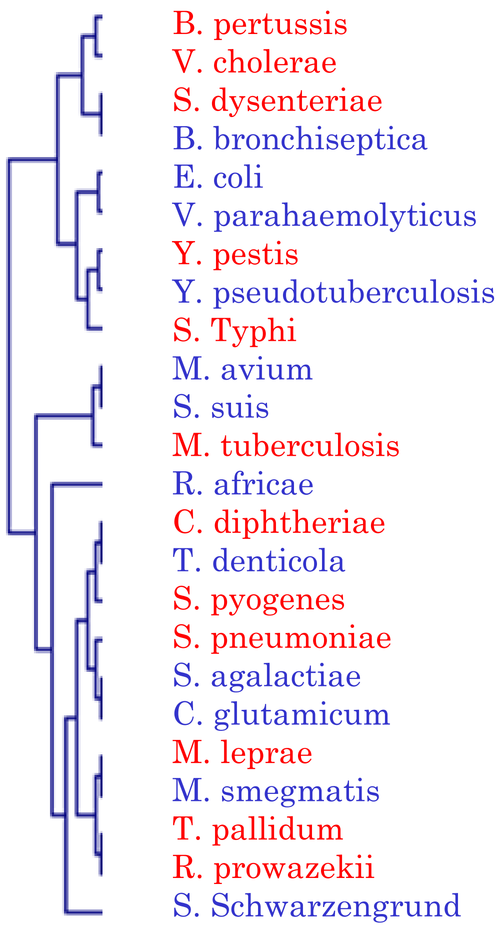

Figure S13: **Phylogenomic clustering of virulence factors**

The “bad bugs” are indicated in red, and the control species are shown in blue.

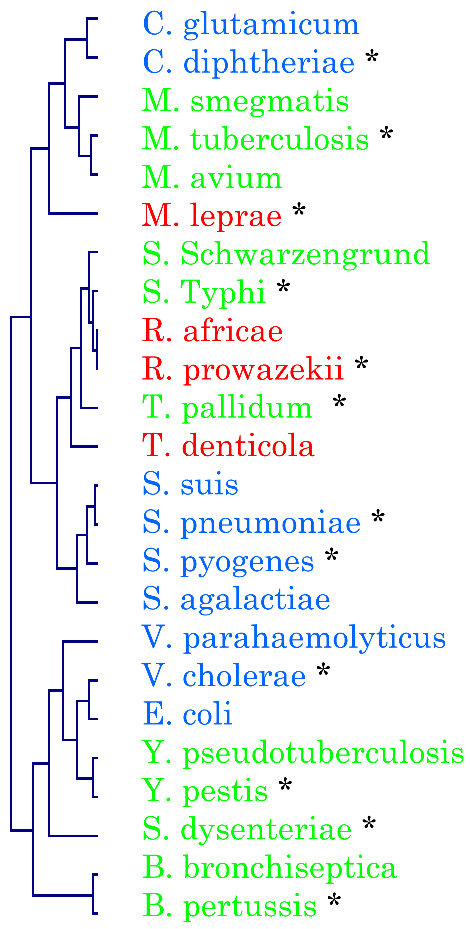

Figure S14:

Phylogenomic clustering of the presence/absence of the set of 100 genes lost from obligate intracellular bacteria. The species in blue are free-living, the species in green are facultative host-dependent, and the species in red are obligate intracellular bacteria. The asterisk denotes “bad bugs”
