# Supplementary material for: Genomes of the Most Dangerous Epidemic Bacteria Have a Virulence Repertoire Characterized by Fewer Genes but More Toxin-Antitoxin Modules
Source: PLoS One. 2011 Mar 18;6(3):e17962. doi: 10.1371/journal.pone.0017962 (PMC3060909; doi:10.1371/journal.pone.0017962)
Supplement: Supporting Information S2 — Supplementary Tables (S1-S13). (PDF) [file pone.0017962.s002.pdf]

	couples	bad bugs	controls
**1**	M. leprae/M. avium	26	68
**2**	M. tuberculosis/M. smegmatis	60	28
**3**	R. prowazekii/R. africae	21	3
**4**	C. diphtheriae/C. glutamicum	31	49
**5**	T. pallidum/T. denticola	19	34
**6**	Y. pestis/Y .pseudotuberculosis	179	192
**7**	B. pertussis/B. bonchiseptica	117	158
**8**	S. pneumoniae/S. agalactiae	44	47
**9**	S. pyogenes/S. suis	38	72
**10**	S. Typhi/S. Schwarzengrund	186	35
**11**	S. dysenteriae/E. coli HS	157	205
**12**	V. cholerae/V. parahaemolyticus	131	202

Table S1. Number of "virulence factors" in the 12 pairs assessed in this study.

Bacteria	Genes encoding eukaryotic-like motifs
M. leprae	**1 U-box domain** (Q9CC35_MYCLE)
M. avium	**1 U-box domain** (A0QKC2_MYCA1)
M. tuberculosis	**1 U-box domain** (O05898_MYCTU)
M. smegmatis	**2 TPR** (A0QT13_MYCS2, A0QY89_MYCS2)<br>**1 ANK** (MSMEG_1266)<br>**2 U-box domain** (A0QTG8_MYCS2, Q8KRQ4_MYCSM)
R. prowazekii	**1 TPR** (RT0587)<br>**3 ANK** (RP226, RP714, RP716)
R. africae	**8 ANK** (raf_ORF0213, raf_ORF0264, raf_ORF0286, raf_ORF0482, raf_ORF0702, raf_ORF0782, raf_ORF0795, raf_ORF0860)
C diphtheriae	**1 ANK** (Q6NFW8_CORDI)<br>**1 U-box domain** (Q6NI57_CORDI)
C. glutamicum	**7 ANK** (A4QFC2_CORGB, A4QG66_CORGB, A4QHW9_CORGB, Q8NLM6_CORGL, Q8NN64_CORGL, Q8NNX2_CORGL, UP10000165DE3)<br>**2 U-box domain** (A4QC82_CORGB, Q8N5C8_CORGL)
T. pallidum	**13 TPR** (007895_TREPA, 083106_TREPA, Y095_TREPA, 083292_TREPA, 083473_TREPA, 083481_TREPA, 083482_TREPA, 083484_TREPA, 083654_TREPA, 083885_TREPA, Y920_TREPA, 083920_TREPA, 083955_TREPA)<br>**2 ANK** (Y502_TREPA, 083807_TREPA)
T. denticola	**27 TPR** (Q73JA3_TREDE, Q73K35_TREDE, Q73KC1_TREDE, Q73KY9_TREDE, Q73KZ24_TREDE, Q73L42_TREDE, Q73LH6_TREDE, Q73M01_TREDE, Q73M24_TREDE, Q73MH2_TREDE, Q73N08_TREDE, Q73N69_TREDE, Q73N91_TREDE, Q73ND5_TREDE Q73NB1_TREDE, Q73NM5_TREDE, Q73NM6_TREDE, Q73NM7_TREDE, Q73NR0_TREDE, Q73P07_TREDE, Q73Q63_TREDE, Q73QJ6_TREDE, Q73QL1_TREDE, Q73QL3_TREDE, Q73QL5_TREDE, Q73R78_TREDE, Q73RP2_TREDE)<br>**6 ANK** (TDE_2138, TDE_2450, TDE_0520, TDE_0552, TDE_2014, TDE_2693)<br>**2 LRR** (Q733KG0_TREDE, Q73Q69_TREDE)
Y. pestis	**3 TPR** (A4TMT9_YERPP, Q7CIL3_YERPP, UP100006DD404)<br>**1 ANK** (Q1CKF5_YERPN)<br>**14 LRR** (A9ZCK3_YERPE, A9ZET9_YERPE, B0A1ZA_YERPE, YOPM_YERPE, Q7CGM7_YERPE, Q7CGM8_YERPE, Q8D0X0_YERPE, B0A3R7_YERPE, BOGK22_YERPE, B0GQ67_YERPE, BOGY75_YERPE, C4GZK9_YERPE, C4GZK7_YERPE, C4H8R8_YERPE, C4HB68_YERPE)<br>**6 U-box domain** (A9Z9F7_YERPE, BOA2L5_YERPE, BOGR75_YERPE, C4H6C9_YERPE, C4HPN9_YERPE, Q8D0I3_YERPE)
Y. pseudotuberculosis	**2 TPR** (A7FFY7_YERPS, A7FI12_YERPS)<br>**1 ANK** (A7FFS8_YERPS)<br>**5 LRR** (A7FEI9_YERPS, A7FEJ0_YERPS, Q663L9_YERPS, Q666F7_YERPS, Q668J8_YERPS)<br>**1 U-box domain** (Q66AE5_YERPS)
B. pertussis	**4 TPR** (Q7VUG8_BORPE, Q7VW81_BORPE, Q7VXV8_BORPE, Q84CS6_BORPE)
B. bronchiseptica	**3 TPR** (Q7WD77_BORBP, Q7WIB7_BORBP, Q7WNY7_BORBP)
S. pneumoniae	**9 U-box domain** (5LDY6_STRPN, A5LP58_STRPN, A5LRZ2_STRPN, A5LZU4_STRPN, A5M9I5_STRPN, A5MI31_STRPN, A5MKB3_STRPN, A5MUL9_STRPN, Q97RR1_STRPN)
S. agalactiae	**1 LRR** (Q2Q1G9_STRAG)
S. pyogenes	**1 LRR** (B0LFQ8_STRPY)<br>**1 U-box domain** (B5I24_STRPZ)
S. suis	**2 TPR** (A4WZS4_STRSU, Q302B7_STRSU)<br>**1 LRR** (B9WUM5_STRSU)<br>**1 U-box domain** (B9WVB0_STRSU)
S. Typhi	**1 LRR** (Q8Z578_SALTI)<br>**1 U-box domain** (Q8Z6R4_SALTI)
S. Schwarzengrund	**1 LRR** (B4TQU4_SALSV)

	**1 U-box domain** (B4TVD2_SALSV)
S. dysenteriae	**5 TPR** (Q32BHO_SHIDS, Q32D24_SHIDS, Q32GQ3_SHIDS, UP1000006733E4, UP1000006735E5)<br>**2 LRR** (B3X564_SHIDY, B3X6G1_SHIDY)<br>**1 U-box domain** (B3WXW5_SHIDY)
E. coli	**1 ANK** (EcHS_A0379)<br>**1 LRR** (A8A027_ECOHS)<br>**1 U-box domain** (A8A0F5_ECOHS)
V. cholerae	**30 TPR** (A1EPE8_VIBCH, A1EPE8_VIBCH, A1EPJ0_VIBCH, A1F7D2_VIBCH, A2P4M4_VIBCH, A2PT69_VIBCH, A2PXG5_VIBCH, A3EJ42_VIBCH, A3ENS9_VIBCH, A3GP76_VIBCH, A3GWT1_VIBCH, A5F321_VIBCH, A5F914_VIBCH, A6A1L5_VIBCH, A6A6N0_VIBCH, A6AGX6_VIBCH, A6AJE1_VIBCH, A6XV93_VIBCH, A6XWT3_VIBCH, A6XZM1_VIBCH, A6Y2A2_VIBCH, A6Y2F5_VIBCH, A6Y2L7_VIBCH, A6Y462_VIBCH, Q9KQT6_VIBCH, Q9KRM4_VIBCH, Q9KS92_VIBCH, Q9KTK1_VIBCH, UP1000005F2FE2, UP1000005F3140, UP1000005F316F)<br>**4 ANK** (A1EPN6_VIBCH, A3EK26_VIBCH, Q9KRQ2_VIBCH, UP1000005F3B34)
V. parahaemolyticus	**18 TPR** (A6AXV6_VIBPA, A6AY66_VIBPA, A6AYK2_VIBPA, A6AZK5_VIBPA, A6B0N7_VIBPA, A6B2L2_VIBPA, A6B3T8_VIBPA, A6AXV6_VIBPA A6B581_VIBPA, A6B8A9_VIBPA, A6B8G3_VIBPA, A6BBA0_VIBPA, Q84157_VIBPA, Q87H96_VIBPA, Q87LB0_VIBPA, Q87M07_VIBPA, Q87N48_VIBPA, Q87P81_VIBPA)<br>**2 ANK** (A6B0E4_VIBPA, Q87JE7_VIBPA)<br>**3 LRR** (A6B2C9_VIBPA, B8K3H5_VIBPA, Q87N90_VIBPA)<br>**5 U-box domain** (A6AX68_VIBPA, A6AZR3_VIBPA, B3IUU9_VIBPA, Q87G92_VIBPA, Q87HJ6_VIBPA)

Table S2. Number of genes encoding eukaryotic-like motifs in bacterial species.
TPR: tetratricopeptide repeats, ANK: ankyrin repeats, LRR: leucine-rich repeats

**Bacteria**	**Number of protein secretion systems**
M. leprae	NO
M. avium	**1 type II/IV secretion protein (COG4962u)**<br>**1 type II secretion system protein F** (COG4965U)
M. tuberculosis	NO
M. smegmatis	**2 type II secretion system protein F (MSMEG_6167, MSMEG_6168)**<br>**1 type I secretion system protein** (MSMEG_6169)
R. prowazekii	**2 type IV secretion system ATPase** (RP103, RP292)
R. africae	**1 type I secretion outer membrane protein** (raf_ORF0284)<br>**8 type IV secretion system ATPase** (raf_ORF0128, raf_ORF0129, raf_ORF0130, raf_ORF0133, raf_ORF0134, raf_ORF0135, DQ821909.1, CP001612.1)
C diphtheriae	NO
C. glutamicum	NO
T. pallidum	NO
T. denticola	NO
Y. pestis	**7 type III secretion system component** (y0515, y0516, y0517, y0518, y0524, y0531, y0532)<br>**2 type III secretion system protein** (y0529, y0530)<br>**1 type III secretion system ATPase** (y0526)<br>**1 type III secretion apparatus protein** (y0527)
Y. pseudotuberculosis	**4 type III secretion system protein** (YPTB0312, YPTB0313, YPTB0326, YPTB0327)<br>**7 type III secretion system apparatus** (YPTB0316, YPTB0317, YPTB0318, YPTB0321, YPTB0324, YPTB0328, YPTB0329)<br>**1 type III secretion system lipoprotein** (YPTB0319)<br>**1 type III secretion system ATPase** (YPTB0323)<br>**1 type IV secretion ATPase** (YPTB3375)
B. pertussis	**1 type II secretion system protein** (BP1996)<br>**12 type III secretion system protein** (BP2235, BP2237, BP2238, BP2239, BP2240, BP2241, BP2244, BP2246, BP2248, BP2249, BP2262, BP2264)<br>**1 type III secretion system ATPase** (BP2245)<br>**1 type III secretion pore protein** (BP2261)
B. bronchiseptica	**7 type II secretion system protein** (BB0780, BB0781, BB0782, BB0784, BB0790, BB1826, BB1828)<br>**12 type III secretion system protein** (BB1609, BB1611, BB1624, BB1625, BB1627, BB1629, BB1631, BB1632, BB1633, BB1634, BB1635, BB1637)<br>**1 type III secretion system ATPase** (BB1628)
S. pneumoniae	NO
S. agalactiae	NO
S. pyogenes	NO
S. suis	NO
S. Typhi	**5 type I secretion system protein** (STY2876, STY2878, STY4456, STY4457, STY4460)<br>**1 type I secretion system protein, ATP-binding** (STY2877)<br>**1 type II secretion system protein** (STY3252)<br>**3 type III secretion system protein** (STY1699, STY1700, STY1701, STY1702, STY1703, STY1704)<br>**1 type III secretion system ATPase** (STY1705)<br>**1 type III secretion system chaperone protein** (STY1721)
S. Schwarzengrund	**3 type I secretion system protein** (SeSA_A2887, SeSA_A2889, SeSA_A4513)<br>**2 type III secretion outer membrane pore** (SeSA_A1491, SeSA_A3051)<br>**7 type III secretion apparatus protein** (SeSA_A1492, SeSA_A1504, SeSA_A1507, SeSA_A3041, SeSA_A1517, SeSA_A3027, SeSA_A3023)<br>**4 type III secretion system protein** (SeSA_A1493, SeSA_A1503, SeSA_A1514, SeSA_A1515)<br>**3 type III secretion system low calcium response chaperone** (SeSA_A1495, SeSA_A1499, SeSA_A3039)<br>**2 type III secretion apparatus needle protein** (SeSA_A1502, SeSA_A3026)

	**2 type III secretion system apparatus lipoprotein** (SeSA_A1505, SeSA_A3024)<br>**1 type III secretion system ATPase** (SeSA_A1511)<br>**6 type IV secretion system protein** (SeSA_A0297, SeSA_A0298, SeSA_A0305, SeSA_A0306, SeSA_A0313, SeSA_A0318)<br>**1 type secretion system lysozyme-related protein** (SeSA_A0299)<br>**1 type IV secretion system ATPase** (SeSA_A0303)<br>**1 type IV secretion system lipoprotein** (SeSA_A0312)
S. dysenteriae	**9 type II secretion system protein** (SDY_3092, SDY_3093, SDY_3094, SDY_3095, SDY_3096, SDY_3097, SDY_3098, SDY_3099, SDY_3100)
E. coli	**2 type III secretion system apparatus** (EcHS_A3009, EcHS_A3011)<br>**1 type III secretion system needle protein** (EcHS_A3010)<br>**1 type IV secretion system protein** (EcHS_A0616)
V. cholerae	NO
V. parahaemolyticus	**14 type III secretion system protein** (VP1664, VP1665, VP1669, VP1671, VP1672, VP1688, VP1689, VP1693, VP1696, VPA1339, VPA1342, VPA1349, VPA1354, VPA1355)<br>**1 type III secretion system ATPase** (VP1668)<br>**2 type III secretion system lipoprotein** (VP1690, VPA1367)<br>**1 type III secretion apparatus** (VPA1335)

Table S3. Number of secretion system proteins in bacterial species.

Bacteria	position of IS	size of IS (bp)	number of operons	of which split
M. leprae	no IS		1	0
M. avium	no IS		1	0
M. tuberculosis	no IS		1	0
M. smegmatis	no IS		2	0
R. prowazekii	no IS		1	1
R. africae	no IS		1	1
C. diphtheriae	5229-5250 *	21	5	0
C. glutamicum	no IS		6	0
T. pallidum	no IS #		2	2
T. denticola	1876-1903<br>4981-5008	27<br>27	2	2
Y. pestis	1651-1690 *	49	7	7
Y. pseudotuberculosis	no IS		7	7
B. pertussis	no IS		3	3
B. bronchiseptica	no IS		3	3
S. pneumoniae	no IS		4	4
S. agalactiae	no IS		7	7
S. pyogenes	5265-5297 *	32	6	6
S. suis	no IS		5	5
S. Typhi	2679-2713 #<br>2723-2788 #<br>3781-3802 #<br>5317-5340 #<br>5371-5400 #	34<br>65<br>27<br>23<br>29	7	7
S. Schwarzengrund	1722-1735<br>1763-1785<br>1804-1814<br>1832-1849<br>1880-1895<br>1962-1974	15<br>22<br>10<br>17<br>15<br>12	3	3
S. dysenteriae	no IS #		7	7
E. coli HS	3121-3143<br>3165-3180	22<br>15	7	7
V. cholerae	1591-1668 *	77	8	8
V. parahaemolyticus	no IS		10	10

Table S4. Position and size of IVSs in the evaluated bacterial species.
*: “bad bugs” with one likely IVS, #: “bad bugs” with fewer IVSs compared to their controls

	Bacteria	Number of Tandem repeats	Size (pb)
**1**	M. leprae	144	2-30
	M. avium	561	6-246
**2**	M. tuberculosis	565	3-257
	M. smegmatis	192	6-231
**3**	C. diphtheriae	75	9-411
	C. glutamicum	92	8-390
**4**	T. pallidum	12	9-96
	T. denticola	82	2-198
**5**	Y. pestis	559	4-477
	Y. pseudotuberculosis	934	5-496
	plasmid 1	6	7-246
	plasmid 2	5	8-78
	chromosome	923	5-496
**6**	B. pertussis	233	6-270
	B. bronchiseptica	233	6-270
**7**	S. pneumoniae *	71	6-213
	S. agalactiae	67	9-293
**8**	S. pyogenes *	42	9-243
	S. suis	16	10-255
**9**	S. Typhi *	94	3-273
	plasmid 1	11	3-273
	plasmid 2	2	15-162
	chromosome	83	12-21
	S. Schwarzengrund	83	3-273
	plasmid 1	3	3-300
	plasmid 2	0	43-106
	chromosome	80	3-300
**10**	S. dysenteriae *	18	8-297
	Plasmid 1	1	9
	Plasmid 2	1	21
	chromosome	16	8-297
	E. coli	30	14-380
**11**	V. cholerae *	84	6-438
	chromosome 1	36	6-438
	chromosome 2	48	6-414
	V. parahaemolyticus	81	6-480
	chromosome 1	58	6-480
	chromosome 2	23	6-429

Table S5. Number and size of tandem repeat sequences in bacteria.
*: “bad bugs” carrying more tandem repeat sequences compared to their controls

Bacteria	Small RNAs Accession	Number of sRNAs
M. leprae	RF00380, RF00515, RF00379	**3**
M. avium	RF00379, RF00380, RF00442, RF00515	**4**
M. tuberculosis	RF00080, RF00379, RF00380, RF00515	**4**
M. smegmatis	-	**0**
R. prowazekii	-	**0**
R. africae	-	**0**
C. diphtheriae	RF00080	**1**
C. glutamicum	RF00080, RF00379	**2**
T. pallidum	**-**	**0**
T. denticola	-	**0**
Y. pestis	RF00018,RF00021,RF00034, **2** RF00057,RF00078, RF00079,RF00081, RF00082,RF00101,RF00110, RF00111,RF00112,RF00127, RF00128,RF00369,RF01339, **2** RF01396, **19** RF01405	**37**
Y. pseudotuberculosis	RF00018, RF00021, RF00034, RF00057, RF00078, RF00079, RF00081, RF00082, RF00110, RF00111, RF00112, RF00127, RF00128, RF00368, RF00369, RF01394, RF01396, **2** RF01405,	**19**
B. pertussis	RF00140, RF01070	**2**
B. bronchiseptica	RF00140, RF00624, RF01068, RF01070	**4**
S. pneumoniae	RF00230, RF00515	**2**
S. agalactiae	RF00029, RF00235	**2**
S. pyogenes	RF00029, RF00235	**2**
S. suis	Listeria snRNA rli28	**1**
S. Typhi	RF00014, RF00018, RF00021, RF00034, RF00035, **2** RF00057, RF00077, RF00078, RF00079, RF00081, RF00082, RF00084, RF00101, RF00110, RF00111, RF00112, **3** RF00113, RF00118, RF00120, RF00121, RF00124, RF00126, RF00127, **2** RF00128, RF00368, RF00369, RF00370, RF00505, RF01388, RF01390, RF01391, RF01394, RF01397, RF01398, RF01399, RF01401, RF01402, RF01403, RF01404, **18** RF01405, RF01406, RF01407, RF01409	**64**

S. Schwarzengrund	RF00018, RF00021, RF00035, **2** RF00057, RF00077, RF00078, RF00081, RF00101, RF00110, RF00111, RF00112, **3** RF00113, RF00118, RF00126, RF00127, **2** RF00128, RF00369, RF00370, RF00505, RF01388, RF01390, RF01391, RF01393, **2** RF01396, RF01397, RF01398, RF01399, RF01401, RF01402, RF01403, RF01404, RF01406, RF01407, RF01409	**39**
S. dysenteriae	RF00014, RF00018, RF00021, RF00034, RF00035, RF00057, RF00079, RF00082, RF00084, RF00101, RF00110, RF00111, RF00115, RF00116, RF00117, RF00118, RF00119, RF00120, RF00121, RF00124, RF00126, RF00368, RF00369, RF00370, RF00372, RF01394, RF01405, RF01407	**28**
E. coli	RF00014, RF00018, RF00034, **6** RF00035, **2** RF00077, RF00078, RF00082, RF00101, RF00112, RF00113, RF00115, RF00116, RF00117, RF00119, RF00125, RF00126, RF00127, RF00128, RF00368, RF00369, RF00370, RF00372, RF00505	**29**
V. cholerae	**2** RF00018, RF00021, RF00057, RF00111, RF00368, **4** RF00378, RF01394	**11**
V. parahaemolyticus	**2** RF00018, RF00021, RF00057, RF00111, RF00127, RF00368, **5** RF00378	**12**

Table S6. Number of sRNAs in “bad bugs” and in controls.

**Bacteria**	**Number of TA systems**
M. leprae	0
M. avium	1
M. tuberculosis *	38
M. smegmatis	0
R. prowazekii	0
R. africae	10
C. diphtheriae	0
C. glutamicum	1
T. pallidum	0
T. denticola	1
Y. pestis *	5
Y. pseudotuberculosis	0
B. pertussis	0
B. bronchiseptica	0
S. pneumoniae *	5
S. agalactiae	2
S. pyogenes *	2
S. suis	0
S. Typhi *	6
S. Schwarzengrund	1
S. dysenteriae *	2
E. coli	1
V. cholerae *	13
V. parahaemolyticus	4

Table S7. Number of toxin-antitoxin (TA) systems in "bad bugs" and in controls.
*: "bad bugs" having more TA systems compared to their controls

	Protein	CG %	GC% ref.	found in	function	Functional category
	**Specific non-pathogen**					
**1**	Trans-2-enoyl-CoA-reductase	62.7%	67% (M. smegmatis)	α-proteobacteria, Actinobacteria, nematodes, flies, primates	oxidoreductase	Energy production and onversion
**2**	Thioredoxin reductase	69%	67% (M. smegmatis)	Actinobacteria, α/δ-proteobacteria, cyanobacteria	oxidoreductase	Posttranslational modification

A

	Protein	CG%	CG% ref.	originated species	found in	function	Functional category
**1**	Rhamnolipids biosynthesis – 3-oxoloacyl reductase	66.3%	67% (M. smegmatis)	fungi	γ/α-proteobacteria, Actinobacteria, Spirochetes	Rhamnolipids biosynthesis	Lipid transport and metabolism
**2**	Cinnamoyl-ester-hydrolase	52%	37.9% (T. denticola)	Firmicutes	Bacteroidetes, Spirochetes	hydrolase	Lipid transport and metabolism
**3**	Pentachlorophenol-4-monooxyganase	67.2%	67% (M. smegmatis)	Actinobacteria	β/δ-proteobacteria,, Firmicutes	oxidoreductase	Energy produnction and conversion
**4**	6-hydroxy-D-nicotine oxidase	68.1%	67% (M. smegmatis)	fungi	α/β-proteobacteria, Actinobacteria	oxidoreductase	Energy production and conversion
**5**	Methionin-S-oxide-reductase	53%	53% (T. pallidum)	δ-proteobacteria or Bacillus	α/β/γ-proteobacteria, Spirochetes, Actinobacteria, fungi, protists, Archaea	oxidoreductase	Posttranslational modification

B

Table S8. Specific control species genes (A) and HGT (B).

**Bacteria**	**photoreactivation**	**Σ**	**Mismatch excision repair**						**Σ**	**base excision repair**			**Σ**	**nucleotide excision repair**					**Σ**
	**PhrB**		**MutS**	**MutL**	**MutH**	**UvrD**	**SbcB**	**MutT**		**Ung**	**MutY**	**MutM**		**UvrA**	**UvrB**	**UvrC**	**UvrD**	**MFD**	**4**
M. leprae	0	**0**	0	0	0	0	1	0	**1**	0	1	1	**2**	1	1	1	0	1	**4**
M. avium	1	**1**	0	0	0	1	1	1	**3**	0	1	0	**1**	1	1	1	1	0	**5**
M. tuberculosis	0	**0**	0	0	0	1	0	1	**2**	1	1	0	**2**	1	1	1	1	1	**5**
M. smegmatis	1	**1**	0	0	0	1	0	0	**1**	1	1	1	**3**	1	1	1	1	1	**4**
R. prowazekii	0	**0**	0	0	0	1	0	0	**1**	0	0	0	**0**	0	1	1	1	1	**4**
R. africae	0	**0**	0	0	0	1	0	0	**1**	0	0	0	**0**	0	1	1	1	1	**4**
C. diphtheriae	0	**1**	0	0	0	0	0	0	**0**	1	0	0	**1**	1	1	1	0	1	**4**
C. glutamicum	0	**1**	0	0	0	0	0	0	**0**	1	1	0	**2**	1	1	1	0	1	**3**
T. pallidum	0	**0**	0	0	1	0	0	1	**2**	0	0	1	**1**	1	1	1	0	0	**5**
T. denticola	0	**0**	1	0	0	1	0	1	**3**	0	0	0	**0**	1	1	1	1	1	**5**
Y. pestis	1	**1**	1	1	1	1	1	1	**6**	1	1	0	**2**	1	1	1	1	1	**5**
Y.pseudotuberculosis	1	**1**	0	0	1	1	1	1	**4**	1	0	0	**1**	1	1	1	1	1	**5**
B. pertussis	0	**0**	1	1	1	1	0	0	**4**	0	1	1	**2**	1	1	1	1	1	**5**
B. bronchiseptica	0	**0**	1	1	1	1	0	0	**4**	0	1	1	**2**	1	1	1	1	1	**5**
S. pneumoniae	0	**0**	1	1	1	1	0	0	**4**	1	1	1	**3**	1	1	1	1	1	**5**
S. agalactiae	0	**0**	1	1	1	1	0	0	**4**	0	0	0	**0**	1	1	1	1	1	**4**
S. pyogenes	1	**1**	1	1	1	0	0	0	**3**	1	1	0	**2**	1	1	1	0	1	**5**
S. suis	0	**0**	1	1	0	1	0	1	**4**	1	0	1	**2**	1	1	1	1	1	**5**
S. Typhi	1	**1**	1	1	0	1	1	1	**5**	1	1	0	**2**	1	1	1	1	1	**5**
S. Schwarzengrund	1	**1**	1	1	1	1	1	1	**6**	1	1	1	**3**	1	1	1	1	1	**5**
S. dysenteriae	1	**1**	1	1	1	1	1	1	**6**	0	1	1	**2**	1	1	1	1	1	**5**
E. coli	1	**1**	1	1	1	1	1	1	**6**	1	1	1	**3**	1	1	1	1	1	**5**
V. cholerae	1	**1**	1	1	1	1	1	1	**6**	1	1	0	**2**	1	1	1	1	1	**5**
V.parahaemolyticus	1	**1**	1	1	1	1	1	1	**6**	1	1	0	**2**	1	1	1	1	1	**4**

	**Recombinational repair**										**Σ**	**Other repair**				**Σ**
	**RecB**	**RecC**	**RecD**	**SbcB**	**SbcC**	**SbcD**	**RecA**	**RecR**	**RecO**	**RecN**		**UmuC**	**UmuD**	**LexA**	**RadA**	
M. leprae	0	0	0	1	0	0	0	1	0	0	**2**	0	0	1	1	**2**
M. avium	1	1	0	1	0	0	1	0	0	0	**4**	0	0	0	1	**1**
M. tuberculosis	1	1	1	0	0	0	1	0	0	0	**4**	0	0	1	1	**2**
M. smegmatis	0	0	1	0	0	0	1	0	0	0	**2**	0	1	1	1	**3**
R. prowazekii	0	0	0	0	0	0	1	1	1	1	**4**	0	0	0	1	**1**
R. africae	0	0	0	0	0	0	1	1	1	1	**4**	0	0	0	1	**1**
C. diphtheriae	0	0	0	0	0	1	1	0	0	0	**2**	0	0	1	1	**2**
C. glutamicum	1	0	0	0	0	0	1	1	0	0	**3**	0	0	1	1	**2**
T. pallidum	0	0	0	0	0	0	1	0	0	0	**1**	0	1	0	1	**2**
T. denticola	0	0	0	0	1	1	1	1	0	0	**4**	0	0	0	1	**1**
Y. pestis	1	1	1	1	1	1	1	0	0	0	**7**	1	0	1	1	**3**
Y.pseudotuberculosis	1	1	1	1	1	1	1	0	0	0	**7**	0	0	1	1	**2**
B. pertussis	0	0	0	0	1	0	1	1	0	0	**3**	0	0	1	1	**2**
B. bronchiseptica	0	0	0	0	1	0	1	1	0	0	**3**	0	0	1	1	**2**
S. pneumoniae	0	0	0	0	0	0	1	0	0	0	**1**	0	0	1	1	**2**
S. agalactiae	0	0	1	0	0	0	1	1	0	0	**3**	0	0	1	1	**2**
S. pyogenes	1	1	1	0	0	1	1	1	0	0	**6**	0	0	1	1	**2**
S. suis	0	0	1	0	0	0	1	1	0	0	**3**	1	0	0	1	**2**
S. Typhi	1	1	0	1	1	1	1	0	0	0	**6**	1	1	1	1	**4**
S. Schwarzengrund	1	1	1	1	1	1	1	0	0	0	**7**	1	1	1	1	**4**
S. dysenteriae	1	1	1	1	1	0	1	0	0	0	**6**	1	1	1	1	**4**
E. coli	1	1	1	1	1	1	1	0	0	0	**7**	1	1	1	1	**4**
V. cholerae	0	1	1	1	0	0	1	0	0	0	**4**	0	0	1	1	**2**
V.parahaemolyticus	1	1	1	1	0	0	1	0	0	0	**5**	0	0	1	1	**2**

Table S9. Database of repair, replication and recombination genes.
1: gene present, 0: gene absent, Σ: total number of genes

		Species			
**Networks**	**Genes**	**Bad bugs**	**Controls**	**Functions**	**Functional category**
**1**	KatE<br>SodA<br>starvation induced DNA protecting protein<br>Soj		M. avium<br>E. coli<br>M. avium/ M. smegmatis<br>M. avium	catalase HPII<br>superoxide dismutase<br>response to stress<br>regulator protein	inorganic ion transport and **metabolism**<br>cell cycle control
**2**	2-oxo-hepta-3-ene-1,7-dioic hydratase<br>feruloyl-CoA synthetase		M. avium/M. smegmatis/ E. coli<br>M. avium/M. smegmatis	aromatic compound catabolism<br>catalitic activity	secondary metabolites transport and **metabolism**
**3**	Xaa-Pro dipeptidase<br>Oxygenase KshA<br>Rieske [2Fe-2S]domain containing protein		M. avium/M. smegmatis<br>M. avium<br>M. avium	dipeptidase activity<br>oxidoreductase activity<br>oxidoreductase activity	secondary metabolites transport and **metabolism**<br>inorganic ion transport and **metabolism**
**4**	TobE protein<br>ThiS family protein		M. avium/M. smegmatis<br>C. glutamicum	Transport	coenzyme transport and **metabolism**
**5**	Threonine synthase<br>LtaE<br>Tat-translocated enzyme		T. denticola<br>S. agalactiae/ S. suis<br>M. avium/ Y. pseudotuberculosis/ S. pneumoniae	amino acid biosynthesis<br>L-threonine aldolase<br>iron iron binding	amino acid transport and **metabolism**<br>inorganic ion transport and **metabolism**
**6**	Histone deacetylase<br>argR (arginine repressor)		M. avium<br>E. coli	transcription regulation<br>transcription regulation	chromatine structure<br>transcription
**7**	PemK family protein<br>CutC		T. denticola<br>T. denticola	DNA-binding<br>copper homeostasis protein	signal transduction mecanisms<br>inorganic ion transport and **metabolism**
**8**	dam2<br>integral membrane protein<br>chromate transport protein		R. africae<br>T. denticola	site-specific DNA alanine methylase<br>membrane protein<br>transporter activity	replication, recombination<br>general prediction<br>inorganic ion transport and **metabolism**
**9**	plasmid maintenance system antidote protein<br>6-aminohexanoate-cyclic dimer hydrolase		R africae<br>M. avium/M. smegmatis	DNA binding<br>hydrolase activity	general prediction<br>translation
**10**	putative virulence protein<br>oxygen-independent coproporphyrinogen		R. africae	pathogenesis<br>oxidoreductase	General prediction<br>coenzyme transport and **metabolism**
**11**	TcpJ (type 4 prepilin leader peptide processing)<br>TcpA (toxin coregulated pilus A)	V. cholerae<br>V. cholerae		endopeptidase activity<br>pathogenesis	intracellular trafficking<br>cell motility

Table S10. Networks containing proteins that are either found only in the controls (1-7) or only in the "bad bugs" (8) of each pair.

		Species			
	**Genes**	**Bad bugs**	**Controls**	**Functions**	**Functional category**
**1**	VirS	M. tuberculosis		virulence regulating transcriptional regulator	Transcription
**2**	2-component response regulator	V. cholerae		transcription regulator	Transcription
**3**	plasmid replication protein	C. diphtheriae		protein polymerisation	Transcription
**4**	DNA-binding bacteriophage	C. diphtheriae			Transcription
**5**	SigI	M. tuberculosis		transcription initiation	Transcription
**6**	lantibiotic ABC transporter ATP-binding	C. diphtheriae		bacteriocin transport	defense mechanisms
**7**	DNA methylesterase	C. diphtheriae			defense mechanisms
**8**	HsdS restriction modification system	C. diphtheriae		DNA methylase	defense mechanisms
**9**	drug transport protein	M.leprae/M.tuberculosis			defense mechanisms
**10**	penicillin binding protein	M. tuberculosis		penicillin binding	defense mechanisms
**11**	lipoprotein	M. tuberculosis		pathogenesis	defense mechanisms
**12**	FliS protein	Y. pestis		flagellum assembly	intracellular trafficking
**13**	peptidase	S. Typhi			cell cycle control
**14**	protein methionine-S-oxide reductase	T. pallidum		oxidation reduction	Posttranslationnal modifications
**15**	tail fiber assembly protein	S.dysenteriae/V.cholerae			RNA processing and modification
**16**	vibriobactin and enterobactin ABC transporter	V. cholerae		high affinity iron ion transport	inorganic ion transport and metabolism
**17**	phosphoribosylpyrophosphate synthase	V. cholerae			amino acid transport and metabolism
**18**	nickel hydrogenase incorporation		M. avium/M. smegmatis/C. glutamicum	cofactor biosynthetic process	Transcription
**19**	ATP-dependent helicase DING		E. coli	DNA recombination	Transcription
**20**	SigJ		M. avium	transcription initiation	Transcription
**21**	diphtheria toxin repressor		M. avium	regulation of transcription	Transcription

**22**	DNA-directed RNA pol. Su beta'		E. coli	regulation of transcription	Transcription
**23**	transcriptional regulator		R. africae	regulation of transcription	Transcription
**24**	SpoT15		R. africae	Guanosine polyphosphate pyrophosphate hydrolase	Transcription
**25**	Ankyrin repeat		R. africae	protein-protein interaction	General prediction
**26**	flagellar assembly protein H (FliH)		V. parahaemolyticum	flagellar motility	intracellular trafficking
**27**	inner membrane protein		T. denticola		intracellular trafficking
**28**	outer membrane usher protein precursor		E. coli		intracellular trafficking
**29**	chaperone protein		E. coli	response to stress	intracellular trafficking
**30**	hemolysin activator like		E. coli/V. parahaemolyticum		intracellular trafficking
**31**	type IV prepillin peptidase		V. parahaemolyticum		intracellular trafficking
**32**	transmembrane protein		M. avium		cell cycle control
**33**	nucleotide binding protein		T. denticola		cell cycle control
**34**	cell division initiation		S. suis		cell cycle control
**35**	MukB		S. schwarzengrund	chromosome partition protein	cell cycle control
**36**	septum site determining		E. coli		cell cycle control
**37**	UDP-N-acetylglycosamine acyltransferase		E. coli		cell wall biogenesis
**38**	ABC transporter ATP-binding		S. suis/B. bronchiseptica		cell wall biogenesis
**39**	large conductance mechanosensitive channel		M. avium	Ion transport	cell wall biogenesis
**40**	capsular polysaccharide biosynthesis glycos		E. coli	polysaccharide biosynthetic protein	cell wall biogenesis
**41**	major facilitator superfamily protein		M. smegmatis		signal transduction
**42**	PspC domain containing protein		T. denticola		signal transduction
**43**	P22 repressor		Y.		signal transduction

			pseudotuberculosis		
44	HTH-DNA binding domain containing		E. coli	transcriptional regulator	signal transduction
45	mannitol-2-dehydrogenase		M. avium	oxidoreductase	carbohydrate transport and metabolism
46	HAMP domain/ EAL domain protein		T. denticola	signal transducer activity	cell motility
47	pentachlorophenol-4-monooxygenase		M. smegmatis	oxidation reduction	energy production and conversion
48	6-hydroxy-D-nicotine oxidase		M. smegmatis	oxidation reduction	energy production and conversion
49	trans-2-enoyl-CoA-reductase		M. smegmatis	oxidoreductase	energy production and conversion
50	Dihydropyrimidinase		M. smegmatis	dihydropyrimidinase activity	nucleotide transport and metabolism
51	thioredoxin reductase		M. smegmatis	oxidoreductase	Posttranslationnal modifications
52	cinnamoyl-ester hydrolase		T. denticola	hydrolase activity	lipid metabolism and transport
53	para-nitrobenzylesterase		M. avium		lipid metabolism and transport
54	rhamnolipids biosynthesis 3-oxaloacyl reduct		M. avium		lipid metabolism and transport
55	phage related membrane protein		B. bronchispetica		Chromatine structure
56	phage related DNA-binding		B. bronchispetica		Chromatine structure
57	autotransporter adhesin		E. coli		extracellular structure
58	Hemagglutin		E. coli		extracellular structure
59	von Willebrand factor type A domain containin		M. avium/M. smegmatis		inorganic ion transport and metabolism
60	superoxide dismutase		E. coli	oxidation reduction	inorganic ion transport and metabolism
61	NADH dehydrogenase subunit L		M. avium		inorganic ion transport and metabolism

**62**	lipolytic enzyme		M. avium/M. smegmatis/ C. glutamicum/ B.bronchiseptica		inorganic ion transport and metabolism
**63**	Cyclododecanone monooxygenase		M. avium/M. smegmatis	oxidation reduction	inorganic ion transport and metabolism
**64**	KtrA		V. parahaemolyticum	pottasium uptake protein	inorganic ion transport and metabolism
**65**	TrkH		T. denticola	Trk system potassium uptake protein	inorganic ion transport and metabolism
**66**	Ferritin		T. denticola		inorganic ion transport and metabolism
**67**	hemerythrin-related		T. denticola	metal ion binding	inorganic ion transport and metabolism
**68**	FeoA		T. denticola	iron ion binding	inorganic ion transport and metabolism
**69**	rhodanese like domain containing protein		T. denticola		inorganic ion transport and metabolism
**70**	peptide ABC transporter		T. denticola		inorganic ion transport and metabolism
**71**	4-hydroxyphenylacetate degradation		E. coli	decarboxylase	IIary metabolites transport and metabolism
**72**	TauD/TfdA		M. avium	taurine catabolism dioxygenase	IIary metabolites transport and metabolism
**73**	carveol dehydrogenase		M. avium/ B. bronchiseptica	dehydrogenase activity	IIary metabolites transport and metabolism
**74**	carotenoid oxygenase		M. avium	oxygenase activity	IIary metabolites transport and metabolism
**75**	arylamine N-acetyltransferase		M. avium/E. coli	acetyltransferase activity	IIary metabolites transport and metabolism
**76**	3-alpha-hydroxysteroid dehydrogenase		M. avium/ M. smegmatis	oxidation reduction	IIary metabolites transport and metabolism
**77**	2,5-dichloro-2,5-cyclohexadiene-1,4-diol dH		M. avium/ M. smegmatis	oxidation reduction	IIary metabolites transport and metabolism

**78**	P450 heme thiolate protein		M.avium		IIary metabolites transport and metabolism
**79**	retinal pigment epithelium membrane protein		M. avium/ M. smegmatis		IIary metabolites transport and metabolism
**80**	linear gramicidin synthetase subunit B		M. avium/ M. smegmatis	isomerase, ligase activiy	IIary metabolites transport and metabolism
**81**	queuine Trna-ribosyltransferase		M. avium/ M. smegmatis	biosynthetic protein	Translation
**82**	selenocysteine-specific translation		M. avium/ M. smegmatis	elogation factor	Translation
**83**	GCN5-related-N-acetyltransferase		M. avium/ M. smegmatis	metabolic process	Translation
**84**	thiopurine S-methyltransferase		M. avium	metabolic process	coenzyme transport and metabolism
**85**	D-isomer specific 2-hydroxyacid dH family		M. avium/ M. smegmatis	oxidoreductase	coenzyme transport and metabolism
**86**	precorrin 6A-synthetase		M. avium/ M. smegmatis		coenzyme transport and metabolism
**87**	glycoxylase family		M. avium/ M. smegmatis		amino acid transport and metabolism
**88**	selenide, water dikinase		M. avium/ M. smegmatis		amino acid transport and metabolism
**89**	gamma-glutamyltransferase		M. avium		amino acid transport and metabolism
**90**	3-isopropylmalate dehydrogenase small su		M. avium/ M. smegmatis	oxidation reduction	amino acid transport and metabolism
**91**	Orn/Lys/Arg decarboxylase		M. avium/ M. smegmatis	decarboxylase	amino acid transport and metabolism
**92**	B12-dependent methionine synthetase		M. avium/ M. smegmatis		amino acid transport and metabolism
**93**	dihydrodipicolinate synthase family protein		M. avium/ M. smegmatis	synthase activity	amino acid transport and metabolism
**94**	cystathionine gamma-lyase		M. avium	lyase activity	amino acid transport and metabolism

**95**	choline dehydrogenase		M. avium/ M. smegmatis	oxidation reduction	amino acid transport and metabolism
**96**	chorismate synthase		T. denticola	synthase activity	amino acid transport and metabolism
**97**	3-phosphoshikinate 1-carboxyviniltransferase		T. denticola		amino acid transport and metabolism
**98**	shikimate kinase		T. denticola	biosynthetic process	amino acid transport and metabolism
**99**	ornithine cyclodeaminase		T. denticola	cyclodeaminase activity	amino acid transport and metabolism
**100**	L-threonine O-3-phosphate decarboxylate		T. denticola	decarboxylase	amino acid transport and metabolism
**101**	aromatic amino acid aminotransferase		S. suis	transferase activity	amino acid transport and metabolism
**102**	Cell surface antigen Sca2		R. africae	autotransportase domain	Unknown

Table S11. Genes found only in “bad bugs” or only in the control species.

COGs	COG description	Found in
COG2264	Ribosomal protein L11 methylase (J)	Y. pestis, Y. pseudotuberculosis, B. bronchiseptica
COG1188	Ribosome-associated heat shock protein implicated in the recycling of the 50S subunit (S4 paralog) (J)	B. bronchiseptica, B. pertussis, Y. pestis, Y. pseudotuberculosis; V. parahaemolyticus, V. cholerae, C. glutamicum, C. Diphtheriae, E. coli, S. Typhi
COG2265 #	SAM-dependent methyltransferases related to tRNA (uracil-5-)-methyltransferase (J)	M. smegmatis, M. avium, M. tuberculosis, C. glutamicum, C. diphtheriae, T. denticola, V. parahaemolyticus, V. cholerae, S. pneumoniae, S. pyogenes, S. agalactiae, S. suis, E. coli, S. dysenteriae, B. pertussis, S. Typhi, S. Schwarzengrund
COG1595 *	DNA-directed RNA polymerase specialized sigma subunit, sigma24 homolog (K)	B. bronchiseptica, B. pertussis, C. glutamicum, M. leprae
COG1508 #	DNA-directed RNA polymerase specialized sigma subunit, sigma54 homolog (K)	Y. pestis, Y. pseudotuberculosis, V. parahaemolyticus, V. cholerae, B. bronchiseptica, B. pertussis, E. coli, S. dysenteriae, T. pallidum, T. Denticola, S. Typhi, S. Schwarzengrund
COG1522	Transcriptional regulators (K)	V. parahaemolyticus, V. cholerae, B. bronchiseptica, B. pertussis, E. coli, Y. pestis, Y. pseudotuberculosis, M. tuberculosis, S. Typhi, S. Schwarzengrund
COG1167	Transcriptional regulators containing a DNA-binding HTH domain and an aminotransferase domain (MocR family) and their eukaryotic orthologs (KE)	
COG1974 *	SOS-response transcriptional repressors (RecA-mediated autopeptidases) (KT)	S. pneumoniae, M. smegmatis, M. tuberculosis, M. avium, M. leprae, C. diphtheriae, B. bronchiseptica, B. pertussis, S. Typhi, S. Schwarzengrund
COG1643	HrpA-like helicases (L)	Y. pseudotuberculosis, Y. pestis, E. coli, S. dysenteriae, B. bronchiseptica, B. pertussis, V. parahaemolyticus, V. cholerae, M. avium, M. smegmatis, C. glutamicum, C. diphtheriae, S. Typhi, S. Schwarzengrund
COG0277 * #	FAD/FMN-containing dehydrogenases (C)	M. smegmatis, M. leprae, M. avium, M. tuberculosis, T. denticola, E. coli, B. bronchiseptica, B. pertussis
COG0247 *	Fe-S oxidoreductase (C)	M. smegmatis, M. leprae, E. coli, C. diphtheriae, S. Typhi, S. Schwarzengrund
COG2225 *	Malate synthase (C)	V. cholerae, V. parahaemolyticus, E. coli, Y. pseudotuberculosis, Y. pestis, S. dysenteriae, M. leprae, S. Typhi, S. Schwarzengrund
COG1902	NADH:flavin oxidoreductases, Old Yellow Enzyme family (C)	B. bronchiseptica, Y. pseudotuberculosis, Y. pestis, C. glutamicum, E. coli, S. Typhi, S.

		Schwarzengrund
COG0778 *	Nitroreductase (C)	S. dysenteriae, E. coli, M. leprae, S. Typhi, S. Schwarzengrund
COG2352 *	Phosphoenolpyruvate carboxylase (C)	M. leprae, M. smegmatis, M. avium, S. suis, S. pneumoniae, S. agalactiae, C. diphtheriae, C. glutamicum, B. pertussis, B. bronchiseptica, S. pyogenes, V. parahaemolyticus, V. cholerae, S. dysenteriae, Y. pseudotuberculosis, S. Typhi, S. Schwarzengrund
COG1600	Uncharacterized Fe-S protein (C)	E. coli, S. dysenteriae, Y. pestis, Y. pseudotuberculosis, V. cholerae, V. parahaemolyticus, S. pyogenes, S. agalactiae, S. suis
COG1062 *	Zn-dependent alcohol dehydrogenases, class III (C)	M. leprae, M. avium, M. tuberculosis, M. smegmatis, B. bronchiseptica, B. pertussis, Y. pseudotuberculosis, Y. pestis, C. Glutamicum, E. coli, S. suis, S. pneumoniae, S. pyogenes, S. Schwarzengrund
COG0239	Integral membrane protein possibly involved in chromosome condensation (D)	
COG0683	ABC-type branched-chain amino acid transport systems, periplasmic component (E)	S. suis, S. pneumoniae, M. smegmatis, S. agalactiae, S. dysenteriae
COG1176	ABC-type spermidine/putrescine transport system, permease component I (E)	V. cholerae, V. parahaemolyticus, E. coli, S. dysenteriae, Y. pestis, Y. pseudotuberculosis, S. pneumoniae, S. pyogenes, S. suis, S. agalactiae, S. Typhi, S. Schwarzengrund
COG1177	ABC-type spermidine/putrescine transport system, permease component II (E)	V. parahaemolyticus, Y. pestis, Y. pseudotuberculosis, T. pallidum, E. coli, S. dysenteriae, B. bronchiseptica, V. cholerae, C. glutamicum, M. smegmatis, S. Typhi, S. Schwarzengrund
COG2049 *	Allophanate hydrolase subunit 1 (E)	B. pertussis, B. bronchiseptica, M. tuberculosis, M. avium, M. smegmatis, M. leprae, E. coli, S. dysenteriae, Y. pseudotuberculosis, Y. pestis, C. glutamicum, V. cholerae, S. Typhi, S. Schwarzengrund
COG1984 *	Allophanate hydrolase subunit 2 (E)	Y. pseudotuberculosis, Y. pestis, E. coli, S. dysenteriae, V. cholerae, V. parahaemolyticus, B. bronchiseptica, B. pertussis, M. tuberculosis, M. avium, M. leprae, S. Typhi, S. Schwarzengrund
COG2303	Choline dehydrogenase and related flavoproteins (E)	
COG0014 *	Gamma-glutamyl phosphate reductase (E)	E. coli, S. agalactiae, Y. pseudotuberculosis, Y. pestis, S. pyogenes, V. cholerae, V. parahaemolyticus, S. pneumoniae, S. suis, B. pertussis, B. bronchispetica, T. pallidum, M. leprae, S. Typhi, S.

		Schwarzengrund
COG0405	Gamma-glutamyltransferase (E)	E. coli, S. dysenteriae, Y. pseudotuberculosis, Y. pestis, V. parahaemolyticus, V. cholerae, B. pertussis, B. bronchiseptica, M. tuberculosis, M. avium, M. smegmatis, C. glutamicum
COG0263 *	Glutamate 5-kinase (E)	M. leprae, M. smegmatis, M. avium, M. tuberculosis, V. cholerae, E. coli, B. bronchiseptica, B. pertussis, Y. pestis, S. pneumoniae, S. suis, S. agalactiae, S. pyogenes, C. glutamicum
COG0665	Glycine/D-amino acid oxidases (deaminating) (E)	B. bronchispetica, B. pertussis, V. parahaemolyticus, S. dysenteriae, E. Coli, S. Typhi, S. Schwarzengrund
COG0346 *	Lactoylglutathione lyase and related lyases (E)	M. leprae, M. avium, S. Schwarzengrund
COG2755 *	Lysophospholipase L1 and related esterases (E)	M. leprae
COG1410 *	Methionine synthase I, cobalamin-binding domain (E)	M. leprae, M. smegmatis, M. avium, M. tuberculosis, B. bronchiseptica, B. pertussis, V. cholerae, V. parahaemolyticus, E. coli, Y. pestis, Y. pseudotuberculosis, S. Typhi, S. Schwarzengrund
COG0347	Nitrogen regulatory protein PII (E)	V. cholerae, E. coli, S. dysenteriae, B. pertussis, B. bronchiseptica, V. parahaemolyticus, Y. pseudotuberculosis, Y. pestis, C. glutamicum, C. diphtheriae, M. tuberculosis, M. smegmatis, S. Typhi, S. Schwarzengrund
COG1280	Putative threonine efflux protein (E)	V. cholerae, Y. pestis, Y. pseudotuberculosis
COG2008 #	Threonine aldolase (E)	M. smegmatis, T. denticola, S. agalactiae, S. suis
COG1762 #	Phosphotransferase system mannitol/fructose-specific IIA domain (Ntr-type) (GT)	T. denticola, V. parahaemolyticus
COG0315	Molybdenum cofactor biosynthesis enzyme (H)	V. cholerae, M. avium, M. tuberculosis, Y. pseudotuberculosis, Y. pestis, E. coli, C. glutamicum, M. smegmatis, C. diphtheriae, S. Typhi, S. Schwarzengrund
COG2896	Molybdenum cofactor biosynthesis enzyme (H)	M. tuberculosis, M. avium, M. smegmatis, C. diphtheriae, C. glutamicum, B. bronchiseptica, B. pertussis, E. coli, S. dysenteriae, V. cholerae, V. parahaemolyticus, Y. pestis, Y. pseudotuberculosis, S. Typhi
COG0303*	Molybdopterin biosynthesis enzyme (H)	M. leprae, M. avium, M. tuberculosis, E. coli, Y. pseudotuberculosis, Y. pestis, B. bronchiseptica, B. pertussis, V. cholerae, C. diphtheriae, C. glutamicum, S. Typhi, S. Schwarzengrund

COG0521 *	Molybdopterin biosynthesis enzymes (H)	M. leprae, M. avium, M. tuberculosis, M. smegmatis, V. cholerae, V. parahaemolyticus, E. coli, S. dysenteriae, B. bronchiseptica,; B. pertussis, Y. pestis, Y. pseudotuberculosis, C. glutamicum
COG0314	Molybdopterin converting factor, large subunit (H)	V. cholerae, V. parahaemolyticus, E. coli, S. dysenteriae, Y. pestis, Y. pseudotuberculosis, B. bronchiseptica, B. Pertussis
COG1977	Molybdopterin converting factor, small subunit (H)	
COG0746	Molybdopterin-guanine dinucleotide biosynthesis protein A (H)	
COG2267 *	Lysophospholipase (I)	M. leprae, M. tuberculosis, M. smegmatis, M. avium, C. diphtheriae, S. Typhi, S. Schwarzengrund
COG0318	Acyl-CoA synthetases (AMP-forming)/AMP-acid ligases II (IQ)	S. Typhi, S. Schwarzengrund
COG2230 *	Cyclopropane fatty acid synthase and related methyltransferases (M)	V. parahaemolyticus, Y. pestis, V. cholerae, Y. pseudotuberculosis, E. coli, S. dysenteriae, S. agalactiae, C. glutamicum, B. bronchiseptica, B. pertussis, M. avium, M. tuberculosis, M. leprae, S. Typhi, S. Schwarzengrund
COG1596	Periplasmic protein involved in polysaccharide export (M)	
COG0810	Periplasmic protein TonB, links inner and outer membranes (M)	S. Typhi, S. Schwarzengrund
COG1247	Sortase and related acyltransferases (M)	E. coli, S. dysenteriae, C. glutamicum, C. diphtheriae, B. bronchiseptica, B. pertussis, V. cholerae, S. Typhi, S. Schwarzengrund
COG2148	Sugar transferases involved in lipopolysaccharide synthesis (M)	V. cholerae, S. pneumoniae, S. suis, S. agalactiae, V. parahaemolyticus, C. glutamicum, S. Typhi, S. Schwarzengrund
COG3206	Uncharacterized protein involved in exopolysaccharide biosynthesis (M)	E. coli, S. dysenteriae, C. glutamicum, V. parahaemolyticus, V. cholerae, S. agalactiae, S. pneumoniae
COG1580	Flagellar basal body-associated protein (N)	B. bronchiseptica, B. pertussis, S. Typhi, S. Schwarzengrund
COG1291	Flagellar motor component (N)	B. bronchiseptica, E. coli, Y. pseudotuberculosis, Y. pestis, S. dysenteriae, V. parahaemolyticus
COG1344	Flagellin and related hook-associated proteins (N)	
COG0643 #	Chemotaxis protein histidine kinase and related kinases (NT)	V. cholerae, Y. pestis, Y. pseudotuberculosis, E. coli, T. denticola, T. Pallidum, S. Typhi, S. Schwarzengrund
COG2201	Chemotaxis response regulator containing a CheY-like receiver domain and a methylesterase domain (NT)	T. pallidum, V. parahaemolyticus, V. cholerae
COG0835 #	Chemotaxis signal transduction	V. parahaemolyticus, V. cholerae,

	protein (NT)	E. coli, Y. pestis, Y. pseudotuberculosis, B. bronchiseptica, T. pallidum, T. denticola, S. Typhi, S. Schwarzengrund
COG0840 #	Methyl-accepting chemotaxis protein (NT)	V. cholerae, V. parahaemolyticus, E. coli, T. denticola, S. dysenteriae
COG1352 #	Methylase of chemotaxis methyl-accepting proteins (NT)	V. cholerae, E. coli, Y. pestis, Y. pseudotuberculosis, B. pertussis, T. denticola, T. pallidum, V. parahaemolyticus, M. smegmatis, M. avium, S. Typhi, S. Schwarzengrund
COG0229	Conserved domain frequently associated with peptide methionine sulfoxide reductase (O)	E. coli, S. dysenteriae, V. parahaemolyticus, V. cholerae, Y. pestis, Y. pseudotuberculosis, S. suis, S. pyogenes, S. pneumoniae, S. Typhi, S. Schwarzengrund
COG4235	Cytochrome c biogenesis factor (O)	E. coli, S. dysenteriae, Y. pseudotuberculosis, Y. pestis, V. cholerae, V. parahaemolyticus
COG1281	Disulfide bond chaperones of the HSP33 family (O)	Y. pestis, Y. pseudotuberculosis, E. coli, S. dysenteriae, V. cholerae, V. parahaemolyticus, B. pertussis, B. bronchiseptica
COG0386 #	Glutathione peroxidase (O)	M. avium, M. smegmatis, E. coli, S. dysenteriae, C. glutamicum, C. diphtheriae, Y. pestis, Y. pseudotuberculosis, S. pyogenes, S. suis, S. pneumoniae, B. pertussis, B. bronchiseptica, T. denticola, S. Typhi, S. Schwarzengrund
COG2360 #	Leu/Phe-tRNA-protein transferase (O)	E. coli, S. dysenteriae, B. pertussis, T. denticola, Y. pestis, Y. pseudotuberculosis, V. cholerae, S. Typhi, S. Schwarzengrund
COG0652 *	Peptidyl-prolyl cis-trans isomerase (rotamase) – cyclophilin family (O)	M. leprae, E. coli, S. dysenteriae, V. parahaemolyticus, V. cholerae, B. pertussis, B. bronchiseptica, Y. pestis, Y. pseudotuberculosis, S. suis, S. pneumoniae, S. Typhi, S. Schwarzengrund
COG1764	Predicted redox protein, regulator of disulfide bond formation (O)	C. glutamicuum, M. smegmatis, V. cholerae, V. parahaemolyticus
COG2518	Protein-L-isoaspartate carboxylmethyltransferase (O)	V. parahaemolyticus, Y. pestis, Y. pseudotuberculosis, V. cholerae, E. coli, S. dysenteriae, B. bronchiseptica, B. pertussis, S. Typhi
COG3118 *	Thioredoxin domain-containing protein (O)	E. coli, S. dysenteriae, Y. pestis, Y. pseudotuberculosis, V. cholerae, V. parahaemolyticus, C. glutamicum, C. diphtheriae, B. bronchiseptica, M. leprae, M. avium, M. tuberculosis, S. pyogenes, S. agalactiae, S. Typhi, S. Schwarzengrund
COG2844	UTP:GlnB (protein PII) uridylyltransferase (O)	V. cholerae, V. parahaemolyticus, E. coli, S. dysenteriae, Y. pseudotuberculosis, Y. pestis, B. bronchiseptica, B. pertussis, M.

		smegmatis, M. avium, M. tuberculosis, C. diphtheriae, C. glutamicum, S. Typhi, S. Schwarzengrund
COG1391 *	Glutamine synthetase adenylyltransferase (OT)	M. leprae, M. tuberculosis, M. smegmatis, M. avium, E. coli, V. cholerae, V. parahaemolyticus, S. dysenteriae, Y. pseudotuberculosis, Y. pestis, C. diphtheriae, B. pertussis, S. Typhi, S. Schwarzengrund
COG0725	ABC-type molybdate transport system, periplasmic component (P)	V. parahaemolyticus, V. cholerae, E. coli, Y. pestis, Y. pseudotuberculosis, S. dysenteriae, C. glutamicum, C. diphtheriae, M. avium, M. tuberculosis, M. smegmatis, S. Typhi, S. Schwarzengrund
COG0600	ABC-type nitrate/sulfonate/bicarbonate transport system, permease component (P)	B. bronchiseptica, B. pertussis, C. glutamicum, V. parahaemolyticus, Y. pestis, Y. pseudotuberculosis, M. avium, S. suis, S. pneumoniae
COG0004	Ammonia permease (P)	V. parahaemolyticus, V. cholerae, M. avium, M. smegmatis, M. tuberculosis, B. bronchiseptica, B. pertussis, C. glutamicum, E. coli, S. dysenteriae, Y. pestis, Y. pseudotuberculosis, S. Typhi, S. Schwarzengrund
COG1393 *	Arsenate reductase and related proteins, glutaredoxin family (P)	E. coli, S. dysenteriae, Y. pestis, Y. pseudotuberculosis, V. cholerae, V. parahaemolyticus, M. avium, M. smegmatis, M. leprae, B. pertussis, B. bronchiseptica, S. agalactiae, S. pyogenes, C. glutamicum, S. Typhi, S. Schwarzengrund
COG0704	Phosphate uptake regulator (P)	
COG0855 *	Polyphosphate kinase (P)	M. leprae, M. smegmatis, M. tuberculosis, M. avium, B. pertussis, B. bronchiseptica, V. parahaemolyticus, Y. pestis, Y. pseudotuberculosis, S. dysenteriae, E. coli, S. Typhi, S. Schwarzengrund
COG2897 *	Rhodanese-related sulfurtransferase (P)	M. leprae, M. avium, M. tuberculosis, B. pertussis, B. bronchiseptica, C. diphtheriae, C. glutamicum, S. dysenteriae, E. coli, V. parahaemolyticus, V. cholerae, Y. pestis, S. Typhi, S. Schwarzengrund
COG0659 *	Sulfate permease and related transporters (MFS superfamily) (P)	M. tuberculosis, M. leprae, M. avium, M. smegmatis, Y. pestis, Y. pseudotuberculosis, C. diphtheriae, C. glutamicum, S. Typhi, S. Schwarzengrund
COG0179 *	2-keto-4-pentenoate hydratase/2-oxohepta-3-ene-1,7-dioic acid hydratase (catechol pathway) (Q)	M. leprae, M. tuberculosis, S. smegmatis, E. coli, S. dysenteriae, Y. pseudotuberculosis, Y. pestis, B. bronchiseptica, B. pertussis, C. glutamicum, V. parahaemolyticus, V. cholerae, S. Typhi, S. Schwarzengrund

COG3127	Predicted ABC-type transport system involved in lysophospholipase L1 biosynthesis, permease component (Q)	E. coli, S. dysenteriae, Y. pestis, Y. pseudotuberculosis, V. parahaemolyticus, V. cholerae, B. pertussis, B. bronchiseptica, S. Typhi, S. Schwarzengrund
COG0664 *	cAMP-binding proteins – catabolite gene activator and regulatory subunit of cAMP-dependent protein kinases (T)	M. leprae, M. smegmatis, M. avium, M. tuberculosis, C. glutamicum, C. diphtheriae, Y. pestis, Y. pseudotuberculosis, E. coli, S. dysenteriae, V. cholerae, V. parahaemolyticus, S. Typhi, S. Schwarzengrund
COG5001 *	Predicted signal transduction protein containing a membrane domain, an EAL and a GGDEF domain (T)	M. leprae, M. avium, M. smegmatis, V. cholerae, V. parahaemolyticus, Y. pestis, Y. pseudotuberculosis, B. bronchiseptica, B. pertussis, E. coli, C. glutamicum, S. Typhi, S. Schwarzengrund
COG0394	Protein-tyrosine-phosphatase (T)	V. cholerae, V. parahaemolyticus, B. bronchiseptica, B. pertussis, S. agalactiae, S. pyogenes, S. pneumoniae, S. suis
COG3852 #	Signal transduction histidine kinase, nitrogen specific (T)	B. bronchiseptica, B. pertussis, Y. pestis, Y. pseudotuberculosis, E. coli, S. dysenteriae, V. cholerae, V. parahaemolyticus, M. avium, M. tuberculosis, T. denticola, T. pallidum, S. suis, S. Typhi, S. Schwarzengrund
COG1253	Hemolysins and related proteins containing CBS domains (R)	E. coli, S. dysenteriae, Y. pestis, Y. pseudotuberculosis, V. cholerae, V. parahaemolyticus, S. pyogenes, S. suis, S. agalactiae, S. pneumoniae, S. Typhi, S. Schwarzengrund
COG0714 *	MoxR-like ATPases (R)	M. leprae
COG1741	Pirin-related protein (R)	V. cholerae, V. parahaemolyticus, Y. pseudotuberculosis, Y. pestis, M. smegmatis, M. avium, B. bronchiseptica, B. pertussis, S. dysenteriae, E. coli, S. Typhi, S. Schwarzengrund
COG0388 *	Predicted amidohydrolase (R)	M. leprae, M. smegmatis, V. parahaemolyticus, V. cholerae, B. pertussis, B. bronchiseptica, Y. pseudotuberculosis, Y. pestis, S. Typhi, S. Schwarzengrund
COG2081	Predicted flavoproteins (R)	E. coli, Y. pestis, V. parahaemolyticus, V. cholerae, B. bronchiseptica, B. pertussis, S. pyogenes, S. pneumoniae, S. agalactiae, S. suis, S. Typhi, S. Schwarzengrund
COG1694	Predicted pyrophosphatase (R)	S. agalactiae
COG1611 *	Predicted Rossmann fold nucleotide-binding protein (R)	M. leprae, E. coli, S. dysenteriae, Y. pseudotuberculosis, Y. pestis, V. Cholerae, S. Typhi, S. Schwarzengrund
COG0523	Putative GTPases (G3E family) (R)	E. coli, S. dysenteriae, Y. pseudotuberculosis, Y. pestis, V. cholerae, M. smegmatis, B. bronchiseptica, B. pertussis, S.

		Typhi, S. Schwarzengrund
COG2334	Putative homoserine kinase type II (protein kinase fold) (R)	
COG1540 *	Uncharacterized proteins, homologs of lactam utilization protein B (R)	M. leprae, M. avium, M. smegmatis, C. glutamicum, B. pertussis, B. bronchiseptica, Y. pestis, E. coli, S. dysenteriae, V. parahaemolyticus, V. cholerae, S. Typhi, S. Schwarzengrund
COG0397	Uncharacterized conserved protein (S)	M. aviu;, M. smegmatis, C. glutamicum, Y. pseudotuberculosis, Y. pestis, V. cholerae, E. coli, S. dysenteriae
COG1576	Uncharacterized conserved protein (S)	
COG2127 *	Uncharacterized conserved protein (S)	M. leprae, M. smegmatis, M. avium, M. tuberculosis, C. diphtheriae, C. glutamicum, S. Typhi, S. Schwarzengrund
COG2606 * #	Uncharacterized conserved protein (S)	M. leprae, T. denticola, S. Typhi, S. Schwarzengrund
COG2983	Uncharacterized conserved protein (S)	Y. pestis, Y. pseudotuberculosis, E. coli, S. dysenteriae, B. bronchiseptica, B. pertussis, V. cholerae, V. parahaemolyticus, M. avium, M. smegmatis, M. tuberculosis, C. glutamicum, C. diphtheriae, S. Typhi, S. Schwarzengrund
COG1671	Uncharacterized protein conserved in bacteria (S)	Y. pestis, Y. pseudotuberculosis, S. dysenteriae, V. parahaemolyticus, V. cholerae, T. denticola, T. pallidum, S. Typhi, S. Schwarzengrund
COG 3024	Uncharacterized protein conserved in bacteria (S)	E. coli, S. dysenteriae, Y. pestis, S. Typhi, S. Schwarzengrund

Table S12. Set of 100 COGs that are lost in obligate intracellular bacteria.
#: genes found in *T. denticola*, *: genes found in *M. leprae*

Genes	Function
COG1167	Transcriptional regulators containing a DNA-binding HTH domain and an aminotransferase domain (MocR family) and their eukaryotic orthologs (KE)
COG0239	Integral membrane protein possibly involved in chromosome condensation (D)
COG2303	Choline dahydrogenase and related flavoproteins (E)
COG1977	Molybdopterin converting factor, small subunit (H)
COG0746	Molybdopterin-guanine dinucleotide biosynthesis protein A (H)
COG1596	Periplasmic protein involved in polysaccharide export (M)
COG1344	Flagellin and related hook-associated proteins (N)
COG0704	Phosphate uptake regulator (P)
COG2334	Putative homoserine kinase type II (protein kinase fold) (R)
COG1576	Uncharacterized conserved protein (S)

Table S13. Among the set of 100 genes, ten genes were not detected in any of our 24 species.
