# Supplementary figures and images for: Genomes of the Most Dangerous Epidemic Bacteria Have a Virulence Repertoire Characterized by Fewer Genes but More Toxin-Antitoxin Modules
Source: PLoS One. 2011 Mar 18;6(3):e17962. doi: 10.1371/journal.pone.0017962 (PMC3060909; doi:10.1371/journal.pone.0017962)

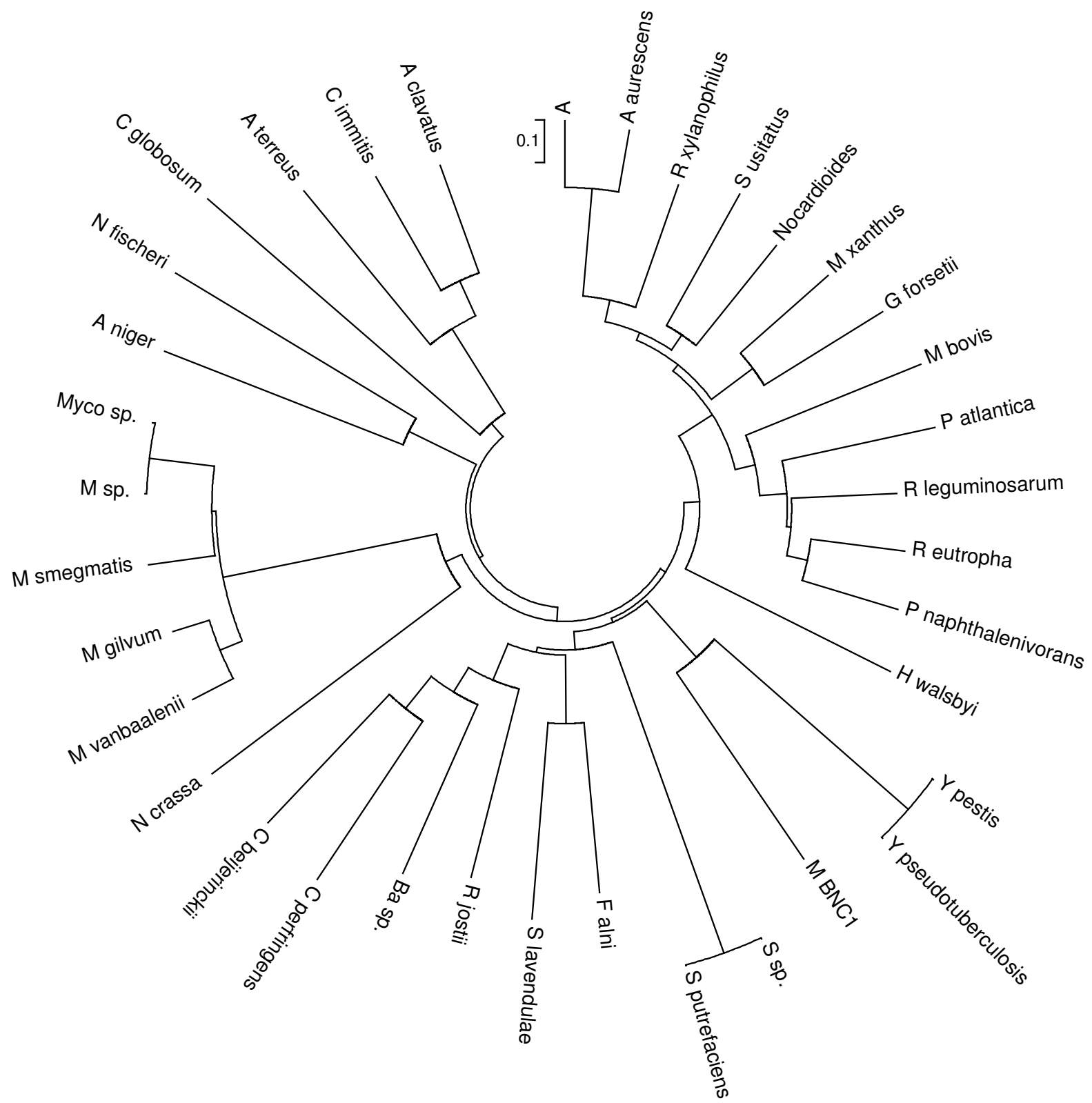

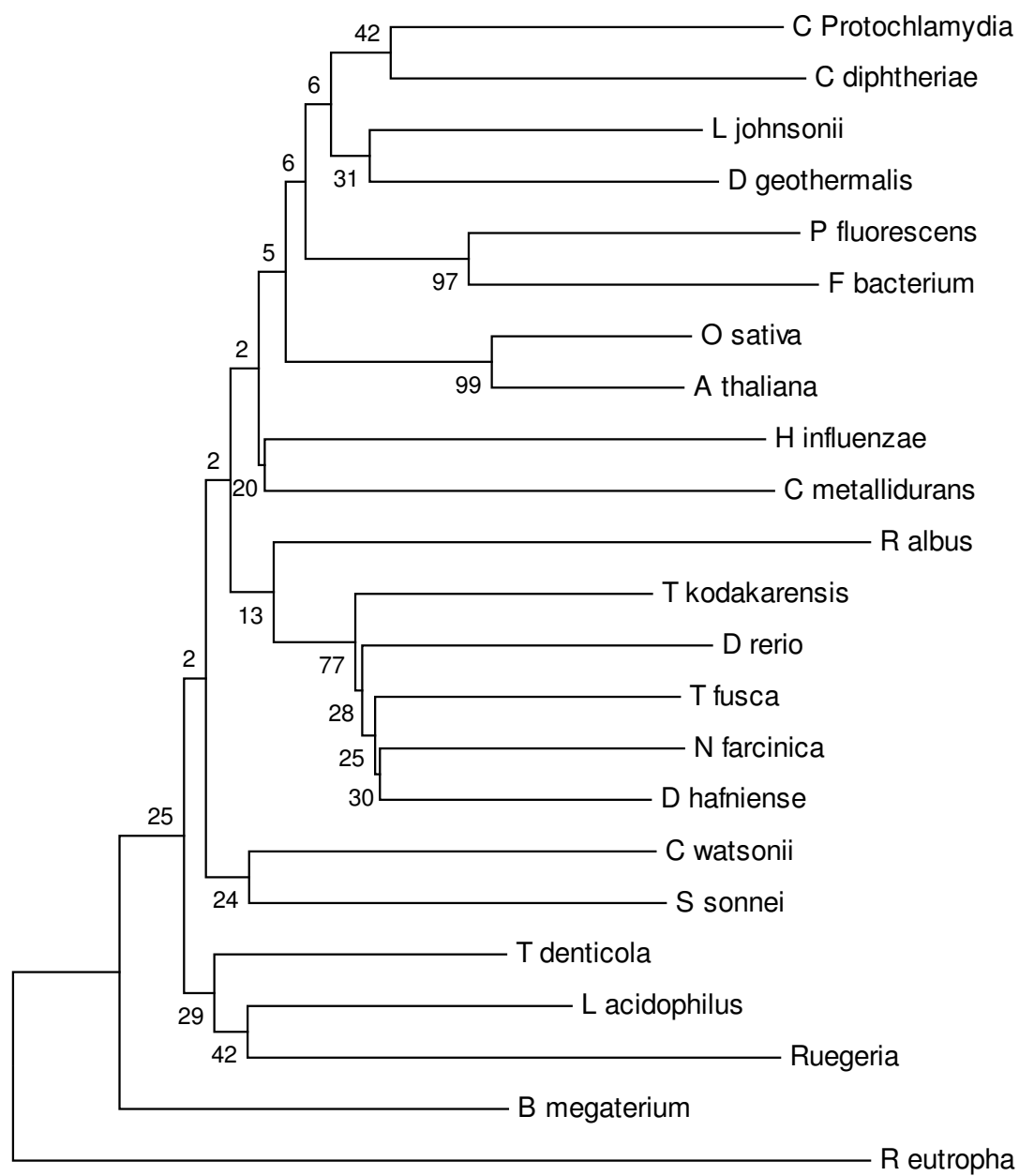

0.2

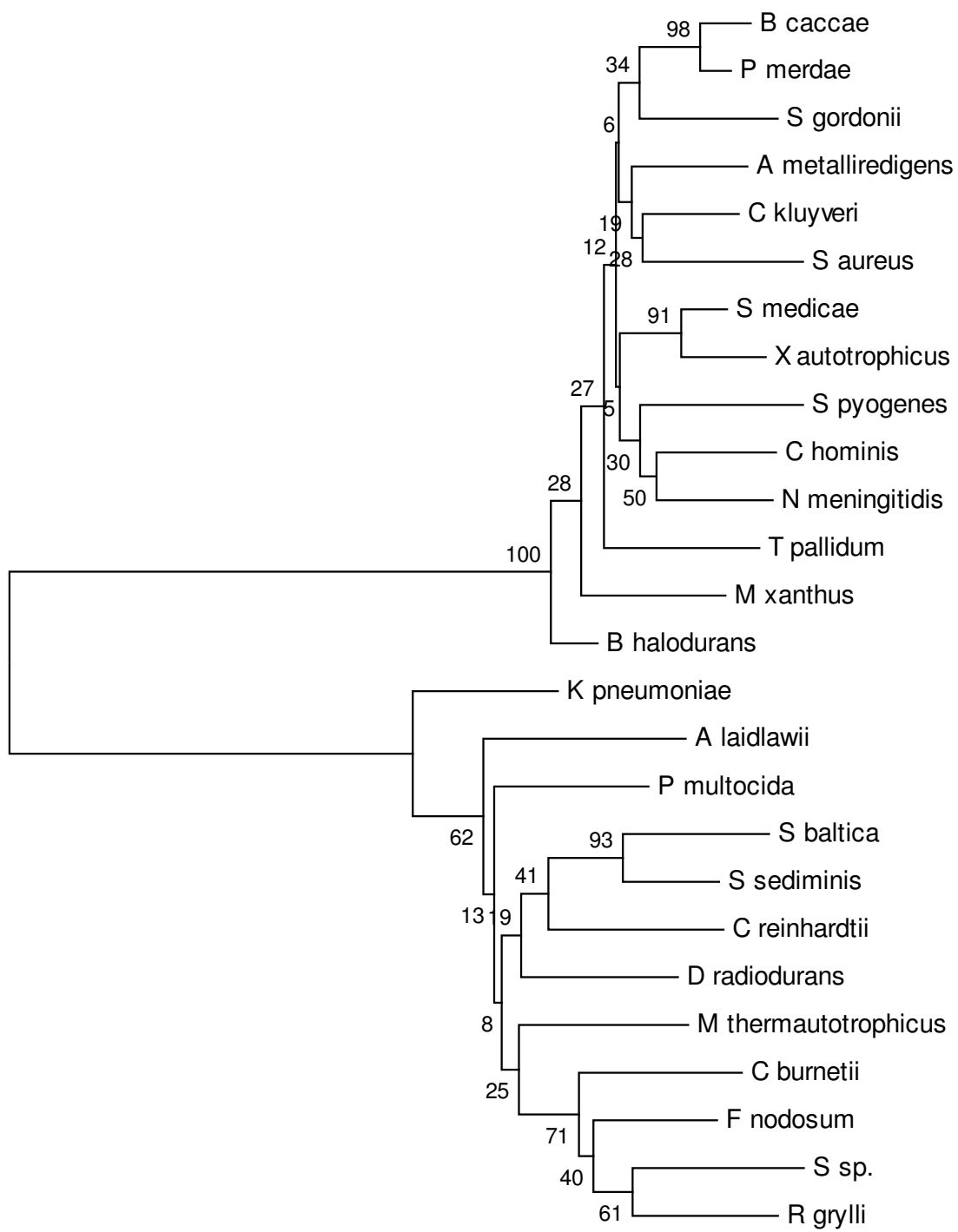

0.2

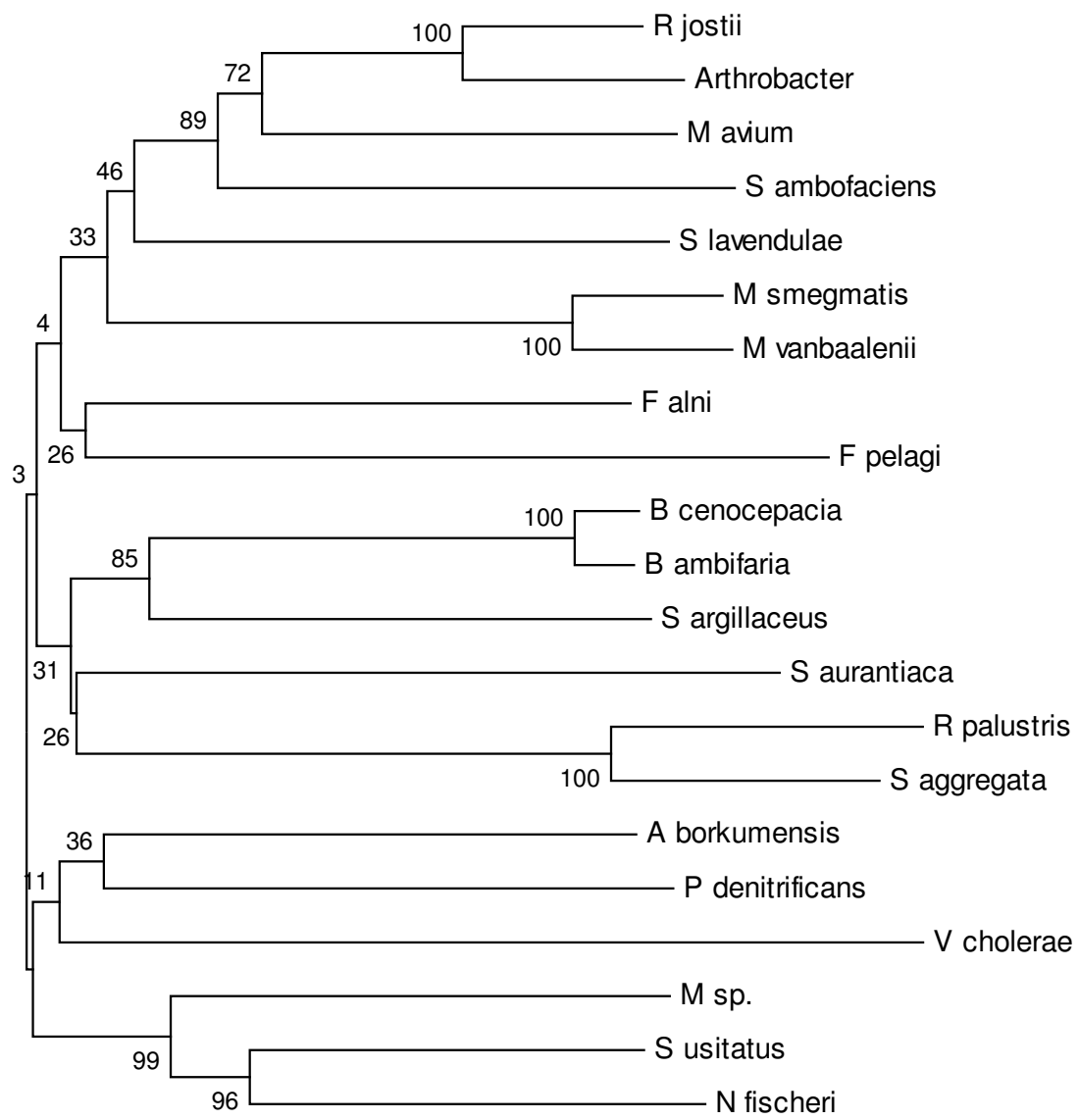

0.1

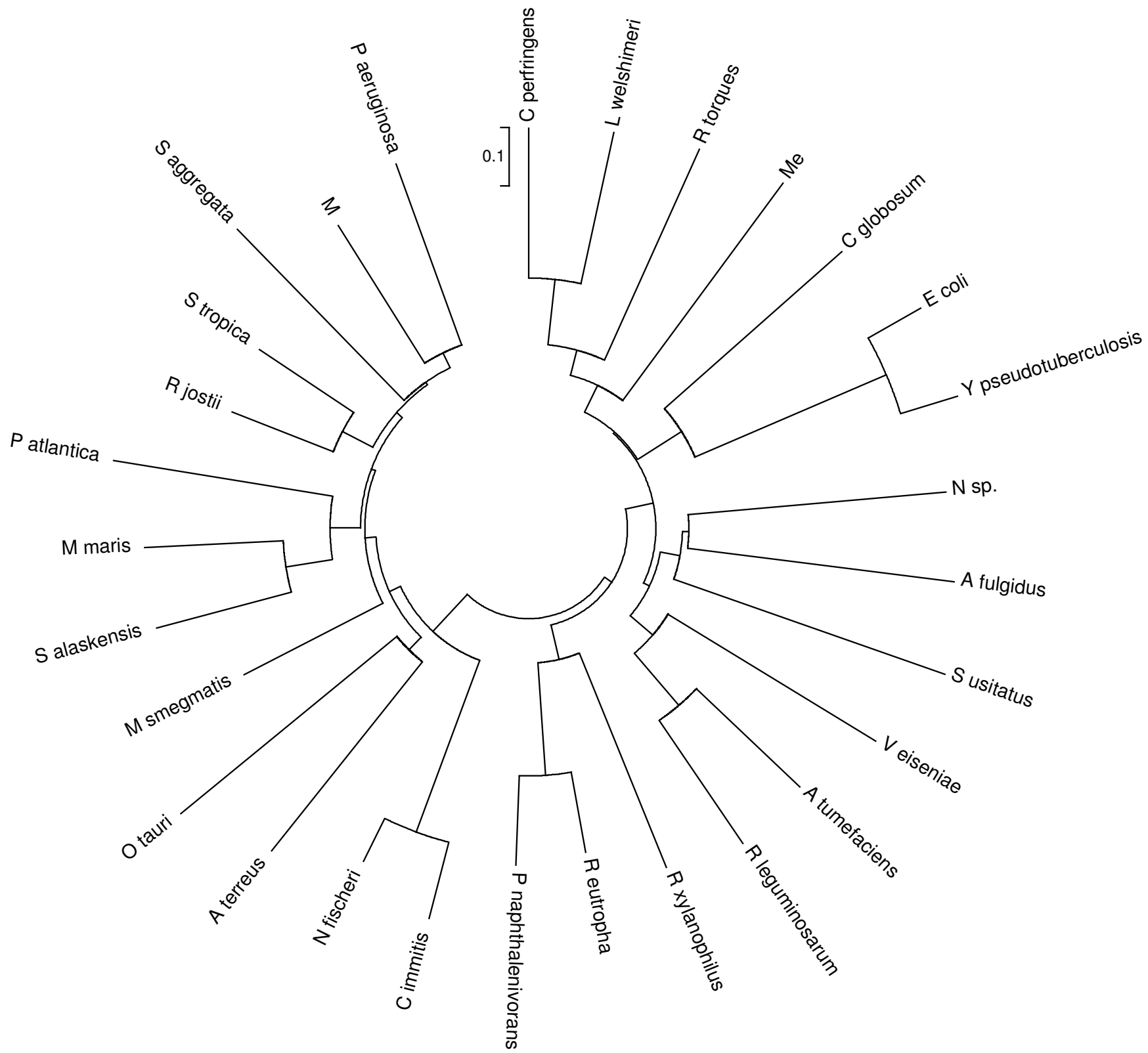

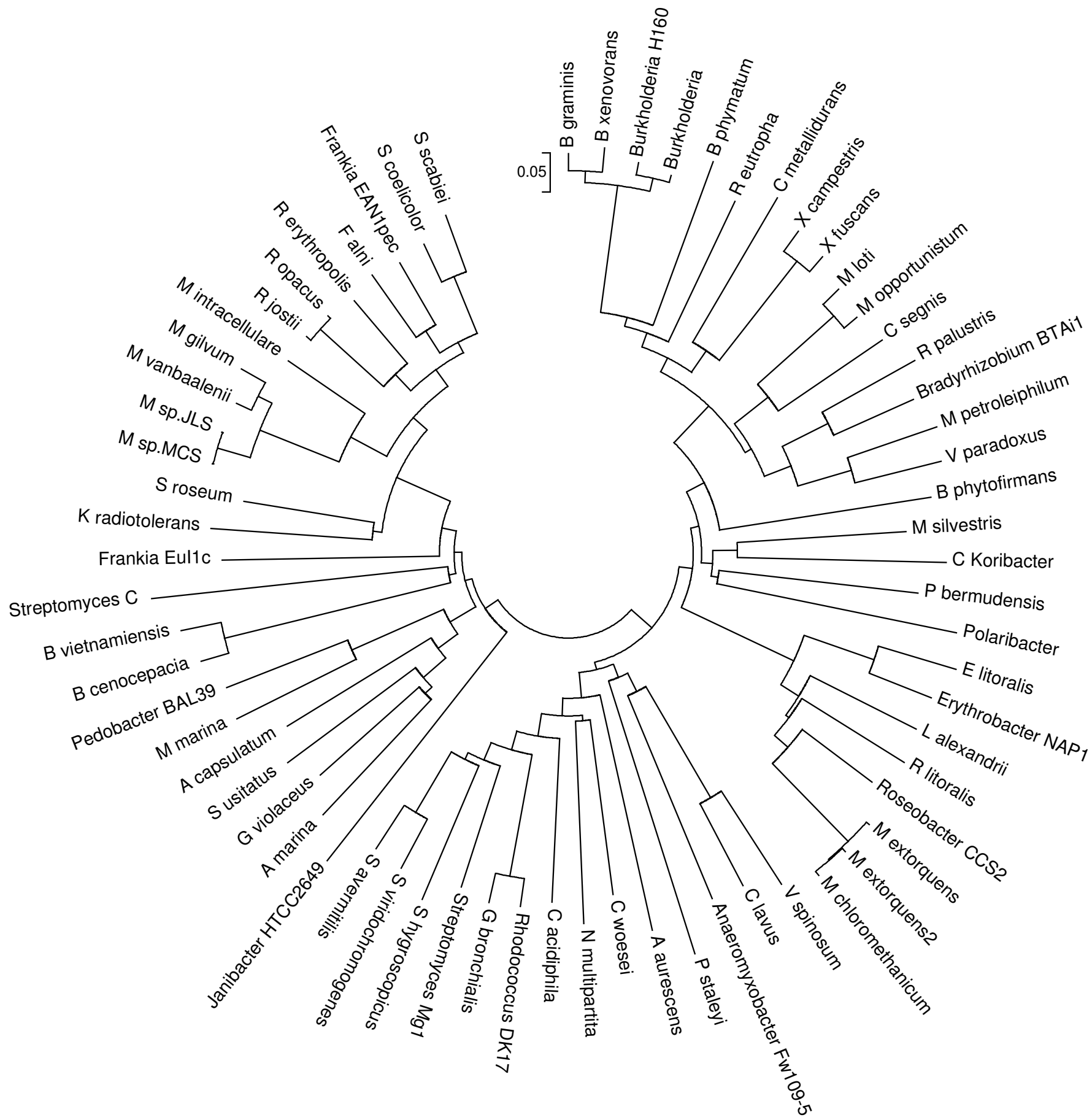

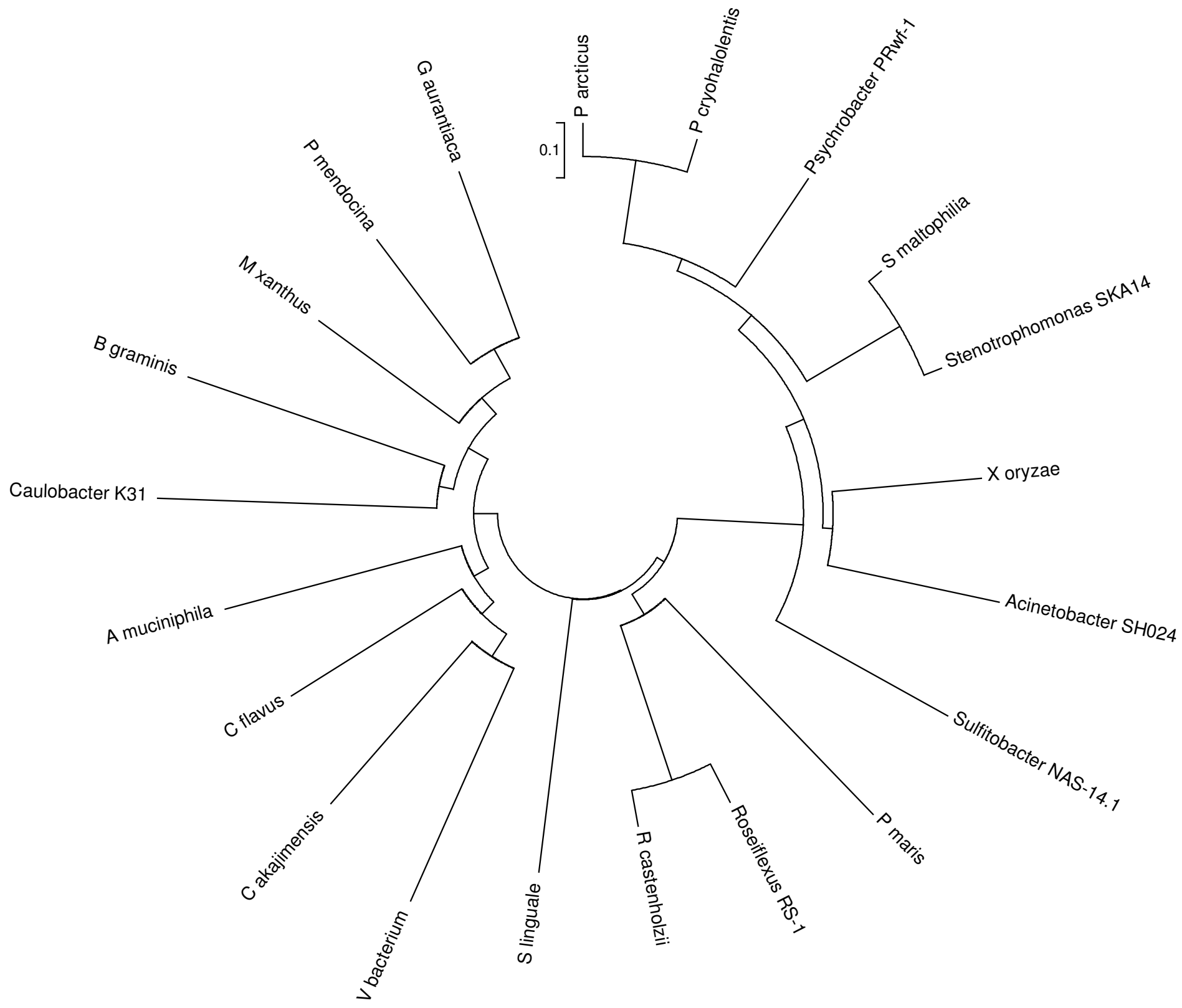

Supplement: Supporting Information S3 — Phylogenetic trees of specific genes and HGT. (PDF) [file pone.0017962.s003.pdf]
